# Scalarane Sesterterpenoids with Antibacterial and Anti-Proliferative Activities from the Mushroom *Neonothopanus* *nambi*

**DOI:** 10.3390/molecules26247667

**Published:** 2021-12-17

**Authors:** Awat Wisetsai, Ratsami Lekphrom, Sureeporn Bua-art, Thanapat Suebrasri, Sophon Boonlue, Sarawut Tontapha, Vittaya Amornkitbamrung, Thanaset Senawong, Florian T. Schevenels

**Affiliations:** 1 Department of Chemistry, Center for Innovation in Chemistry, Faculty of Science, Khon Kaen University, Khon Kaen 40002, Thailand; w.awat@kkumail.com (A.W.); ratsami@kku.ac.th (R.L.); 2 Applied Taxonomic Research Center, Faculty of Science, Khon Kaen University, Khon Kaen 40002, Thailand; 3 Plant Pathology Research Group Plant Protection Research and Development Office, Department of Agriculture, Bangkok 10900, Thailand; suree.suli@gmail.com; 4 Faculty of Medical Science, Nakhon Ratchasima College, Nakhon Ratchasima 30000, Thailand; s.thanapat@kkumail.com; 5 Department of Microbiology, Faculty of Science, Khon Kaen University, Khon Kaen 40002, Thailand; bsopho@kku.ac.th; 6 Integrated Nanotechnology Research Center, Department of Physics, Faculty of Science, Khon Kaen University, Khon Kaen 40002, Thailand; s.tontapha@gmail.com (S.T.); vittaya@kku.ac.th (V.A.); 7 Department of Biochemistry, Faculty of Science, Khon Kaen University, Khon Kaen 40002, Thailand; sthanaset@kku.ac.th

**Keywords:** *Neonothopanus* *nambi*, scalarane, sesterterpenoid, antibacterial, anti-proliferative

## Abstract

Seven undescribed scalarane sesterterpenoids, nambiscalaranes B–H (**1**–**7**), together with two known compounds, nambiscalarane (**8**) and aurisin A (**9**) were isolated from the cultured mycelium of the luminescent mushroom *Neonothopanus nambi*. Their structures were elucidated by thorough analysis of their 1D and 2D NMR spectroscopic data. The absolute configurations of **1**–**8** were determined by electronic circular dichroism (ECD) calculations and optical rotation measurements. The isolated sesterterpenoids were evaluated against A549, HT29, HeLa, and HCT-116 cancer cell lines, and against five bacterial strains. Compounds **3**, **5**, and **7** showed strong cytotoxicity against HCT-116 cell line, with IC_50_ values ranging from 13.41 to 16.53 µM, and showed no cytotoxicity towards *Vero* cells. Moreover, compound **8** inhibited the growth of *Bacillus subtilis* with a MIC value of 8 µg/mL, which was equivalent to the MIC value of the standard kanamycin.

## 1. Introduction

The luminescent mushroom *Neonothopanus nambi* belongs to the Omphalotaceae family. It is known as ‘Hed Ruang Sang Sirin-ratsami’ or ‘Sirin-ratsami mushroom’ in Thai [1]. Previous agricultural studies have shown that *N. nambi* can induce systemic resistance against root-knot nematodes such as *Meloidogyne incognita* in tomato plants [2,3]. *N. nambi* produces diverse secondary metabolites that seemed to greatly vary in nature based on the conditions of its culture (Figure S1 and Table S1). Our previous work showed that potato dextrose broth led to the production of sesquiterpenoids (and dimers) as well as terphenyls and benzoquinones [1,4]. Yeast malt agar conditions led to the isolation of more diverse secondary metabolites. One of them was a scalarane sesterterpenoid, named nambiscalarane (**8**) [5].

Sesterterpenoids are a relatively small family of terpenoids. Among this family, the carbocyclic core can greatly vary. Scalaranes are a rare group of sesterterpenoids with a 6/6/6/6 tetracyclic skeleton. Its first member was discovered from the marine sponge *Cacospongia scalaris* in 1972 [6]. Scalaranes display a wide range of biological properties, including antifeedant, antimicrobial, antifungal, antitumor, cytotoxic, and anti-HIV activities [7,8,9,10,11,12,13,14,15,16,17]. In this work, the mycelium of *N. nambi* was cultured in malt extract broth. Due to its potential in agriculture, we seek to fully understand the scope of the secondary metabolites it can produce. The culture medium greatly affected the production of secondary metabolites, as seven new scalarane sesterterpenenoids, named nambiscalaranes B–H (**1**–**7**), were isolated, along with nambiscalarane (**8**) and aurisin A (**9**). All the isolated sesterterpenoids were tested for their cytotoxic activities against A549, HT29, HeLa, and HCT-116 cancer cell lines. In addition, they were screened for antibacterial activities against several Gram-positive and Gram-negative bacteria.

## 2. Materials and Methods

### 2.1. General Experimental Procedure

Optical rotations were measured with a JASCODIP-1000 digital polarimeter (JASCO Inc., Tokyo, Japan). UV and ECD spectra were recorded using a JASCO J-810 apparatus. IR analyses were performed using a Bruker Tenser 27 spectrophotometer (Bruker, Karlsruhe, Germany). NMR spectra were recorded on a Varian Mercury Plus 400 spectrometer (Varian Inc., Palo Alto, CA, USA) or on a Bruker Avance 400 NMR spectrometer (Bruker, Karlsruhe, Germany) using CDCl_3_, CD_3_OD and CD_3_CN as solvents. The residual peaks of these solvents were used as internal references. The HRESITOFMS were carried out on a Bruker micrOTOF mass spectrometer (Brucker, Karlsruhe, Germany). Column chromatography was carried out on MERCK silica gel 60 (230–400 mesh) (Merck, Darmstadt, Germany). Thin-layer chromatography was carried out with pre-coated MERCK silica gel 60 PF254 (Merck, Darmstadt, Germany); the spots were visualized under UV light (254 and 365 nm) and further stained by spraying *p*-anisaldehyde and then heated until charred. 

### 2.2. Fungus Material

The luminescent mushroom was collected in 2003 from the Plant Genetic Conservation Project under Royal Initiation by Her Royal Highness Princess Maha Chakri Sirindhorn at Kok Phutaka area, Wiang Kao District, Khon Kaen Province, and was identified by Prof. Weerasak Saksirirat as *N. nambi*. The voucher specimen (PW2) [4] was deposited at the Department of Plant Science and Agricultural Resources, Faculty of Agriculture, Khon Kaen University, Khon Kaen, Thailand. The mushroom was cultivated on malt extract broth without shaking with 2 h of light per day at 25 °C for 30 days.

### 2.3. Extraction and Isolation

The cultured mycelium of *N. nambi* (375.7 g) was ground to powder and then extracted at room temperature with EtOAc (3 × 3 L). Removal of solvent under reduced pressure gave the crude EtOAc (82.74 g) extract. The crude EtOAc extract led to the isolation of 36.97 g of **9** as yellow crystals after crystallization from EtOAc. The residue (45.7 g) was obtained from evaporation of the filtrate and chromatographed over silica gel flash column chromatography (FCC), eluting with a gradient system of hexanes:EtOAc and EtOAc:MeOH to afford six fractions, EF1–EF6. Fraction EF1 was separated over silica gel FCC, eluting with a gradient system of hexanes:EtOAc (95:5 to 80:20) to give three subfractions, EF1.1–EF1.3. Subfraction EF1.2 was purified by silica gel FCC, eluting with an isocratic system of hexanes:CH_2_Cl_2_ (25:75) to give **1** (8.0 mg) as a yellow amorphous powder. Fraction EF3 was purified by silica gel FCC, using a gradient elution of hexanes:EtOAc to EtOAc, to give **8** (48.0 mg), **3** (8.5 mg), and **2** (15.0 mg) as pale yellow oils, as well as **6** (5.3 mg) and **5** (4.0 mg) as white viscous oils. Fraction EF4 was subjected to silica gel FCC, eluting with an isocratic system of hexanes:EtOAc (85:15) to obtain two subfractions, EF4.1 and EF4.2. EF4.1 was then purified by FCC, eluting with MeOH:CH_2_Cl_2_ (5:95) to give **4** (10.8 mg) as a white viscous oil and **7** (3.7 mg) as a yellow viscous oil.

Nambiscalarane B (**1**): yellow amorphous powder; [α]^25^_D_ +56.6 (c 0.2, CHCl_3_); UV/Vis (MeOH) λ_max_ (log ε) 201 (3.78) nm; ECD λ_max_ (Δε) 241 (+2.71), 220 (−5.61), 203 (+6.48) nm; ^1^H and ^13^C NMR data, Table 1 and Table 2; IR (neat) ν_max_ 3359, 2921, 2851, 1658, 1542, 1467, 1422, 1260, 1023, 862, 797, 699, 659 cm^−1^; HRESIMS *m*/*z* 369.2788 ([M − H]^−^ (C_25_H_38_O_2_, calcd. 369.2799).

Nambiscalarane C (**2**): pale yellow oil; [α]^25^_D_ +50.0 (c 0.2, CHCl_3_); UV/Vis (MeOH) λ_max_ (log ε) 217 (3.92) nm; ECD λ_max_ (Δε) 240 (+1.44), 218 (−11.29) nm; ^1^H and ^13^C NMR data, Table 1 and Table 2; IR (neat) ν_max_ 3421, 2926, 2859, 1731, 1672, 1538, 1461, 1369, 1259, 1090, 1032, 1021, 799 cm^−1^; HRESIMS *m*/*z* 469.2959 ([M − C_3_H_4_O_4_]^−^ (C_27_H_39_O_4_, calcd. 427.2854).

Nambiscalarane D (**3**): pale yellow oil; [α]^25^_D_ +25.7 (c 0.2, CHCl_3_); UV/Vis (MeOH) λ_max_ (log ε) 217 (3.92) nm; ECD λ_max_ (Δε) 218 (−16.44), 200 (+2.10) nm; ^1^H and ^13^C NMR data, Table 1 and Table 2; IR (neat) ν_max_ 3431, 2925, 2856, 1727, 1656, 1537, 1458, 1368, 1247, 1172, 1143, 1087, 1022, 800 cm^−1^; HRESIMS *m*/*z* 553.3176 ([M − H]^−^ (C_33_H_45_O_7_, calcd. 553.317).

Nambiscalarane E (**4**): white viscous oil; [α]^25^_D_ +32.0 (c 0.2, CHCl_3_); UV/Vis (MeOH) λ_max_ (log ε) 217 (4.00) nm; ECD λ_max_ (Δε) 222 (−15.27), 202 (+4.88) nm; ^1^H and ^13^C NMR data, Table 1 and Table 2; IR (neat) ν_max_ 3359, 2960, 2923, 2852, 1713, 1657, 1466, 1394, 1087, 1017, 791 cm^−1^; HRESIMS *m*/*z* 511.3081 ([M − H]^−^ (C_31_H_43_O_6_, calcd. 511.3065).

Nambiscalarane F (**5**): white viscous oil; [α]^25^_D_ +48.0 (c 0.2, CHCl_3_); UV/Vis (MeOH) λ_max_ (log ε) 202 (4.02), 219 (3.95) nm; ECD λ_max_ (Δε) 228 (−16.48) nm; ^1^H and ^13^C NMR data, Table 1 and Table 2; IR (neat) ν_max_ 3408, 2923, 2854, 1728, 1649, 1459, 1368, 1287, 1244, 1146, 1131, 1084, 1024, 971 cm^−1^; HRESIMS *m*/*z* 569.3127 ([M − H]^−^ (C_33_H_45_O_8_, calcd. 569.3120).

Nambiscalarane G (**6**): white viscous oil; [α]^25^_D_ +50.0 (c 0.2, CHCl_3_); UV/Vis (MeOH) λ_max_ (log ε) 202 (4.02) nm; ECD λ_max_ (Δε) 242 (+2.04), 217 (−5.53), 204 (+9.90) nm; ^1^H and ^13^C NMR data, Table 1 and Table 2; IR (neat) ν_max_ 3396, 2963, 1735, 1651, 1543, 1460, 1375, 1260, 1089, 1032 cm^−1^; HRESIMS *m*/*z* 569.3122 ([M − H]^−^ (C_33_H_45_O_8_, calcd. 569.3120).

Nambiscalarane H (**7**): yellow viscous oil; [α]^25^_D_ +38.0 (c 0.2, CHCl_3_); UV/Vis (MeOH) λ_max_ 203 (3.60) nm; ECD λ_max_ (Δε) 226 (−0.31), 212 (+2.36) nm; ^1^H and ^13^C NMR data, Table 1 and Table 2; IR (neat) ν_max_ 3411, 2953, 2918, 2850, 1730, 1651, 1615, 1460, 1370, 1245, 1172, 1142, 1084, 1023, 946, 734 cm^−1^; HRESIMS *m*/*z* 585.3077 ([M − H]^−^ (C_33_H_45_O_9_, calcd. 585.3096).

Nambiscalarane (**8**): pale yellow oil; [α]^25^_D_ +68.0 (c 0.2, CHCl_3_); UV/Vis (MeOH) λ_max_ 220 (4.17) nm; ECD λ_max_ (Δε) 218 (−13.39), 200 (+1.17) nm; Key NMR spectra are provided in Supporting information (Figures S76 and S78); HRESIMS *m*/*z* 553.3185 ([M − H]^−^ (C_33_H_45_O_7_, calcd. 553.3171), 509.3297 [M − CO_2_]^−^ (C_32_H_45_O_5_, calcd. 509.3272). 

### 2.4. ECD Calculations

Preliminary conformational analyses were carried out using HyperChem software, Hypercube Inc., Gainesville, FL, USA. For theoretical ECD spectra, the possible configurations of compound **2** were established for both geometrical optimizations and electronic excited calculations. Geometrical optimizations of the structure were taken under density functional theory (DFT) calculations. These calculations were performed with hybrid density functional B3LYP, and using 6–311G(d,p) to diffuse basis set. In the single point energy calculations, the vertical transition energies to the valence excited-states were computed with the time-dependent density functional theory (TD-DFT) method using the long-range corrected functional CAM-B3LYP at the 6–311++G(d,p) level (σ = 0.40). The bulk solvent effects were evaluated using the Conductor-like Polarizable Continuum Model (C-PCM). All calculations were performed with the Gaussian09 program [17].

### 2.5. Antibacterial Assay

Five microorganism cultures (Methicillin resistant *S. aureus* DMST 20654, *S. aureus* ATCC 25923, *S. sonnei* ATCC 11060, *B. subtilis* ATCC 6633, and *B. cereus* ATCC 11778) were used. The experiments were performed at the Department of Microbiology, Faculty of Science, Khon Kaen University, Thailand. The antibacterial assay was performed as recommended by the Clinical and Laboratory Standards Institute [18]. The standard drugs kanamycin and chloramphenicol were used as positive controls.

### 2.6. Antiproliferative Activity Assay

The anti-proliferative effect on cancer cells was evaluated by MTT (3-(4,5-dimethylthiazol-2-yl)-2,5-diphenyltetrazolium bromide) assay. Cells (8 × 10^3^ cell/well) were seeded onto 96-well plates and incubated for 24 h to allow adherence. After 24 h, the cells were exposed to increasing concentrations (3.12, 6.25, 12.5, 25, 50, and 100 µg/mL) of pure metabolites in a mixture of DMSO and ethanol (1:1) for 72 h. Control groups were treated with a mixture of DMSO and ethanol (1:1). After the indicated time, the medium was replaced with 110 µL of fresh medium containing MTT (0.5 mg/mL in PBS) (Sigma Chemical Co., St Louis, MO, USA) and incubated for 2 h. The formazan formed after conversion of MTT was dissolved in DMSO. The absorbance of formazan was measured with a microplate reader (Bio-Rad Laboratories, Hercules, CA, USA) at the wavelength of 550 nm with a reference wavelength of 655 nm. The percentage of viable cells which corresponds to the production of formazan was calculated using the following formula [19].

%cell viability = [Sample (A550 − A655)/Control (A550 − A655)] × 100(1)

## 3. Results

### 3.1. Isolated Compounds from N. nambi

**Figure 1:**
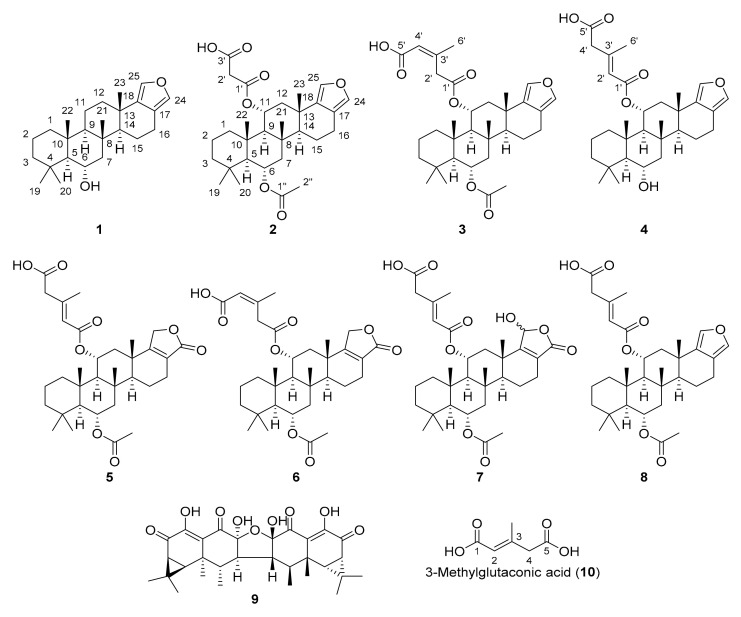
Structures of the isolated compounds from *N. nambi* (**1**–**9**) and 3-methylglutaconic acid (**10**).

The dried cultured mycelium of *N. nambi* was extracted with ethyl acetate (EtOAc). Separation of the crude EtOAc extract via column chromatography (CC) yielded eight sesterterpenoids (**1**–**8**) and aurisin A (**9**) [4]. Their structures were established by thorough analysis of spectroscopic evidence. They are depicted in Figure 1. The data obtained were compared with published values of similar compounds. The absolute configurations of **1**–**7** were determined by a combination of electronic circular dichroism (ECD) calculations and optical rotation ([α]_D_) measurements. The absolute configuration of the known sesterterpenoid nambiscalarane (**8**), previously unspecified [5], was also determined in a similar manner.

### 3.2. Structural Characterization of the New Compounds

**Figure 2:**
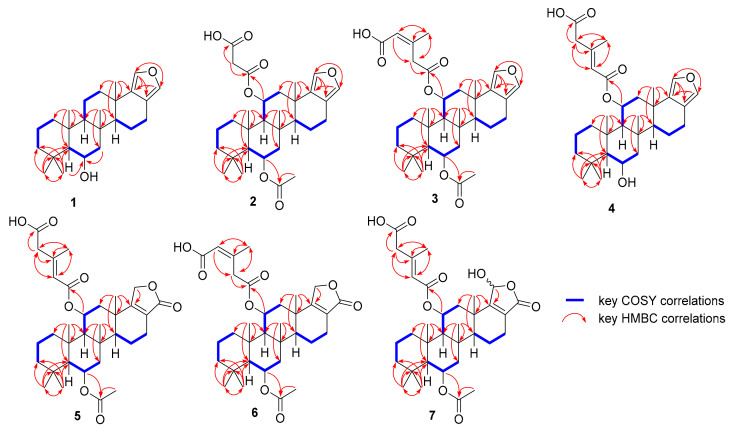
Key 2D NMR (COSY and HMBC) correlations of sesterterpenoids **1**–**7**.

**Figure 3:**
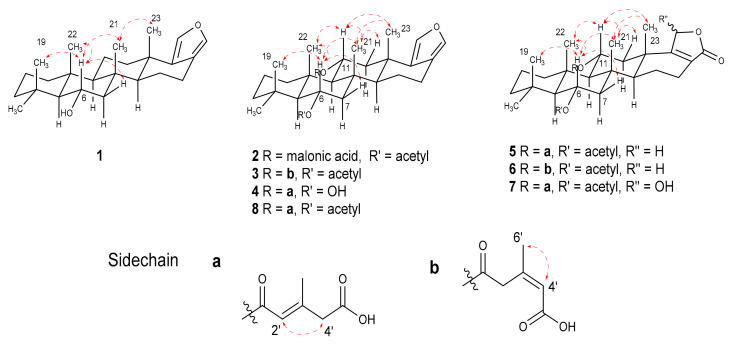
Relevant NOESY correlations (dashed) used to assign the relative stereochemistry of compounds **1**–**8**.

Compound **1**, nambiscalarane B, was isolated as a yellow amorphous powder. Its molecular formula was determined as C_25_H_38_O_2_ on the basis of negative-ion HRESIMS (*m*/*z* 369.27881 [M − H]^−^, calcd 369.2799). Inspection of the ^1^H NMR spectrum of compound **1** (Table 1 and Table 2) showed resonance peaks for five methyl groups attached to quaternary carbons at δ_H_ 0.90 (s, H_3_-22), 0.98 (s, H_3_-21), 1.02 (s, H_3_-19), 1.15 (s, H_3_-20), and 1.19 (s, H_3_-23) ppm; two olefinic protons at δ_H_ 7.04 (d, *J* = 1.2 Hz, H-24) and 7.07 (d, *J* = 1.2 Hz, H-25) ppm; as well as an oxygenated methine group at δ_H_ 4.01 (td, *J* = 11.6, 4.0 Hz, H-6) ppm. The ^13^C, DEPTQ, and HSQC NMR spectroscopic data (Table 2) revealed twenty-five carbon resonance peaks, consisting of five methyl, eight methylene, six methine, and six quaternary carbons. These data suggested the presence of a sesterterpenoid core. COSY and HMBC correlations (Figure 2) established the presence of four spin systems, enabling the assignment of fragments C-1/C-2/C-3, C-5/C-6/C-7, C-9/C-11/C-12, and C-14/C-15/C-16. The key HMBC correlations from H-24 to C-17, C-18, and C-25, as well as H-25 to C-17, C-18, and C-24, indicated the presence of a disubstituted furan ring at these positions. The HMBC correlations of H_3_-19(20) to C-3, C-4, C-5, and C-20(19), of H-5 to C-6, and of H-7 to C-6 led to the location of the hydroxy group at C-6. In addition, correlations from H_3_-22 to C-1, C-5, C-9, and C-10, of H_3_-21 to C-7, C-8, C-9, and C-14, and of H_3_-23 to C-12, C-13, C-14, and C-18 confirmed the presence of a tetracyclic scalarane sesterterpenoid skeleton, which was similar to the known analogue 16-deacetoxy-12-epi-scalarafuranacetate isolated from *Spongia officinalis* in 1989 [20]. However, compound **1** contains a hydroxy group at C-6 instead of an acetyl group at C-12 in the known analogue. The relative configuration of compound **1** was determined by analysis of NOESY correlations and coupling constants (Figure 3). The NOESY correlations between H-6β/H-7β/H_3_-19/H_3_-21/H_3_-22 and between H_3_-21/H_3_-23 indicated that all these groups are located on the same face. Moreover, the large ^1^H-^1^H coupling constant of H-6 and H-11 (11.6 Hz) indicated an axial orientation [21]. Comparison of the optical rotation of compound **1** ([α]^25^_D_ +56.6, c 0.2, CHCl_3_) with 16-deacetoxy-12-*epi*-scalarafurane acetate, an analogous sesterterpenoid ([α]^19^_D_ +70.0, c 0.15, CHCl_3_) [22], suggested that the absolute configuration of compound **1** should be identical to the one of the known analogues. In order to confirm this assertion, the experimental ECD curve of compound **1** was compared with the ECD curve of compound **2**, for which ECD calculations were undertaken (*vide infra*). Thus, the configuration of compound **1** was established as 5*S*, 6*S*, 8*R*, 9*S*, 10*R*, 13*S*, 14*S*.

Compound **2**, nambiscalarane C, was isolated as a pale yellow oil. Its molecular formula was determined as C_30_H_42_O_7_ on the basis of negative-ion HRESIMS (*m*/*z* 513.2846 [M − H]^−^, calcd 513.2858; 469.2953 [M − CO_2_]^−^, calcd 469.2959). The molecular ion peak could be observed but at a relatively low intensity due to rapid fragmentation via decarboxylation of the malonate. The ^1^H and ^13^C NMR data of compound **2** (Table 1 and Table 2) were similar to those of compound **1**.

**Figure 4:**
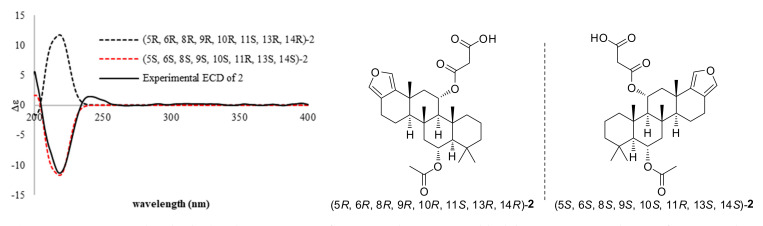
Experimental and calculated ECD spectra of compound **2** in MeOH (black line, experimental ECD of compound **2**; red dashed line, calculated for 5*S*, 6*S*, 8*S*, 9*S*, 10*S*, 11R, 13*S*, 14*S* configurations; black dashed line, calculated for its enantiomer).

The strong deshielding of position 11 (δ_H_/δ_C_ 5.49 (td, *J* = 11.2, 3.0 Hz)/72.3 ppm) suggested the presence of an ester group connected to this position through its oxygen atom. The moderate deshielding of the proton at position 6 (δ_H_/δ_C_ 5.23 (td, *J* = 11.2, 3.3 Hz)/70.6 (C-6) ppm) suggested that the hydroxy group observed in compound 1 was esterified. Extra signals of an acetate (δ_H_/δ_C_ 2.06, s/22.1 (C-2″) and δ_C_ 170.6 (C-1″) ppm) and a malonate (δ_H_/δ_C_ 3.43, s/41.4 (C-2′); δ_C_ 166.6 (C-1′) and 170.0 (C-3′) ppm) were also detected. HMBC correlations (Figure 2) between H-6 to C-1″ and H-11 to C-1′ located the acetate at C-6 and the malonate at C-11. In addition, the NMR data of **2** were very similar those of nambiscalarane (**8**), with slight differences arising from the substitution of the hydroxy group at C-11 by a malonate. NOESY correlations (Figure 3) between H-11β/H_3_-21/H_3_-22/H_3_-23 and H-6β/H-7β/H_3_-19/H_3_-21/H_3_-22, as well as the large ^1^H-^1^H coupling constants between H-6/H-7 (11.2 Hz) and H-11/H-12 (11.2 Hz) indicated that the relative configuration of compound **2** was identical to that of compound **1**. The absolute configuration of compound **2** was established through comparison of calculated and experimental ECD curves. The conformational analysis of compound **2** showed one lowest energy conformer (Figure S2). The ECD spectra of the two possible enantiomers (5*S*, 6*S*, 8*S*, 9*S*, 10*S*, 11*R*, 13*S*, 14*S* and 5*R*, 6*R*, 8*R*, 9*R*, 10*R*, 11*S*, 13*R*, 14*R*) based on the established relative configuration of compound **2** were initially optimized at the B3LYP level, using 6–311G(d,p). TDDFT was then utilized in MeOH to predict the rotational strengths of the transition states using the CAMB3LYP/6–311++G(d,p) level, and these calculated spectra were compared with the experimental ECD spectrum of compound **2**. The calculated ECD spectrum of the (5*S*, 6*S*, 8*S*, 9*S*, 10*S*, 11*R*, 13*S*, 14*S*)-enantiomer was in excellent agreement with the experimental curve (Figure 4). In addition, the optical rotation of compound **2** ([α]^25^_D_ +50.0, c 0.2, CHCl_3_) was compared with the optical rotation of a known analogous scalarane sesterterpenoid ([α]^19^_D_ +70.0, c 0.15, CHCl_3_) [22]. Their similarity further confirmed our assignment of the absolute configuration of compound **2**. 

Compound **3**, nambiscalarane D, was isolated as a pale yellow oil. Its molecular formula was determined as C_33_H_46_O_7_ on the basis of negative-ion HRESIMS (*m*/*z* 553.3176 [M − H]^−^, calcd 553.3171). The ^1^H and ^13^C NMR data of compound **3** (Table 1 and Table 2) were very similar to those of compound **2**, except for the malonyl group at C-11. Analysis of 2D NMR spectra (Figure 2) led to the conclusion that it was replaced by a 3-methylglutaconic acid unit, which was connected through the oxygen at its carbon n° 5 (Figure 1) (δ_H_/δ_C_ 5.90, s/118.8 (C-4′); 3.75 (d, *J* = 16.4 Hz), 3.49 (d, *J* = 16.0 Hz)/39.8 (C-2′); 2.02, s/26.6 (C-6′); δ_C_ 154.0 (C-3′), 169.3 (C-1′), and 170.9 (C-5′) ppm). Analysis of NOESY correlations (Figure 3) and coupling constants showed that compound **3** shared the same relative configuration as compound **2**. The NOESY correlations between H-4′/H_3_-6′ indicated a (*Z*) geometry for the double bond (Figure 3). 

**Figure 5:**
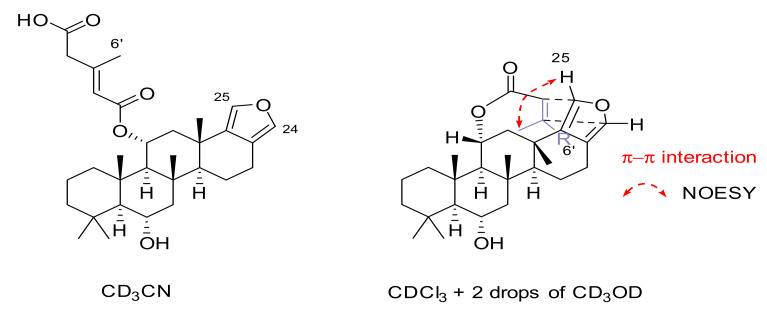
Effect of the NMR solvent on the conformation of compound **4**.

Compound **4**, nambiscalarane E, was isolated as a white viscous oil. Its molecular formula was determined as C_31_H_44_O_6_ on the basis of its negative-ion HRESIMS (*m*/*z* 511.3081 [M − H]^−^, calcd 511.3065). The ^1^H and ^13^C NMR data of **4** (Table 1 and Table 2) were very similar to those of compound **3**. The shielding of H-6 (from δ_H_ 5.23 to 3.91 ppm) and the absence of signals of an acetate moiety in the proton and carbon NMR spectra suggested deacetylation at this position. The shielding of H-4 suggested a change in the nature of the ester group’s tail (at C-11). Analysis of 2D NMR data (Figure 2) indicated that the sidechain of C-11 was replaced by a 3-methylglutaconic acid unit, which was connected through the oxygen at its carbon n° 1 (Figure 1) (δ_H_/δ_C_ 5.69, s/120.1 (C-2′); 3.10, s/45.9 (C-4′); 2.23, s/19.1 (C-6′); 172.6 (C-5′), 165.5 (C-1′), and 152.0 (C-3′) ppm). The NOESY (Figure 3) correlations of H-11β/H-12β/H_3_-21/H_3_-22/H_3_-23 and H-6β/H-7β/H_3_-19/H_3_-21/H_3_-22, as well as the large coupling constants of H-6 (10.8 Hz) and H-11 (11.2 Hz) indicated that the relative configuration of **4** was identical to that of compounds **2** and **3**. The geometry of the double bond was identified as (*E*) due to the NOESY correlation observed between H-2′/H-4′ (Figure 3). Interestingly, a NOESY correlation between H_3_-6′/H-25 (Figure S41) was observed when CDCl_3_ with 2 drops of CD_3_OD was used as a solvent, indicating that an intramolecular π-π interaction was present between C-2′/C-3′ and the furan ring (Figure 5). This interaction was likely responsible for the splitting of the ^13^C NMR resonance peaks at δ_C_ 119.3/119.1 (C-17), 136.6/136.4 (C-18), 137.0/136.9 (C-24), and 135.0/134.9 (C-25) ppm (Table 2 and Figure S36) initially observed. These pairs of resonance peaks merged at δ_C_ 118.3 (C-17), 137.7 (C-18), 138.0 (C-24), and 136.1 (C-25) ppm in deuterated acetonitrile (Table 2 and Figure S43).

Compound **5**, nambiscalarane F, was isolated as a white viscous oil. Its molecular formula was determined as C_33_H_46_O_8_ on the basis of negative-ion HRESIMS (*m*/*z* 569.31275 [M − H]^−^, calcd 569.3120). The NMR data (Table 1 and Table 2) of compound **5** were very similar to those of **4** and the known compound nambiscalarane (**8**). Dramatic differences arose in the furan region. Analysis of 2D NMR data (Figure 2) led to the conclusion that the furan ring was replaced by an α,β-unsaturated butanolide moiety (δ_H_/δ_C_ 4.73 (dt, *J* = 16.8, 2.8 Hz), 4.59 (ddd, *J* = 17.0, 3.8, 1.6 Hz)/68.2 (C-25); 174.1 (C-24), 169.1 (C-18) and 123.9 (C-17) ppm). Analysis of NOESY correlations (Figure 3) and coupling constants showed that compound **5** shared the same relative configuration as compounds **2**–**4**. The geometry of the double bond was determined as (*E*) in a similar manner to compound **4**.

Compound **6**, nambiscalarane G, was isolated as a white viscous oil. Its molecular formula was determined as C_33_H_46_O_8_ on the basis of negative-ion HRESIMS (*m*/*z* 569.3122 [M − H]^−^, calcd 569.3120). The NMR spectroscopic data of **6** (Table 1 and Table 2) were extremely similar to those of compound **5**. Analysis of 2D NMR spectra (Figure 2) led to the conclusion that the ester unit at position 11 was altered at its tail. The sidechain of C-11 was replaced by a 3-methylglutaconic acid unit, which was connected through the oxygen at its carbon n° 5, as in compound 3 (Figure 1) (δ_H_/δ_C_ 5.91, s/118.7 (C-4′); 3.74 (d, *J* = 16.0 Hz), 3.53 (d, *J* = 16.0 Hz)/39.8 (C-2′); 2.02, s/26.7 (C-6′); 169.3 (C-1′), 153.8 (C-3′) and 169.9 (C-5′) ppm). Analysis of NOESY correlations and coupling constants showed that compound **6** shared the same relative configuration as compound **5**. Moreover, the double bond geometry was determined as (*Z*) in a similar manner to compound **3**.

Compound **7**, nambiscalarane H, was isolated as a yellow viscous oil. Its molecular formula was determined as C_33_H_46_O_9_ on the basis of negative-ion HRESIMS (*m*/*z* 585.30774 [M − H]^−^, calcd 585.3069). The ^1^H and ^13^C NMR data of compound **7** (Table 1 and Table 2) were extremely similar to those of compound **5**. Analysis of 2D NMR spectra (Figure 2) led to the conclusion that the butanolide was altered. Position C-25 was substituted by a hydroxy group, leading to a 1:1 mixture of hemiacetal epimers (δ_H_/δ_C_ 5.84:5.82, s/98.8:98.7 ppm). Analysis of NOESY correlations and coupling constants showed that compound **7** shared the same relative configuration and double bond geometry as compound **5**.

Compound **8**, nambiscalarane, was isolated as a yellow viscous oil. Its NMR data were extremely similar to those of compound **4**, except for the absence of an acetate group at position 6. Our spectroscopic data matched with reported values [5]. The relative and absolute configurations of this compound were not described at the time of its discovery. Therefore, NOESY and ECD spectra were recorded. Analysis of NOESY (Figure 3) correlations and coupling constants showed that compound **8** shared the same relative configuration as compounds **2**–**4**. The double bond geometry was determined as (*E*) in a similar manner to compound **4**.

**Figure 6:**
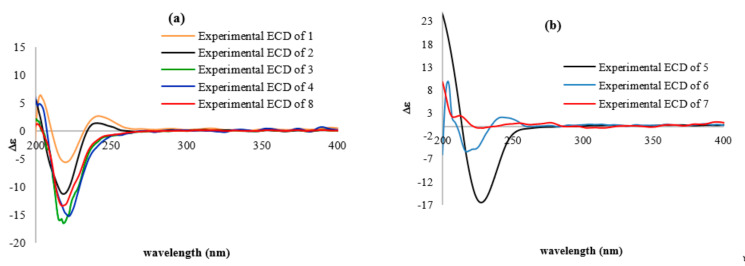
(**a**) Comparison of ECD spectra (black line, experimental ECD of compound **2**; orange line, experimental ECD of compound **1**; green line, experimental ECD of compound **3**; blue line, experimental ECD of compound **4**; red line, experimental ECD of compound **8**). (**b**) Experimental ECD spectra (black line, experimental ECD of compound **5**; blue line, experimental ECD of compound **6**; red line, experimental ECD of compound **7**); all compounds were recorded in MeOH.

The absolute configurations of compounds **3**–**8** were assigned by analogy with compound **2**, as they showed similar ECD curves (Figure 4 and Figure 6a,b) and optical rotations of identical sign, although the ECD curve of compound **7** proved inconclusive (Figure 6b), probably due to the presence of two epimers in a 1:1 ratio.

### 3.3. Cytotoxic Activities of Scalarane Sesterterpenoids ***1**–**8***

Compounds **1**–**8** were assessed for cytotoxic activity against A549 human lung carcinoma cell line, HT29 human colon cancer, HeLa human cervical cancer, and HCT-116 human colon cancer, cancer cell lines by the MTT assay (Table 3). *Cis*-platin was used as the positive control. Compound **7** was inactive against all the tested cell lines. Compounds **1**, **3**, **4**, and **8** showed moderate cytotoxicity to A549 cell line, with IC_50_ values ranging from 22.95 to 27.51 µM, while compounds **2**, **5**, and **6** were inactive. Compounds **1**–**4** and **8** showed moderate activities against HT29 cell line with IC_50_ values ranging from 20.39 to 33.02 µM, while compound **5** showed weak activity (IC_50_ 54.46 µM) and compound **6** was inactive. Compounds **3**, **4**, and **8** showed moderate activities against HeLa cell line with IC_50_ values ranging from 21.14 to 26.55 µM, compounds **1** and **6** showed weak activities (IC_50_ 40.81 and 45.94 µM, respectively), while compounds **2** and **5** were inactive. Compounds **2**, **4**, and **6** showed strong cytotoxicity against HCT-116 cell line with IC_50_ values ranging from 13.41 to 16.53 µM, while compounds **1**, **3**, **5**, and **8** showed moderate activities with IC_50_ values ranging from 20.28 to 32.70 µM. No clear conclusion could be drawn in term of structure–activity relationship among the eight sesterterpenoids tested. In addition, the tested compounds proved inactive towards *Vero* cells.

### 3.4. Antibacterial Activities of Scalarane Sesterterpenoids ***1**–**8***

The isolated scalarane sesterterpenoids were evaluated for their antibacterial activities against four Gram-positive and one Gram-negative bacteria (Table 4). Compounds **5** and **8** showed moderate activities against *Staphylococcus aureus* with MIC values of 16 μg/mL. Compound **8** also showed strong antibacterial activity against *Bacillus cereus* with a MIC value of 16 μg/mL, which is equivalent to that of kanamycin. Compound **3** showed moderate antibacterial activity against *Bacillus subtilis* with a MIC value of 16 μg/mL. Compound **8** showed strong antibacterial activity against *Bacillus subtilis* with a MIC value of 8 μg/mL, which is equivalent to that of kanamycin. The other compounds showed either weak (MIC values in the range of 32−128 μg/mL) or no antibacterial activity against the tested bacterial strains.

## 4. Conclusions

Nambiscalaranes B–H (**1**–**7**), nambiscalarane (**8**), and aurisin A (**9**) were isolated from the cultured mycelium of the luminescent mushroom *Neonothopanus nambi*. The structures of compounds **1**–**9** were determined by thorough analysis of spectroscopic data (mostly NMR spectra) and ECD data. Compounds **1**–**8** were tested for anti-proliferative activities against A549, HT29, HeLa, and HCT-116 cancer cell lines. Remarkably, compounds **3**, **5**, and **7** showed strong cytotoxicity against HCT-116 cell line, with IC_50_ values ranging from 13.41 to 16.53 µM, and showed no cytotoxicity towards *Vero* cells. In addition, compounds **1**–**8** were tested for antibacterial activities against selected Gram-positive and Gram-negative bacteria, with diverse results. Interestingly, compound **8** inhibited the growth of *Bacillus subtilis* with a MIC value of 8 µg/mL, which was equivalent to the MIC value of the standard kanamycin.

## Figures and Tables

**Table 1:** ^1^H NMR (400 MHz) data for compounds **1**–**7** (δ in ppm and J in Hz).

No.	1 ^a^	2 ^a^	3 ^a^	4 ^b^	5 ^a^	6 ^a^	7 ^c^
1	1.97 (m) 1.67 (m)	1.84 (dt, 12.8, 5.2) 1.27 (overlap)	1.93 (m) 1.21 (overlap)	1.81 (m) 1.20 (m)	1.91 (m) 1.28 (overlap)	1.90 (m) 1.22 (overlap)	1.96 (m) 1.38 (m)
2	1.60 (m) 1.42 (m)	1.53 (m) 1.39 (m)	1.51 (m) 1.37 (overlap)	1.45 (m) 1.32 (overlap)	1.53 (m) 1.40 (m)	1.54 (m) 1.38 (m)	1.58 (m) 1.44 (m)
3	1.35 (m) 1.20 (m)	1.37 (overlap) 1.20 (overlap)	1.37 (overlap) 1.18 (overlap)	1.31 (overlap) 1.11 (overlap)	1.37 (m) 1.19 (m)	1.37 (m) 1.22 (overlap)	1.38 (m) 1.23 (m)
4	-	-	-	-	-	-	-
5	0.89 (m)	1.27 (overlap)	1.29 (overlap)	0.94 (d, 10.8)	1.29 (overlap)	1.30 (m)	1.39 (d, 11.6)
6	4.01 (td, 11.6, 4.0)	5.23 (td, 11.2, 3.3)	5.23 (td, 11.0, 3.2)	3.91 (td, 10.8, 3.5)	5.23 (td, 10.8, 3.5)	5.22 (td, 11.2, 3.5)	5.26 (td, 11.2, 3.7)
7*β*	2.18 (dd 11.6, 3.6)	2.13 (dd, 11.6, 3.2)	2.11 (dd, 12.0, 3.6)	2.11 (dd, 12.0, 3.6)	2.10 (dd, 12.0, 3.6)	2.08 (overlap)	2.07 (dd, 11.8, 3.4)
7*α*	1.00 (m)	1.07 (overlap)	1.06 (overlap)	1.02 (overlap)	1.09 (m)	1.08 (overlap)	1.19 (m)
8	-	-	-	-	-	-	-
9	0.89 (m)	1.40 (d, 11.2)	1.38 (d, 11.2)	1.31 (d, 11.2)	1.38 (d, 10.8)	1.36 (m)	1.46 (d, 11.2)
10	-	-	-	-	-	-	-
11	1.60 (m) 1.42 (m)	5.49 (td, 11.2, 3.0)	5.42 (td, 11.2, 2.8)	5.40 (td, 10.8, 3.3)	5.47 (td, 10.8, 3.6)	5.42 (td, 11.0, 3.5)	5.51 (td, 11.0, 3.6)
12*β*	2.09 (m)	2.33 (dd, 12.4, 3.0)	2.30 (dd, 12.0, 3.6)	2.24 (dd, 11.6, 3.2)	2.01 (m)	1.98 (dd, 11.6, 3.6)	2.65 (m)
12*α*	1.55 (m)	1.75 (t, 11.2)	1.69 (m)	1.66 (t, 11.4)	1.52 (m)	1.52 (m)	1.48 (m)
13	-	-	-	-	-	-	-
14	1.22 (m)	1.26 (dd, 11.6, 5.6)	1.28 (overlap)	1.22 (d, 12.0)	1.25 (overlap)	1.25 (overlap)	1.32 (m)
15	1.77 (m) 1.60 (m)	1.75 (m) 1.60 (m)	1.70 (m) 1.59 (m)	1.81 (m) 1.56 (dd, 12.4, 6.4)	1.88 (m) 1.53 (m)	1.87 (m) 1.50 (m)	1.94 (m) 1.86 (dd, 13.2, 6.4)
16	2.77 (dd, 16.4, 6.4) 2.43 (dddd, 16.4, 12.4, 7.2, 1.6)	2.76 (dd, 16.4, 5.6) 2.43 (m)	2.76 (dd, 16.6, 6.2) 2.42 (m)	2.72 (dd, 16.4, 6.0) 2.39 (m)	2.42 (dd, 17.4, 5.0) 2.13 (m)	2.41 (dd, 17.6, 4.8) 1.92 (m)	2.53 (d, 5.2)/2.48 (d, 5.2) 2.34 (m)
17	-	-	-	-	-	-	-
18	-	-	-	-	-	-	-
19	1.02 (s)	0.87 (s)	0.87 (s)	0.98 (s)	0.87 (s)	0.87 (s)	0.88 (s)
20	1.15 (s)	1.05 (s)	1.05 (s)	1.10 (s)	1.06 (s)	1.06 (s)	1.09 (s)
21	0.98 (s)	1.15 (s)	1.14 (s)	1.04 (s)	1.18 (s)	1.15 (s)	1.17 (s)
22	0.90 (s)	1.09 (s)	1.07 (s)	0.96 (s)	1.08 (s)	1.07 (s)	1.09 (s)
23	1.19 (s)	1.31 (s)	1.29 (s)	1.27 (s)	1.29 (s)	1.25 (s)	1.29 (s)
24	7.04 (d, 1.2)	7.04 (s)	7.02 (d, 1.6)	6.98 (s)	-	-	-
25	7.07 (d, 1.2)	7.07 (s)	7.06 (d, 1.2)	7.00 (s)	4.73 (dt, 16.8, 2.8) 4.59 (ddd, 17.0, 3.8, 1.6)	4.72 (d, 17.2) 4.62 (d, 16.8)	5.84 (s) 5.82 (s)
1′		-	-	-	-	-	-
2′		3.43 (s)	3.75 (d, 16.4) 3.49 (d, 16.0)	5.69 (s)	5.73 (d, 1.2)	3.74 (d, 16.0) 3.53 (d, 16.0)	5.75 (s)
3′		-	-	-	-	-	-
4′		-	5.90 (s)	3.10 (s)	3.21 (s)	5.91 (s)	3.19 (s)
5′		-	2.02 (s)	-	-	2.02 (s)	-
6′		-	-	2.23 (s)	2.30 (d, 1.2)	-	2.26 (d, 0.8)
1″		-	-	-	-	-	-
2″		2.06 (s)	2.05 (s)	-	2.07 (s)	2.06 (s)	2.05 (s)

^a^ Recorded in CDCl_3_. ^b^ Recorded in CDCl_3_ + 2 drops of CD_3_OD. ^c^ Recorded in CD_3_OD.

**Table 2:** ^13^C NMR spectroscopic data (100 MHz) for compounds **1**–**7** (δ in ppm).

No.	1 ^a^	2 ^a^	3 ^a^	4 ^b^	4 ^c^	5 ^a^	6 ^a^	7 ^d^
1	40.1	41.7	41.5	41.4	42.2	41.4	41.4	42.5
2	18.1	18.8	18.8	18.9	19.1	18.8	18.7	19.6
3	43.7	41.9	41.9	42.0	42.6	42.1	41.9	43.0
4	33.9	33.5	33.5	33.7	34.3	33.5	33.5	34.3
5	61.9	56.8	57.0	59.4	59.5	56.8	56.9	57.8
6	68.7	70.6	70.7	67.6	67.2	70.4	70.3	71.8
7	53.7	48.4	48.5	52.7	53.4	48.2	48.2	49.1
8	39.1	40.9	40.8	40.7	41.4	40.6	40.6	41.5
9	60.9	62.9	63.0	62.9	63.6	63.0	62.8	64.0/63.9
10	39.7	41.4	41.9	41.7	42.4	41.8	41.8	42.9
11	18.3	72.3	70.9	69.3	69.9	68.5	70.1	69.6
12	41.3	47.9	47.9	48.3	49.1	44.8	44.3	45.4/44.7
13	34.3	35.1	35.1	34.9	35.8	37.5	37.5	36.7/36.6
14	56.9	56.2	56.3	56.2	56.9	55.7	55.6	57.2
15	18.7	18.2	18.2	18.0	18.7	17.2	17.2	18.1/17.9
16	20.9	20.5	20.6	20.4	21.0	21.6	21.6	25.1/24.8
17	119.9	119.3	119.4	119.3, 119.1	118.3	123.9	123.8	137.9/137.8
18	137.6	136.3	136.5	136.6, 136.4	137.7	169.1	169.1	161.3/161.0
19	22.2	23.1	23.1	23.0	23.4	23.1	23.0	23.3
20	36.7	35.7	35.8	36.1	36.3	35.8	35.8	36.3
21	19.1	19.6	19.6	19.8	20.2	19.8	19.8	20.1
22	17.9	19.4	19.2	19.4	20.0	19.3	19.2	19.7
23	26.4	27.0	27.0	26.9	27.2	22.9	22.8	22.7
24	136.9	137.2	137.0	137.0, 136.9	138.0	174.1	174.2	171.8/171.4
25	135.2	135.1	135.2	135.0, 134.9	136.1	68.2	68.2	98.8/98.7
1′	-	166.6	169.3	165.5	166.0	165.1	169.3	166.8
2′	-	41.4	39.8	120.1	120.6	120.2	39.8	120.4
3′	-	170.0	154.0	152.0	153.1	151.9	153.8	154.6
4′	-	-	118.8	45.9	45.8	45.3	118.7	46.6
5′	-	-	170.9	172.6	171.4	173.0	169.9	173.8
6′	-	-	26.6	19.1	19.6	19.3	26.7	19.3
1″	-	170.6	170.5	-	-	170.5	170.5	172.2
2″	-	22.1	22.2	-	-	22.2	22.2	22.0

^a^ Recorded in CDCl_3_. ^b^ Recorded in CDCl_3_ + 2 drops of CD_3_OD. ^c^ Recorded in CD_3_CN. ^d^ Recorded in CD_3_OD.

**Table 3:** Anti-proliferative activities of compounds **1**–**8** (IC_50_ µM).

Compound	A549	HT29	HeLa	HCT-116	*Vero* Cells
**1**	24.28 ± 7.56	22.71 ± 0.16	40.81 ± 0.73	20.28 ± 3.13	>100
**2**	85.03 ± 10.17	33.02 ± 1.63	73.23 ± 5.37	14.33 ± 0.56	>100
**3**	24.99 ± 1.59	20.75 ± 0.63	21.14 ± 1.66	21.41 ± 1.14	70.21 ± 8.23
**4**	22.95 ± 1.99	19.62 ± 2.32	23.72 ± 0.51	13.41 ± 0.39	>100
**5**	99.14 ± 0.36	54.46 ± 5.93	72.92 ± 5.23	32.70 ± 2.37	>100
**6**	>100	78.25 ± 4.28	45.94 ± 2.77	16.53 ± 2.79	>100
**8**	27.51 ± 0.32	20.39 ± 2.07	26.55 ± 2.58	24.41 ± 2.06	>100
*cis*-platin	16.86 ± 0.03	5.30 ± 0.23	6.43 ± 0.13	4.93 ± 0.77	>100

Mean ± S.D. (*n* = 3); Compound **7** was inactive (IC_50_ > 100 µM).

**Table 4:** Antibacterial activities of compounds **1**–**8** (MIC μg/mL).

Compound ^1,2^	*Staphylococcus aureus* ATCC 25923	*Bacillus cereus* ATCC 11778	*Bacillus subtilis* ATCC 6633	Methicillin Resistant *Staphylococcus aureus* DMST 20654
**1**	128	inactive	inactive	inactive
**2**	128	32	32	128
**3**	inactive	32	16	inactive
**4**	128	inactive	inactive	inactive
**5**	16	128	128	inactive
**6**	64	128	inactive	inactive
**7**	inactive	64	64	inactive
**8**	16	16	8	128
Kanamycin	2	16	8	4
Chloramphenicol	8	4	4	8

^1^ All compounds were inactive with Gram-negative bacteria, *Shigella sonnei* ATCC 11060. ^2^ Inactive at >128 μg/mL.

## Supplementary Materials

The following are available online. Table S1: Secondary metabolites from *Neonothopanus nambi*, Figure S1: Chemical constituents from *Neonothopanus nambi*, Figure S2: Experimental and calculated ECD spectra of **2** (black line, experimental ECD of **2**; red dashed line, calculated for 5*S*, 6*S*, 8*S*, 9*S*, 10*S*, 11*R*, 13*S*, 14*S* configurations; black dashed line, calculated for its enantiomer). Figure S3: Structures of conformers of **2** computed by HyperChem, Table S2: Conformer Boltzmann distribution of **2** computed by HyperChem, Figure S4: IR spectrum of compound **1**, Figure S5: UV spectrum of **1** in methanol, Figure S6: HRESIMS spectrogram of compound **1**, Figure S7: ^1^H NMR spectrum of compound **1** in CDCl_3_, Figure S8: ^13^C NMR spectrum of compound **1** in CDCl_3_, Figure S9: DEPTQ spectrum of compound **1** in CDCl_3_, Figure S10: ^1^H-^1^H COSY NMR spectrum of compound **1** in CDCl_3_, Figure S11: HSQC NMR spectrum of compound **1** in CDCl_3_, Figure S12: HMBC NMR spectrum of compound **1** in CDCl_3_, Figure S13: ^1^H-^1^H NOESY NMR spectrum of compound **1** in CDCl_3_ (overview), Figure S14: ^1^H-^1^H NOESY NMR spectrum of compound **1** in CDCl_3_ (expanded view of the 0.0–4.5 ppm region), Figure S15: IR spectrum of compound **2**, Figure S16: UV spectrum of **2** in methanol, Figure S17: HRESIMS spectrogram of compound **2**, Figure S18: ^1^H NMR spectrum of compound **2** in CDCl_3_, Figure S19: ^13^C NMR spectrum of compound **2** in CDCl_3_, Figure S20: DEPT spectrum of compound **2** in CDCl_3_, Figure S21: ^1^H-^1^H COSY NMR spectrum of compound **2** in CDCl_3_, Figure S22: HSQC spectrum of compound **2** in CDCl_3_, Figure S23: HMBC spectrum of compound **2** in CDCl_3_, Figure S24: ^1^H-^1^H NOESY NMR spectrum of compound **2** in CDCl_3_ (overview), Figure S25: ^1^H-^1^H NOESY NMR spectrum of compound **2** in CDCl_3_ (expanded view of the 0.0–6.0 ppm region), Figure S26: ^1^H-^1^H NOESY NMR spectrum of compound **2** in CDCl_3_ (expanded view of the 0.0–3.0 ppm region), Figure S27: IR spectrum of compound **3**, Figure S28: UV spectrum of compound **3** in methanol, Figure S29: HRESIMS spectrogram of compound **3**, Figure S30: ^1^H NMR spectrum of compound **3** in CDCl_3_, Figure S31: ^13^C NMR spectrum of compound **3** in CDCl_3_, Figure S32: DEPT spectrum of compound **3** in CDCl_3_, Figure S33: ^1^H-^1^H COSY NMR spectrum of compound **3** in CDCl_3_, Figure S34: HSQC spectrum of compound **3** in CDCl_3_, Figure S35: HMBC spectrum of compound **3** in CDCl_3_, Figure S36: ^1^H-^1^H NOESY NMR spectrum of compound **3** in CDCl_3_ (overview), Figure S37: ^1^H-^1^H NOESY NMR spectrum of compound **3** in CDCl_3_ (expanded view of the 0.1–2.9 × 5.05–5.55 ppm region), Figure S38: ^1^H-^1^H NOESY NMR spectrum of compound **3** in CDCl_3_ (expanded view of the 0.0–3.0 ppm region), Figure S39: IR spectrum of compound **4**, Figure S40: UV spectrum of 4 in methanol, Figure S41: HRESIMS spectrogram of compound **4**, Figure S42: ^1^H NMR spectrum of compound **4** in CDCl_3_ + 2 drops of CD_3_OD, Figure S43: ^13^C NMR spectrum of compound **4** in CDCl_3_ + 2 drops of CD_3_OD, Figure S44: DEPTQ spectrum of compound **4** in CDCl_3_ + 2 drops of CD_3_OD, Figure S45: ^1^H-^1^H COSY spectrum of compound **4** in CDCl_3_ + 2 drops of CD_3_OD, Figure S46: HSQC spectrum of compound **4** in CDCl_3_ + 2 drops of CD_3_OD, Figure S47: HMBC spectrum of compound **4** in CDCl_3_ + 2 drops of CD_3_OD, Figure S48: ^1^H-^1^H NOESY spectrum of compound **4** in CDCl_3_ + 2 drops of CD_3_OD (overview), Figure S49: ^1^H-^1^H NOESY NMR spectrum of compound **4** in CDCl_3_ + 2 drops of CD_3_OD (expanded view of the 0.0–6.0 ppm region), Figure S50: ^1^H-^1^H NOESY NMR spectrum of compound **4** in CDCl_3_ + 2 drops of CD_3_OD (expanded view of the 0.0–3.0 ppm region), Figure S51: ^1^H NMR spectrum of compound **4** in CD_3_CN, Figure S52: ^13^C NMR spectrum of compound **4** in CD_3_CN, Figure S53: IR spectrum of compound **5**, Figure S54: UV spectrum of **5** in methanol, Figure S55: HRESIMS spectrogram of compound **5**, Figure S56: ^1^H NMR spectrum of compound **5** in CDCl_3_, Figure S57: _13_C NMR spectrum of compound **5** in CDCl_3_, Figure S58: DEPTQ spectrum of compound **5** in CDCl_3_, Figure S59: ^1^H-^1^H COSY NMR spectrum of compound **5** in CDCl_3_, Figure S60: HSQC spectrum of compound **5** in CDCl_3_, Figure S61: HMBC spectrum of compound **5** in CDCl_3_, Figure S62: ^1^H-^1^H NOESY spectrum of compound **5** in CDCl_3_ (overview), Figure S63: ^1^H-^1^H NOESY NMR spectrum of compound **5** in CDCl_3_ (expanded view of the 4.3–5.7 × 0.6–2.5 ppm region), Figure S64: ^1^H-^1^H NOESY NMR spectrum of compound **5** in CDCl_3_ (expanded view of the 0.0–3.0 ppm region), Figure S65: IR spectrum of compound **6**, Figure S66: UV spectrum of **6** in methanol, Figure S67: HRESIMS spectrogram of compound **6**, Figure S68: ^1^H NMR spectrum of compound **6** in CDCl_3_, Figure S69: ^13^C NMR spectrum of compound **6** in CDCl_3_, Figure S70: DEPTQ spectrum of compound **6** in CDCl_3_, Figure S71: ^1^H-^1^H COSY NMR spectrum of compound **6** in CDCl_3_, Figure S72: HSQC spectrum of compound **6** in CDCl_3_, Figure S73: HMBC spectrum of compound **6** in CDCl_3_, Figure S74: ^1^H-^1^H NOESY spectrum of compound **6** in CDCl_3_ (overview), Figure S75: ^1^H-^1^H NOESY NMR spectrum of compound **6** in CDCl_3_ (expanded view of the 0.0–6.0 ppm region), Figure S76: ^1^H-^1^H NOESY NMR spectrum of compound **6** in CDCl_3_ (expanded view of the 0.7–2.6 ppm region), Figure S77: IR spectrum of compound **7**, Figure S78: UV spectrum of **7** in methanol, Figure S79: HRESIMS spectrogram of compound **7**, Figure S80: ^1^H NMR spectrum of compound **7** in CD_3_OD, Figure S81: ^13^C NMR spectrum of compound **7** in CD_3_OD, Figure S82: DEPTQ spectrum of compound **7** in CD_3_OD, Figure S83: ^1^H-^1^H COSY NMR spectrum of compound **7** in CD_3_OD, Figure S84: HSQC spectrum of compound **7** in CD_3_OD, Figure S85: HMBC spectrum of compound **7** in CD_3_OD, Figure S86: ^1^H-^1^H NOESY spectrum of compound **7** in CD_3_OD (overview), Figure S87: ^1^H-^1^H NOESY NMR spectrum of compound **7** in CD_3_OD (expanded view of the 0.0–3.0 ppm region), Figure S88: UV spectrum of **8** in methanol, Figure S89: HRESIMS spectrogram of compound **8**, Figure S90: ^1^H NMR spectrum of compound **8** in CDCl_3_, Figure S91: ^13^C NMR spectrum of compound **8** in CDCl_3_, Figure S92: ^1^H-^1^H NOESY spectrum of compound **8** in CDCl_3_ (overview), Figure S93: ^1^H-^1^H NOESY NMR spectrum of compound **8** in CDCl_3_ (expanded view of the 0.0–6.0 ppm region), Figure S94: ^1^H-^1^H NOESY NMR spectrum of compound **8** in CDCl_3_ (expanded view of the 0.0–3.5 ppm region).
