# Supplementary material for: Scalarane Sesterterpenoids with Antibacterial and Anti-Proliferative Activities from the Mushroom Neonothopanus nambi
Source: Molecules. 2021 Dec 17;26(24):7667. doi: 10.3390/molecules26247667 (PMC8708503; doi:10.3390/molecules26247667)

Supplementary Information

Scalarane Sesterterpenoids with Antibacterial and Anti-Proliferative Activities from the Mushroom *Neonothopanus nambi*

Awat Wisetsai [1], Ratsami Lekphrom [1,2], Sureeporn Bua-art [3], Thanapat Suebrasri [4], Sophon Boonlue [4], Sarawut Tontapha [5], Vittaya Amornkitbamrung [5], Thanaset Senawong [6], Florian T. Schevenels [1,*]

[1] Department of Chemistry, and Center for Innovation in Chemistry, Faculty of Science, Khon Kaen University, Khon Kaen 40002, Thailand; w.awat@kkumail.com (A.W.); ratsami@kku.ac.th (R.L.)
[2] Applied Taxonomic Research Center, Department of Chemistry, and Center for Innovation in Chemistry, Faculty of Science, Khon Kaen University, Khon Kaen 40002, Thailand; ratsami@kku.ac.th
[3] Department of Agriculture, Plant Pathology Research Group Plant Protection Research and Development Office, Bangkok, Thailand; suree.suli@gmail.com
[4] Department of Microbiology, Faculty of Science, Khon Kaen University, Khon Kaen, 40002, Thailand; s.thanapat@kkumail.com (T.S.); bsopho@kku.ac.th (S.B.)
[5] Integrated Nanotechnology Research Center, Department of Physics, Faculty of Science, Khon Kaen University, Khon Kaen 40002, Thailand; s.thanapat@kkumail.com (T.S.); vittaya@kku.ac.th (V.A.)
[6] Department of Biochemistry, Faculty of Science, Khon Kaen University, Khon Kaen 40002, Thailand; sthanaset@kku.ac.th
* Correspondence: florian@kku.ac.th

Reported structures of *N. nambi* in different culture conditions.

Table S1: Secondary metabolites from *Neonothopanus nambi.*

Figure S1: Chemical constituents from *Neonothopanus nambi.*

Fungus material

Conformational Analysis and ECD Calculations of 2 and Their Enantiomers.

Figure S2: Experimental and calculated ECD spectra of **2** (black line, experimental ECD of **2**; red dashed line, calculated for *5S*, *6S*, *8S*, *9S*, *10S*, *11R*, *13S*, *14S* configurations; black dashed line, calculated for its enantiomer).

Figure S3: Structures of conformers of **2** computed by HyperChem.

Table S2: Conformer Boltzmann distribution of **2** computed by HyperChem.

References

Figure S4: IR spectrum of compound **1**.

Figure S5: UV spectrum of **1** in methanol.

Figure S6: HRESIMS spectrogram of compound **1**.

Figure S7: ^{1}H NMR spectrum of compound **1** in $CDCl_3$.

Figure S8: ^{13}C NMR spectrum of compound **1** in $CDCl_3$.

Figure S9: DEPTQ spectrum of compound **1** in $CDCl_3$.

Figure S10: ^{1}H–^{1}H COSY NMR spectrum of compound **1** in $CDCl_3$.

Figure S11: HSQC NMR spectrum of compound **1** in $CDCl_3$.

Figure S12: HMBC NMR spectrum of compound **1** in $CDCl_3$.

Figure S13: ^{1}H–^{1}H NOESY NMR spectrum of compound **1** in $CDCl_3$ (overview).

Figure S14: ^{1}H–^{1}H NOESY NMR spectrum of compound **1** in $CDCl_3$ (expanded view of the 0.0–4.5 ppm region).

Figure S15: IR spectrum of compound **2**.

Figure S16: UV spectrum of **2** in methanol.

Figure S17: HRESIMS spectrogram of compound **2**.

Figure S18: ^{1}H NMR spectrum of compound **2** in $CDCl_3$.

Figure S19: ^{13}C NMR spectrum of compound **2** in $CDCl_3$.

Figure S20: DEPT spectrum of compound **2** in $CDCl_3$.

Figure S21: ^{1}H–^{1}H COSY NMR spectrum of compound **2** in $CDCl_3$.

Figure S22: HSQC spectrum of compound **2** in $CDCl_3$.

Figure S23: HMBC spectrum of compound **2** in $CDCl_3$.

Figure S24: ^{1}H–^{1}H NOESY NMR spectrum of compound **2** in $CDCl_3$ (overview).

Figure S25: ^{1}H–^{1}H NOESY NMR spectrum of compound **2** in $CDCl_3$ (expanded view of the 0.0–6.0 ppm region).

Figure S26: ^{1}H–^{1}H NOESY NMR spectrum of compound **2** in $CDCl_3$ (expanded view of the 0.0–3.0 ppm region).

Figure S27: IR spectrum of compound **3**.

Figure S28: UV spectrum of compound **3** in methanol.

Figure S29: HRESIMS spectrogram of compound **3**.

Figure S3: ^{1}H NMR spectrum of compound **3** in $CDCl_3$.

Figure S31: ^{13}C NMR spectrum of compound **3** in $CDCl_3$.

Figure S32: DEPT spectrum of compound **3** in CDCl3.

Figure S33: ^{1}H–^{1}H COSY NMR spectrum of compound **3** in $CDCl_3$.

Figure S34: HSQC spectrum of compound **3** in $CDCl_3$.

Figure S35: HMBC spectrum of compound **3** in $CDCl_3$.
Figure S36: ^{1}H–^{1}H NOESY NMR spectrum of compound **3** in $CDCl_3$ (overview).
Figure S37: ^{1}H–^{1}H NOESY NMR spectrum of compound **3** in $CDCl_3$ (expanded view of the 0.1–2.9 × 5.05–5.55 ppm region).
Figure S38: ^{1}H–^{1}H NOESY NMR spectrum of compound **3** in $CDCl_3$ (expanded view of the 0.0–3.0 ppm region).
Figure S39: IR spectrum of compound **4**.
Figure S40: UV spectrum of **4** in methanol.
Figure S41: HRESIMS spectrogram of compound **4**.
Figure S42: ^{1}H NMR spectrum of compound **4** in $CDCl_3$ + 2 drops of CD_3OD.
Figure S43: ^{13}C NMR spectrum of compound **4** in $CDCl_3$ + 2 drops of CD_3OD.
Figure S44: DEPTQ spectrum of compound **4** in $CDCl_3$ + 2 drops of CD_3OD.
Figure S45: ^{1}H–^{1}H COSY spectrum of compound **4** in $CDCl_3$ + 2 drops of CD_3OD.
Figure S46: HSQC spectrum of compound **4** in $CDCl_3$ + 2 drops of CD_3OD.
Figure S47: HMBC spectrum of compound **4** in $CDCl_3$ + 2 drops of CD_3OD.
Figure S48: ^{1}H–^{1}H NOESY spectrum of compound **4** in $CDCl_3$ + 2 drops of CD_3OD (overview).
Figure S49: ^{1}H–^{1}H NOESY NMR spectrum of compound **4** in $CDCl_3$ + 2 drops of CD_3OD (expanded view of the 0.0–6.0 ppm region).
Figure S50: ^{1}H–^{1}H NOESY NMR spectrum of compound **4** in $CDCl_3$ + 2 drops of CD_3OD (expanded view of the 0.0–3.0 ppm region).
Figure S51: ^{1}H NMR spectrum of compound **4** in CD_3CN.
Figure S52: ^{13}C NMR spectrum of compound **4** in CD_3CN.
Figure S53: IR spectrum of compound **5**.
Figure S54: UV spectrum of **5** in methanol.
Figure S55: HRESIMS spectrogram of compound **5**.
Figure S56: ^{1}H NMR spectrum of compound **5** in $CDCl_3$.
Figure S57: ^{13}C NMR spectrum of compound **5** in $CDCl_3$.
Figure S58: DEPTQ spectrum of compound **5** in $CDCl_3$.
Figure S59: ^{1}H–^{1}H COSY NMR spectrum of compound **5** in $CDCl_3$.
Figure S60: HSQC spectrum of compound **5** in $CDCl_3$.
Figure S61: HMBC spectrum of compound **5** in $CDCl_3$.
Figure S62: ^{1}H–^{1}H NOESY spectrum of compound **5** in $CDCl_3$ (overview).
Figure S63: ^{1}H–^{1}H NOESY NMR spectrum of compound **5** in $CDCl_3$ (expanded view of the 4.3–5.7 × 0.6–2.5 ppm region).
Figure S64: ^{1}H–^{1}H NOESY NMR spectrum of compound **5** in $CDCl_3$ (expanded view of the 0.0–3.0 ppm region).
Figure S65: IR spectrum of compound **6**.
Figure S66: UV spectrum of **6** in methanol.
Figure S67: HRESIMS spectrogram of compound **6**.
Figure S68: ^{1}H NMR spectrum of compound **6** in $CDCl_3$.
Figure S69: ^{13}C NMR spectrum of compound **6** in $CDCl_3$.
Figure S70: DEPTQ spectrum of compound **6** in $CDCl_3$.
Figure S71: ^{1}H–^{1}H COSY NMR spectrum of compound **6** in $CDCl_3$.
Figure S72: HSQC spectrum of compound **6** in $CDCl_3$.
Figure S73: HMBC spectrum of compound **6** in $CDCl_3$.
Figure S74: ^{1}H–^{1}H NOESY spectrum of compound **6** in $CDCl_3$ (overview).

Figure S75: ^{1}H–^{1}H NOESY NMR spectrum of compound **6** in $CDCl_3$ (expanded view of the 0.0–6.0 ppm region).

Figure S76: ^{1}H–^{1}H NOESY NMR spectrum of compound **6** in $CDCl_3$ (expanded view of the 0.7–2.6 ppm region).

Figure S77: IR spectrum of compound **7**.

Figure S78: UV spectrum of **7** in methanol.

Figure S79: HRESIMS spectrogram of compound **7**.

Figure S80: ^{1}H NMR spectrum of compound **7** in CD_3OD.

Figure S81: ^{13}C NMR spectrum of compound **7** in CD_3OD.

Figure S82: DEPTQ spectrum of compound **7** in CD_3OD.

Figure S83: ^{1}H–^{1}H COSY NMR spectrum of compound **7** in CD_3OD.

Figure S84: HSQC spectrum of compound **7** in CD_3OD.

Figure S85: HMBC spectrum of compound **7** in CD_3OD.

Figure S86: ^{1}H–^{1}H NOESY spectrum of compound **7** in CD_3OD (overview).

Figure S87: ^{1}H–^{1}H NOESY NMR spectrum of compound **7** in CD_3OD (expanded view of the 0.0–3.0 ppm region).

Figure S88: UV spectrum of **8** in methanol.

Figure S89: HRESIMS spectrogram of compound **8**.

Figure S90: ^{1}H NMR spectrum of compound **8** in $CDCl_3$.

Figure S91: ^{13}C NMR spectrum of compound **8** in $CDCl_3$.

Figure S92: ^{1}H–^{1}H NOESY spectrum of compound **8** in $CDCl_3$ (overview).

Figure S93: ^{1}H–^{1}H NOESY NMR spectrum of compound **8** in $CDCl_3$ (expanded view of the 0.0–6.0 ppm region).

Figure S94: ^{1}H–^{1}H NOESY NMR spectrum of compound **8** in $CDCl_3$ (expanded view of the 0.0–3.5 ppm region).

Reported structures of *N. nambi* in different culture conditions.

Table S1: Secondary metabolites from *Neonothopanus nambi*.

**No**	**Compound**	**Culture conditions of *N. nambi.***			
		a	*b*	*c*	*d*
Sesquiterpenoids and dimeric sesquiterpenoids					
**S1**	nambinone A	V	V	X	X
**S2**	axinysone B	X	X	V	X
**S3**	nambinone B	X	X	V	X
**S4**	nambinone C	V	V	X	X
**S5**	1-*epi*-nambinone B	V	X	X	X
**S6**	nambinone D	V	X	X	X
**S7**	4,8,14-trihydroxyilludala-2,6,8-triene	X	X	V	X
**9**	aurisin A	V	X	V	V
**S8**	aurisin G	V	X	V	X
**S9**	aurisin K	V	X	X	X
**S10**	aurisin Z	V	V	X	X
Terphenyls and benzoquinone					
**S11**	neonambiterphenyl A	X	X	V	X
**S12**	neonambiterphenyl B	X	X	V	X
**S13**	neonambiquinone A	X	X	V	X
Sesterterpenoids					
**1**	nambiscalarane B	X	X	X	V
**2**	nambiscalarane C	X	X	X	V
**3**	nambiscalarane D	X	X	X	V
**4**	nambiscalarane E	X	X	X	V
**5**	nambiscalarane F	X	X	X	V
**6**	nambiscalarane G	X	X	X	V
**7**	nambiscalarane H	X	X	X	V
**8**	nambiscalarane	X	V	X	V
Steroid					
**S14**	ergosta-6,22-diene-3*β*,5,8*α*-triol	X	V	X	X
Butyrolactone					
**S15**	trans-*α*-hydroxy-*γ*-phenylbutyrolactone	X	V	X	X
Other					
**S16**	methyl 4-butyramidobenzoate	X	V	X	X

[a]The cultured liquid of PW1 and PW2 was grown in potato dextrose broth (PDB).[1]

[b]The cultured mycelium was grown in yeast malt agar (YM Agar).[2]

[c]The cultured mycelium of PW3 was grown in potato dextrose broth (PDB).[3]

[d]The cultured mycelium of PW2 was grown in malt extract broth (MEB).

V compound is present. X compound is absent.

Figure S1: Chemical constituents from the *Neonothopanus nambi*.

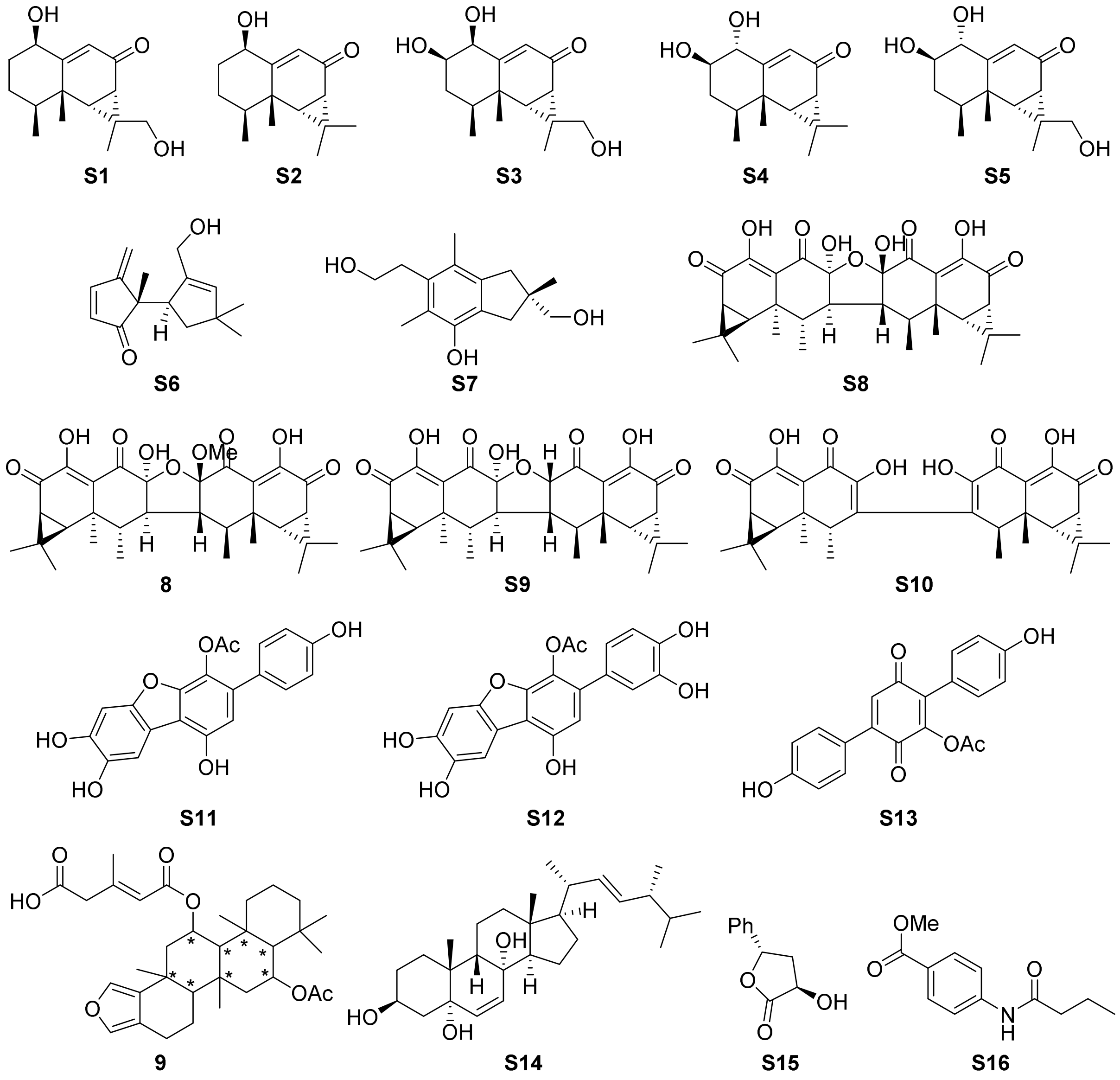

* = stereochemistry not specified in literature

Fungus material

The luminescent mushroom was collected in 2003 from the Plant Genetic Conservation Project under Royal Initiation by Her Royal Highness Princess Maha Chakri Sirindthorn at Kok Phutaka area, Wiang Kao District, Khon Kaen Province, and was identified by W. Saksirirat as *N. nambi*. The voucher specimens (PW1 and PW2) were deposited at the Department of Plant Science and Agricultural Resources, Faculty of Agriculture, Khon Kaen University, Khon Kaen, Thailand. The mushroom culture was incubated on potato dextrose broth in a dark room without shaking with 2 h of light per day at 25 °C for 30 days.[1]

Mycelium was cultivated at 27 °C on Petri dishes (90 mm diameter) using a non> buffered 2.0% (w/v) agar medium, containing 0.3% (w/v) malt extract (Difco), 0.3% (w/v) yeast extract (Helicon), 0.5% bacto peptone (Difco) and 1.0% of dextrose. From the Petri dishes the mycelium was inoculated and grown in submerged liquid cultures under orbital shaking (150 rpm) in 750 mL Erlenmeyer flasks in 250 mL of the same medium, without agar. Media were prepared using an autoclave set at 120 °C for 30 min and a laminar flow hood. Mycelium was harvested after 14 days at room temperature, emitting observable light in contact with air. Harvested culture was filtered, washed with deionized water, dried under vacuum and flushed with argon before use. Mycelium samples were grinded frozen with liquid nitrogen using a mortar and pestle or disrupted in the blender with extraction solvent.[2]

The luminous mushroom was collected in 2015 from the Plant Genetic Conservation Project under Royal Initiation by Her Royal Highness Princess Maha Chakri Sirindhorn at Kok Phutaka area, Wiang Kao District, Khon Kaen Province, Thailand and was identified by Prof. Weerasak Saksirirat as *N. Nambi* PW3. The voucher specimen was deposited at the Department of Plant Science and Agricultural Resources, Faculty of Agriculture, Khon Kaen University, Khon Kaen, Thailand. The mushroom was cultivated on potato dextrose broth without shaking with 2 h of light per day at 25 °C for 30 days.[3]

In this work, the mushroom strain PW2 was cultivated on malt extract broth without shaking with 2 h of light per day at 25 °C for 30 days.

Conformational Analysis and ECD Calculations of 2 and Its Enantiomer. For theoretical ECD spectra, possible configuration of compound **2** was established for both geometry optimizations and electronic excited calculations. Geometrical optimizations of their structures were taken under the density functional theory (DFT) calculations. These calculations were performed with hybrid density functional B3LYP, and using 6–311g(d,p) to diffuse basis set. In the single point energy calculations, the vertical transition energies to the valence excited–states were computed with the time–dependent density functional theory (TD–DFT) method using the long–range corrected functional CAM–B3LYP at the 6–311++g(d,p) level (σ = 0.30). The bulk solvent effects were evaluated using the Conductor–like Polarisable Continuum Model (C–PCM). All calculations were performed with GAUSSIAN 09 program.[4]

Figure S2: Experimental and calculated ECD spectra of **2** (black line, experimental ECD of **2**; red dashed line, calculated for *5S*, *6S*, *8S*, *9S*, *10S*, *11R*, *13S*, *14S* configurations; black dashed line, calculated for its enantiomer).

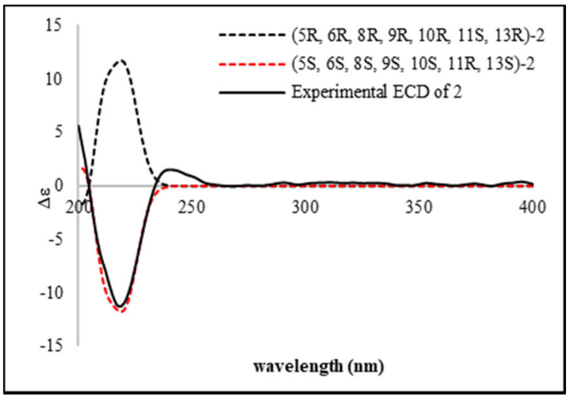

Figure S3: Structures of conformers of **2** computed by HyperChem.

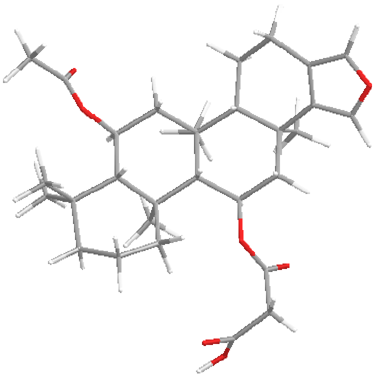

1 31.0%

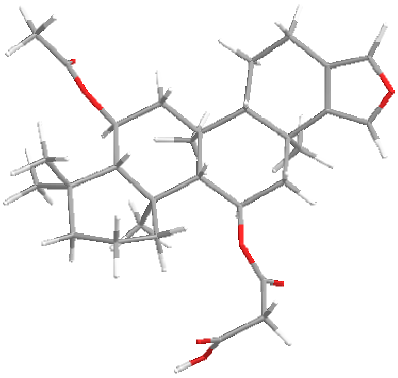

2 23.7%

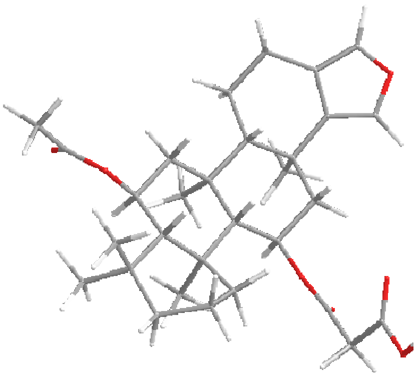

3 16.1%

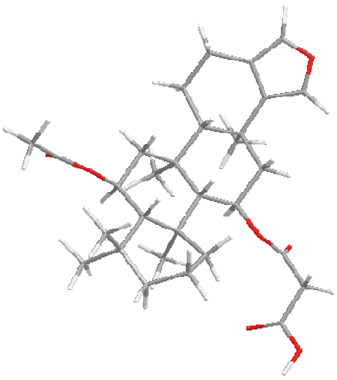

4 5.8%

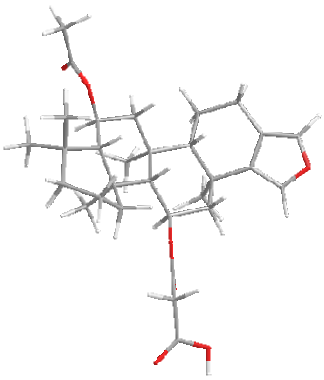

5 5.2%

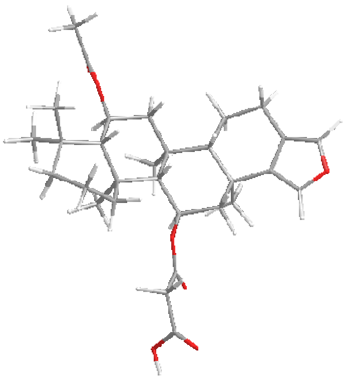

6 5.1%

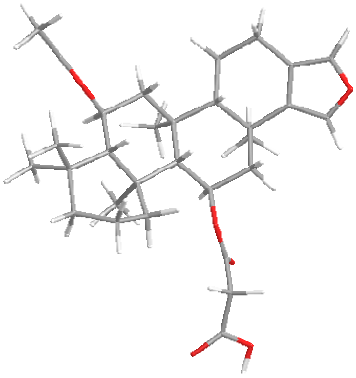

7 4.1%

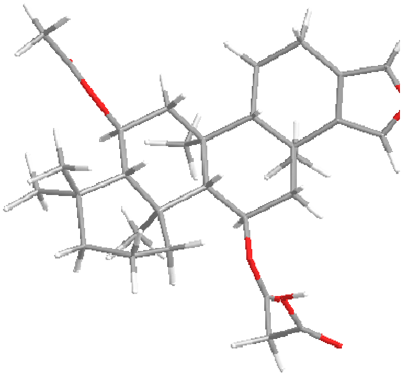

8 4.0%

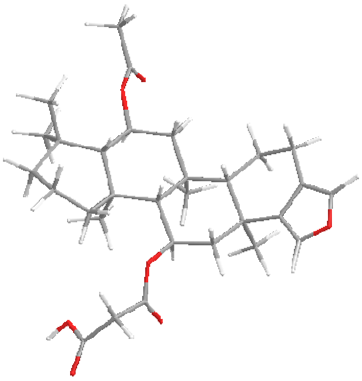

9 2.9%

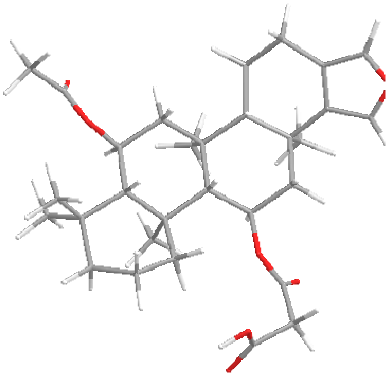

10 2.0%

Table S2: Conformer Boltzmann distribution of **2** computed by HyperChem.

Conformer[a]	ΔG (kcal/mol)	% Pop
**1**	0.00	31.0
**2**	0.16	23.7
**3**	0.39	16.1
**4**	1.00	5.8
**5**	1.07	5.2
**6**	1.08	5.1
**7**	1.20	4.1
**8**	1.22	4.0
**9**	1.41	2.9
**10**	1.63	2.0

[a]See **Figure S3** for conformers structures.

References

1. Kanokmedhakul, S.; Lekphrom, R.; Kanokmedhakul, K.; Hahnvajanawong, C.; Bua>art, S.; Saksirirat, W.; Prabpai, S.; Kongsaeree, P. Cytotoxic sesquiterpenes from luminescent mushroom *Neonothopanus nambi*. Tetrahedron **2012**, *68*:8261–8266.
2. Tsarkova, A. S.; Dubinnyi, M. A.; Baranov, M. S.; Oguienko, A. D.; Yampolsky, I. V.; Nambiscalarane, a novel sesterterpenoid comprising a furan ring, and other secondary metabolites from bioluminescent fungus *Neonothopanus nambi*. Mendeleev Commun. 2016, *26*:191–192.
3. Sangsopha, W.; Lekphrom, R.; Schevenels, F. T.; Saksirirat, W.; Bua>Art, S.; Kanokmedhakul, K,; Kanokmedhakul, S. New p >terphenyl and benzoquinone metabolites from the bioluminescent mushroom *Neonothopanus nambi*. Nat. Prod. Res. **2019**. 10.1080/14786419.2019.1578763.
4. Frisch, M. J.; Trucks, G. W.; Schlegel, H. B.; Scuseria, G. E.; Robb, M. A.; Cheeseman, J. R.; Scalmani, G.; Barone, V.; Mennucci, B.; Petersson, G. A.; Nakatsuji, H.; Caricato, M.; Li, X.; Hratchian, H. P.; Izmaylov, A. F.; Bloino, J.; Zheng, G.; Sonnenberg, J. L.; Hada, M.; Ehara, M.; Toyota, K.; Fukuda, R.; Hasegawa, J.; Ishida, M.; Nakajima, T.; Honda, Y.; Kitao, O.; Nakai, H.; Vreven, T.; Montgomery, J. A.; Peralta, J. E.; Ogliaro, F.; Bearpark, M.; Heyd, J. J.; Brothers, E.; Kudin, K. N.; Staroverov, V. N.; Kobayashi, R.; Normand, J.; Raghavachari, K.; Rendell, A.; Burant, J. C.; Iyengar, S. S.; Tomasi, J.; Cossi, M.; Rega, N.; Millam, J. M.; Klene, M.; Knox, J. E.; Cross, J. B.; Bakken, V.; Adamo, C.; Jaramillo, J.; Gomperts, R.; Stratmann, R. E.; Yazyev, O.; Austin, A. J.; Cammi, R.; Pomelli, C.; Ochterski, J. W.; Martin, R. L.; Morokuma, K.; Zakrzewski, V. G.; Voth, G. A.; Salvador, P.; Dannenberg, J. J.; Dapprich, S.; Daniels, A. D.; Farkas; Foresman, J. B.; Ortiz, J. V.; Cioslowski, J.; Fox, D. J. *Gaussian 09, Revision B.01*; Gaussian, Inc.: Walling.

Original spectroscopic data of 1-8.

Figure S4: IR spectrum of compound **1**.

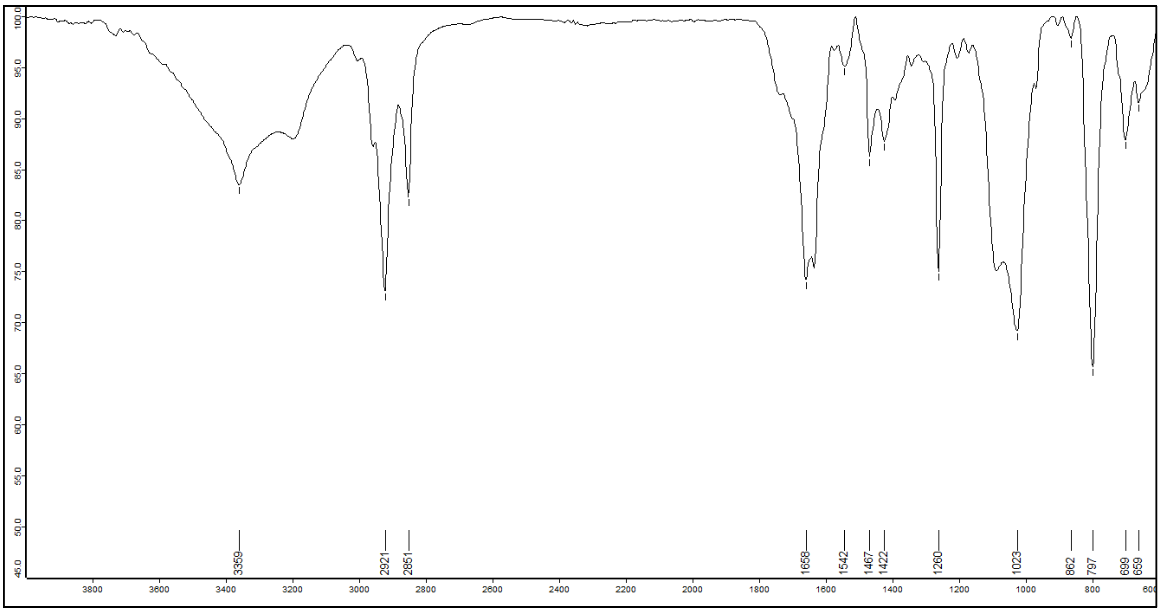

Figure S5: UV spectrum of **1** in methanol.

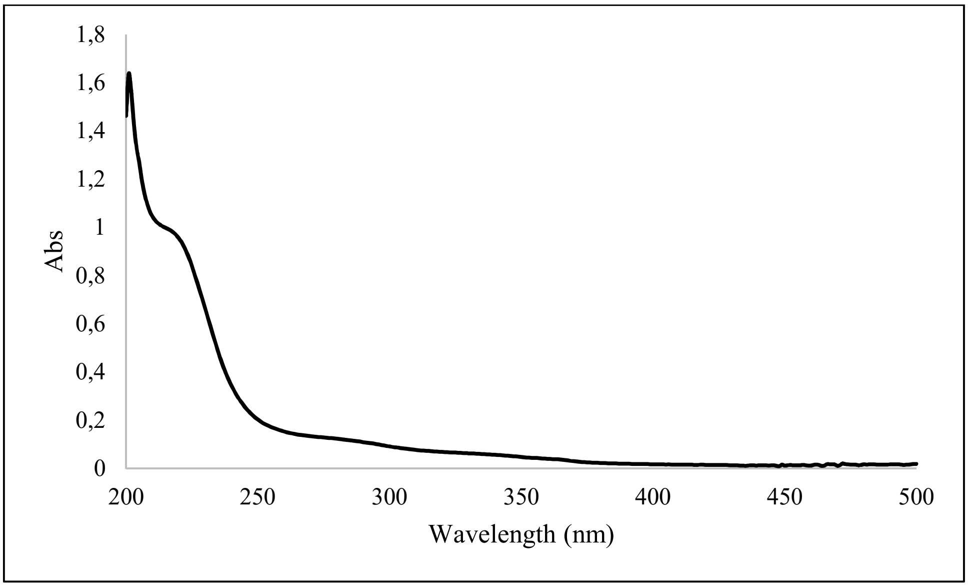

Figure S6: HRESIMS spectrogram of compound **1**.

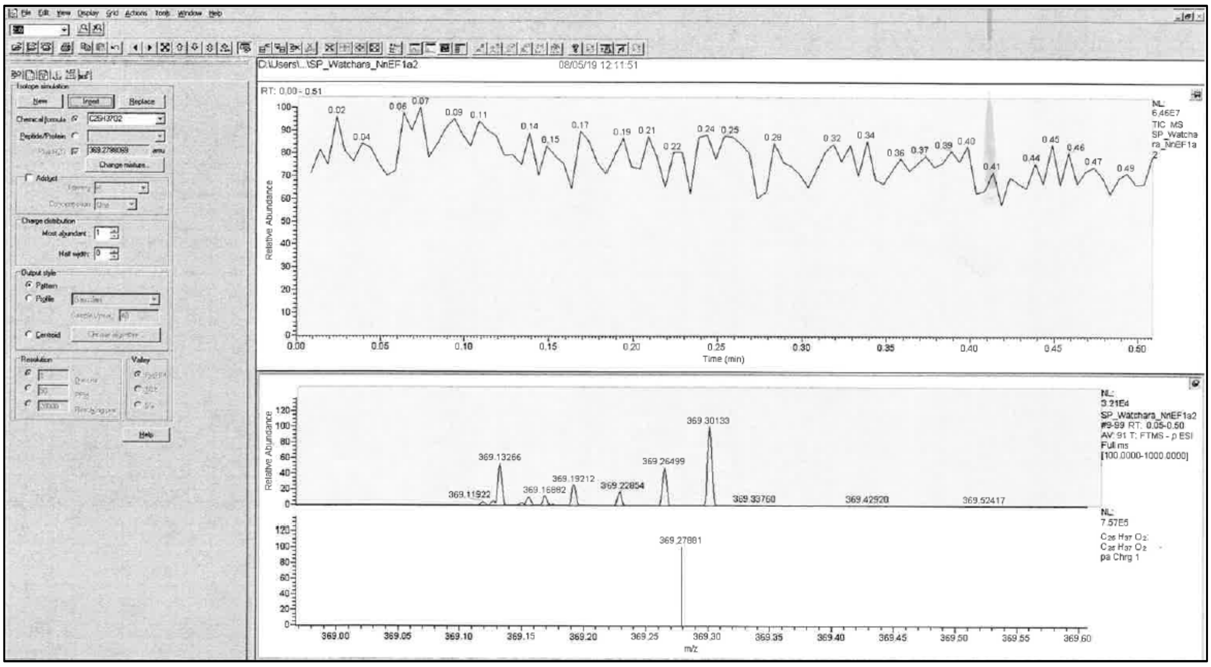

Figure S7: ^{1}H NMR spectrum of compound **1** in $CDCl_3$.

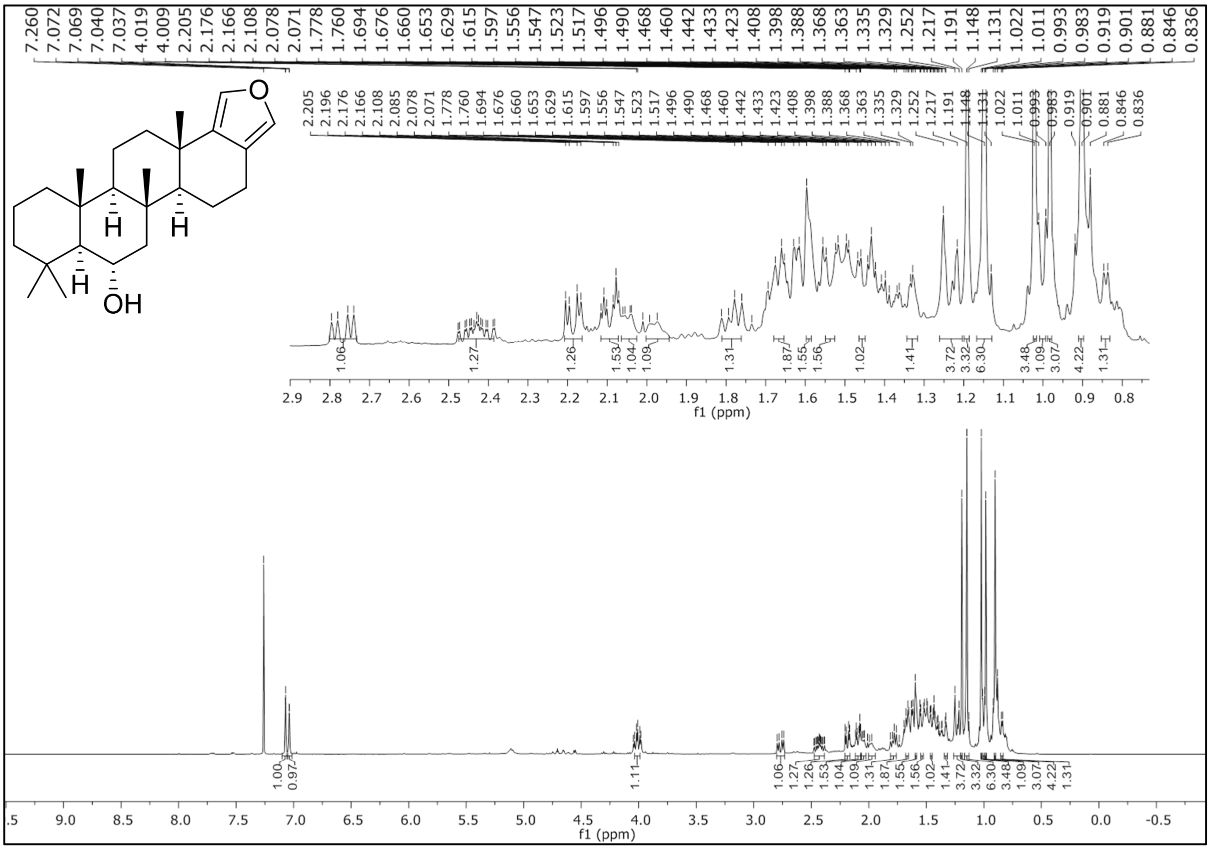

Figure S8: ^{13}C NMR spectrum of compound **1** in $CDCl_3$.

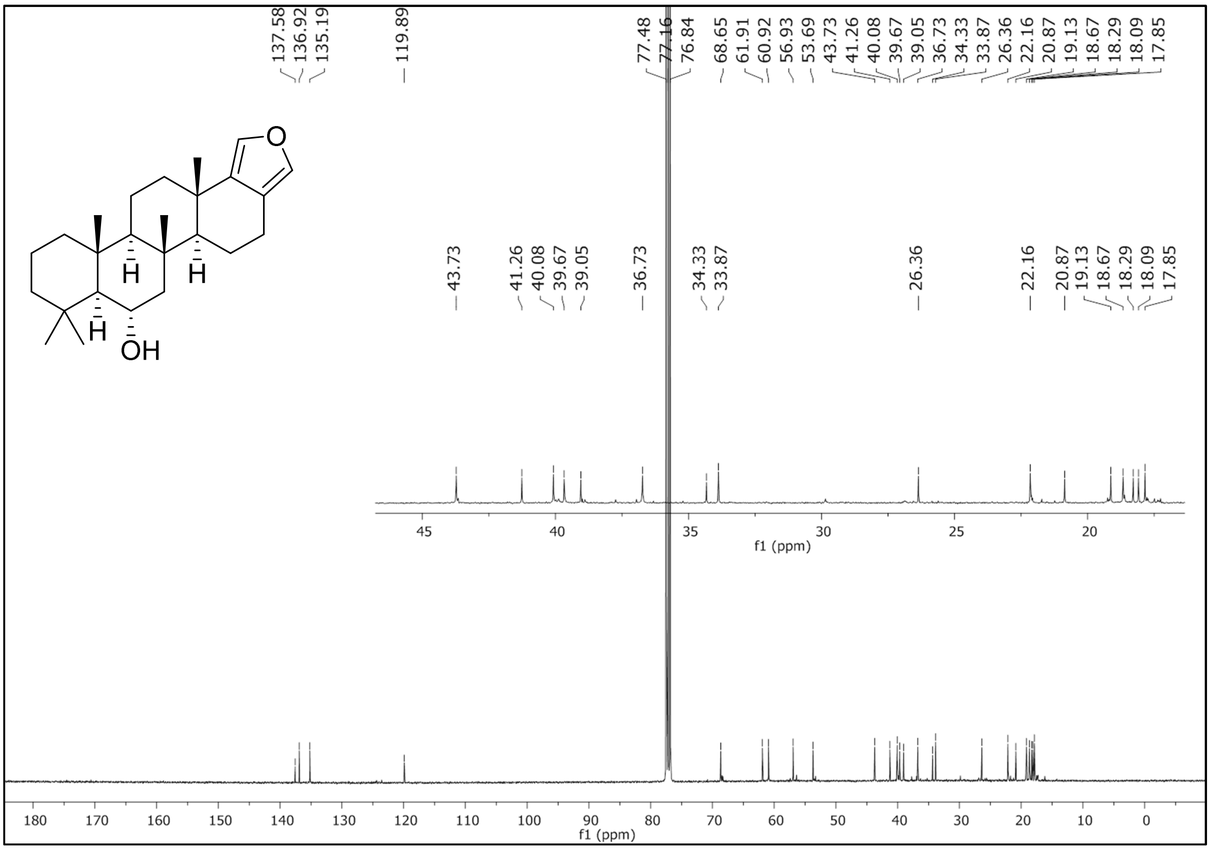

Figure S9: DEPTQ spectrum of compound **1** in $CDCl_3$.

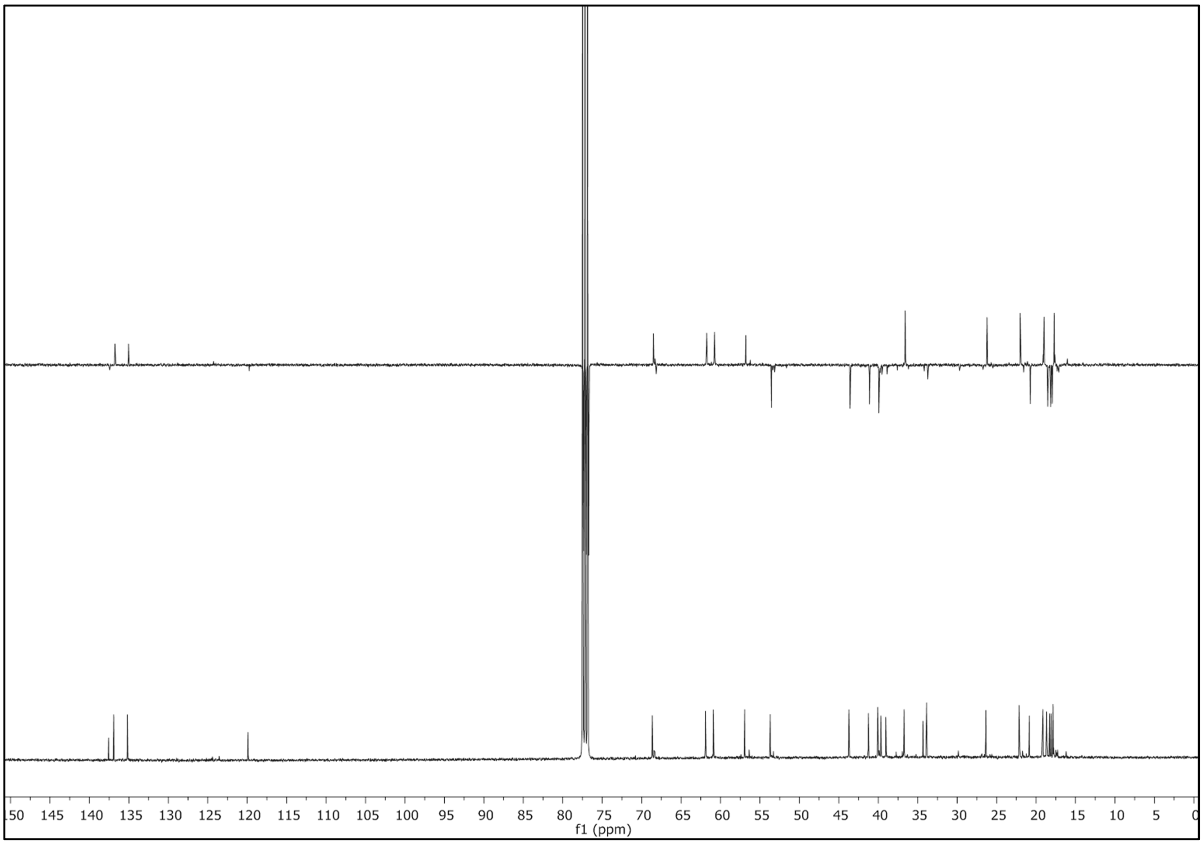

Figure S10: ^{1}H-^{1}H COSY NMR spectrum of compound **1** in $CDCl_3$.

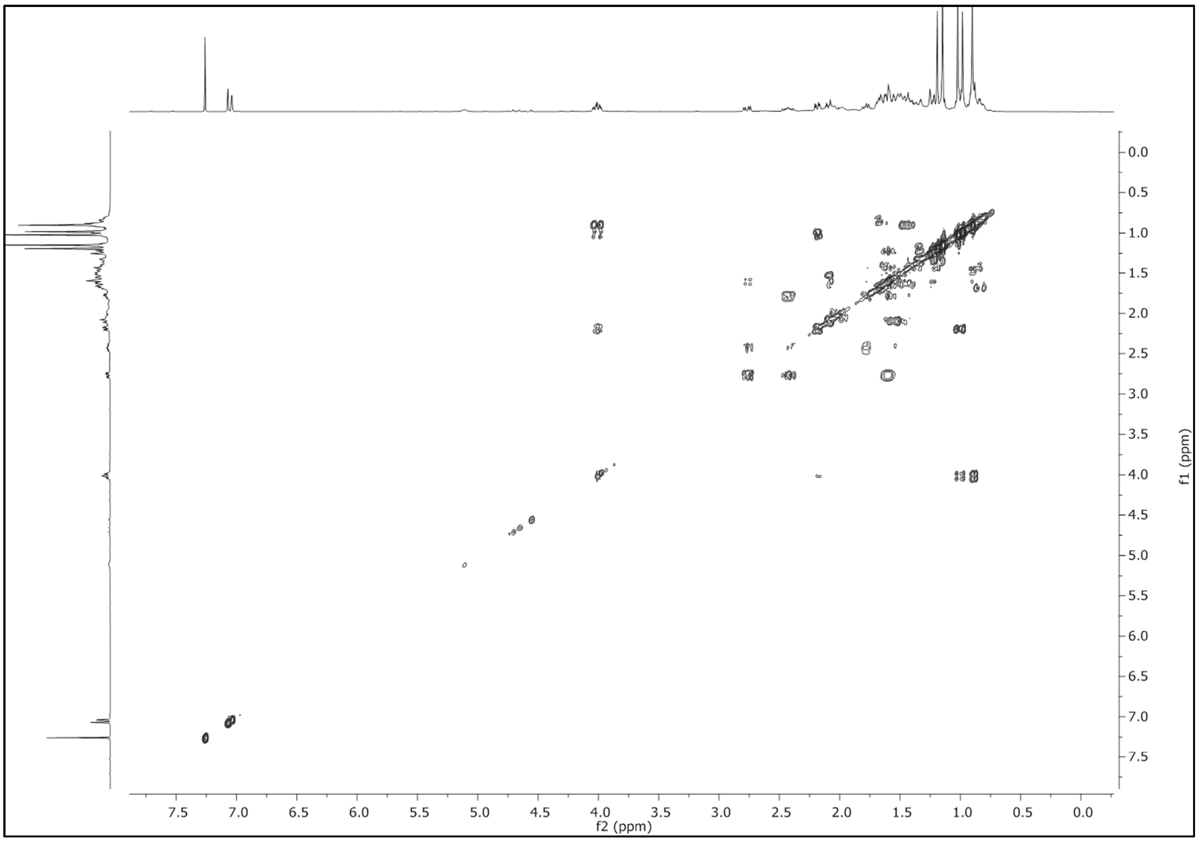

Figure S11: HSQC NMR spectrum of compound **1** in $CDCl_3$.

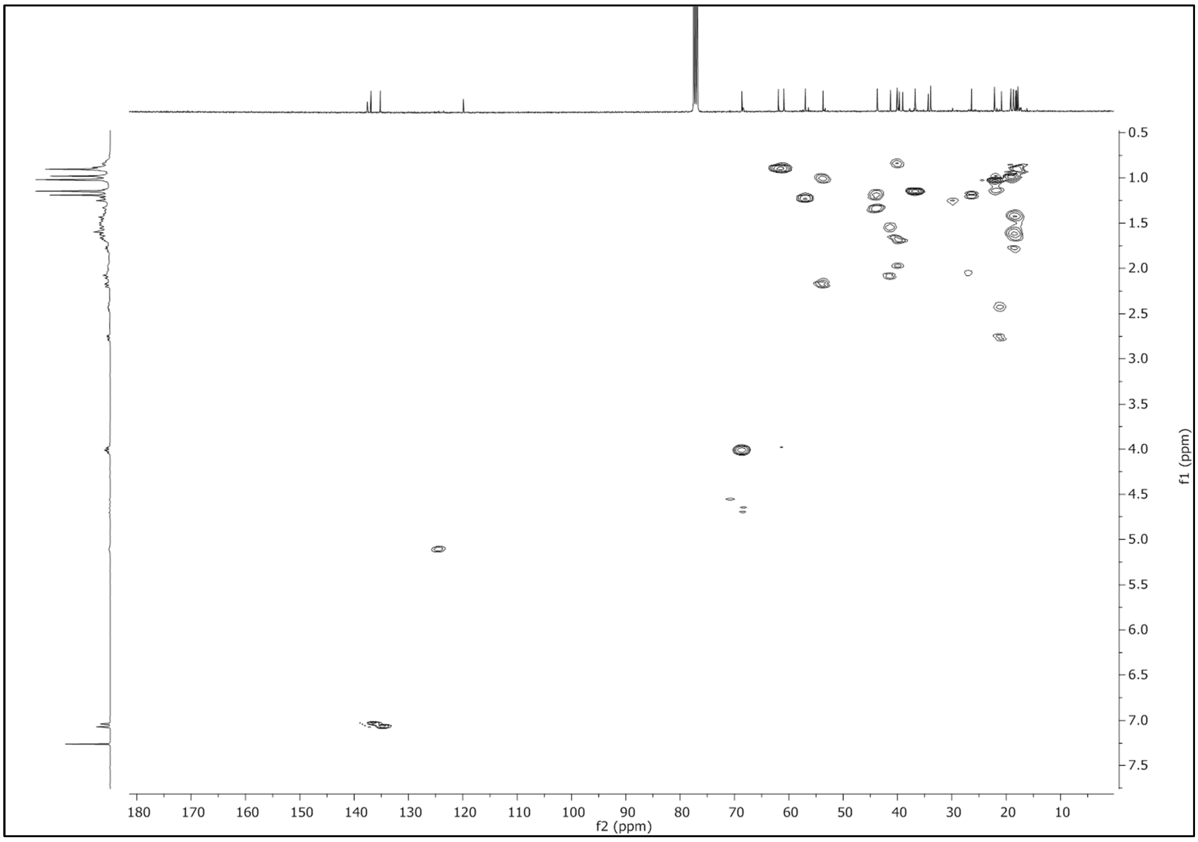

Figure S12: HMBC NMR spectrum of compound **1** in $CDCl_3$.

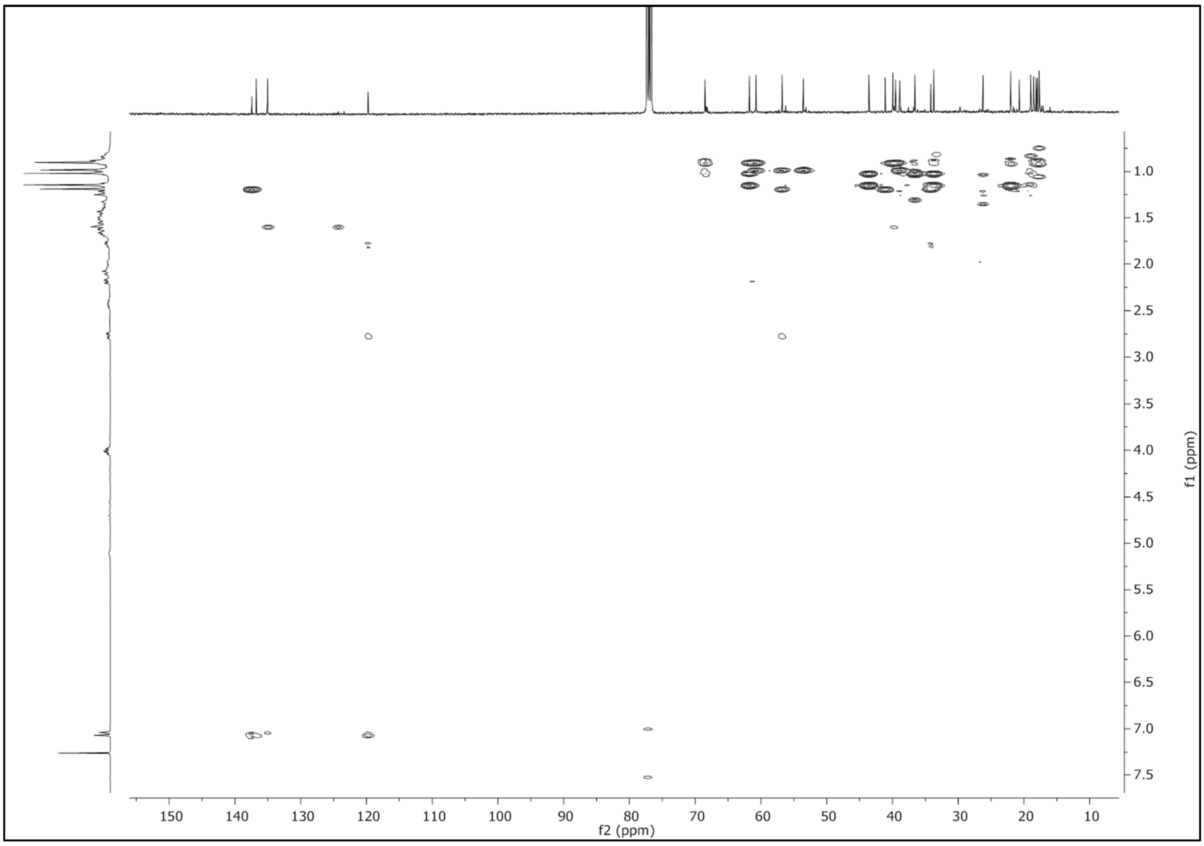

Figure S13: ^1H-^1H NOESY NMR spectrum of compound **1** in $CDCl_3$ (overview).

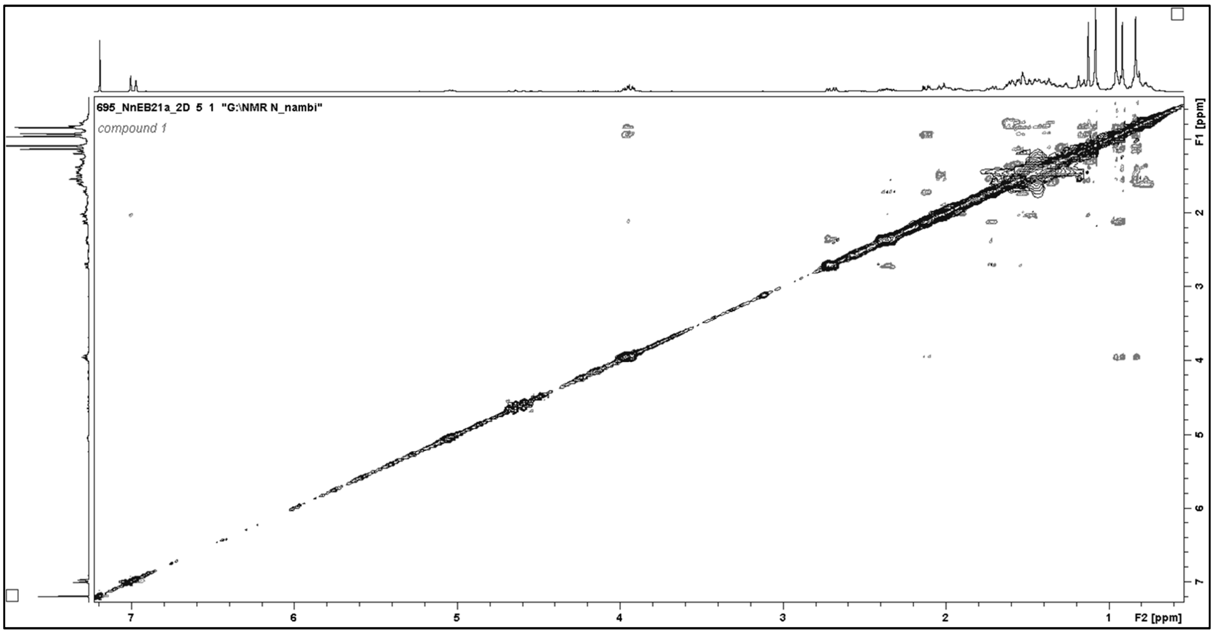

Figure S14: ^{1}H–^{1}H NOESY NMR spectrum of compound **1** in $CDCl_3$ (expanded view of the 0.0–4.5 ppm region).

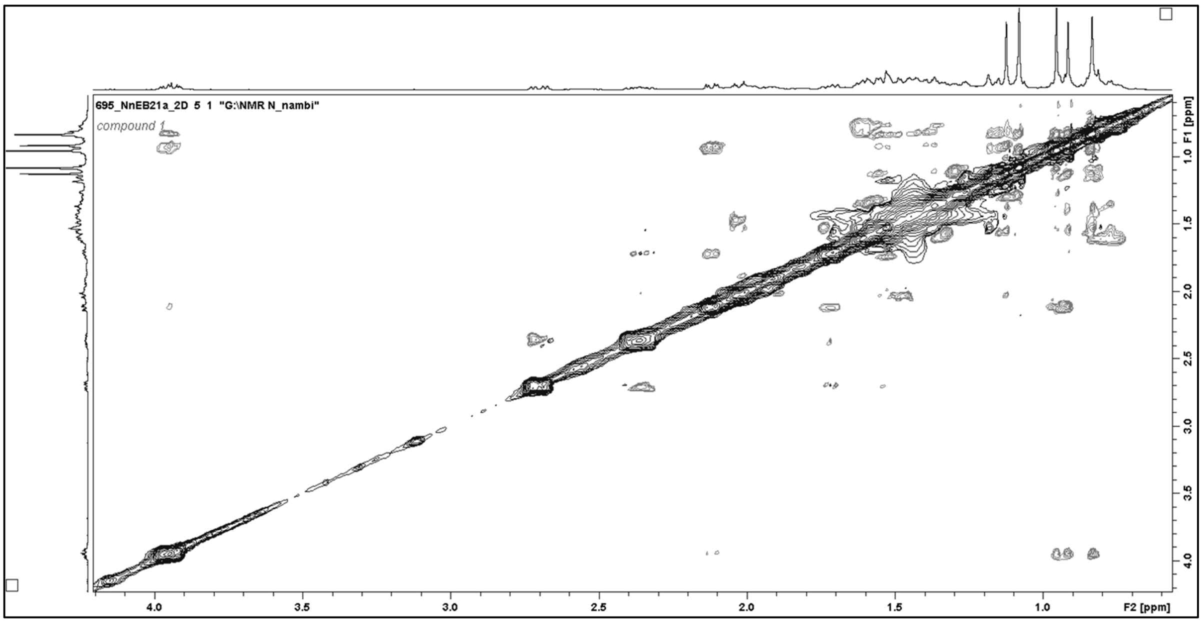

Figure S15: IR spectrum of compound **2**.

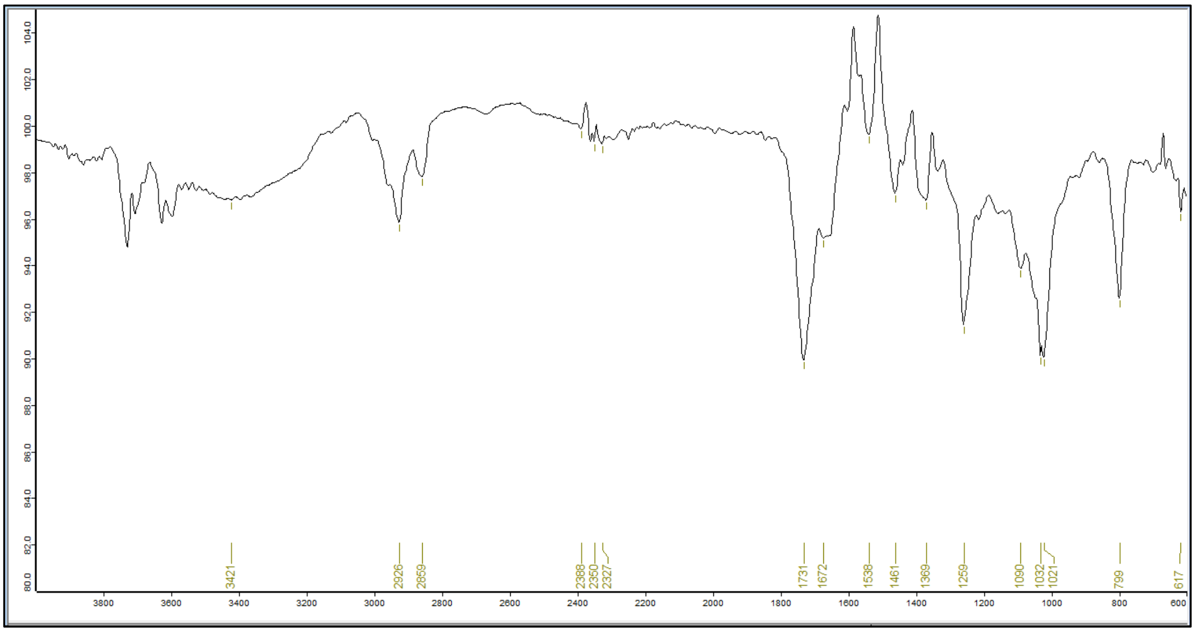

Figure S16: UV spectrum of **2** in methanol.

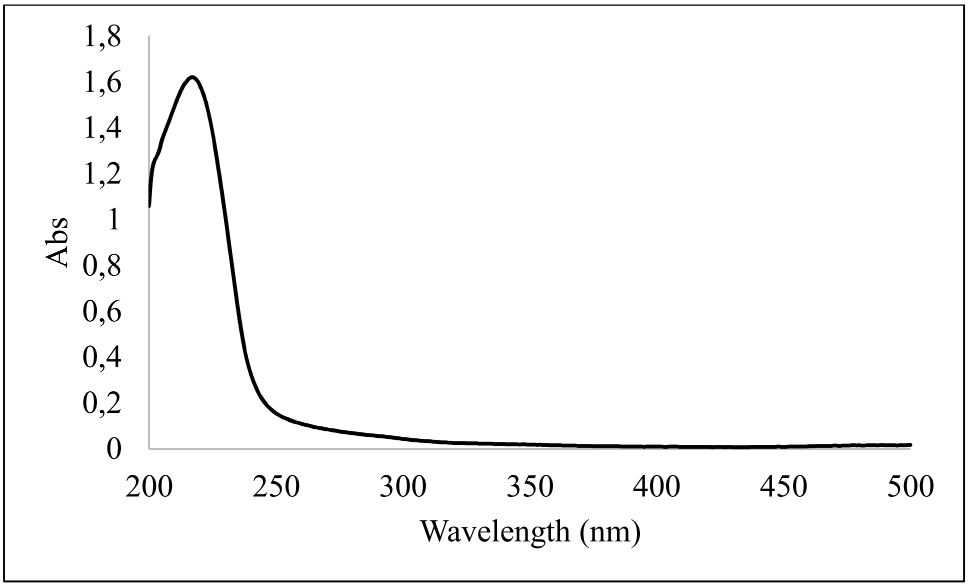

Figure S17: HRESIMS spectrogram of compound **2**.

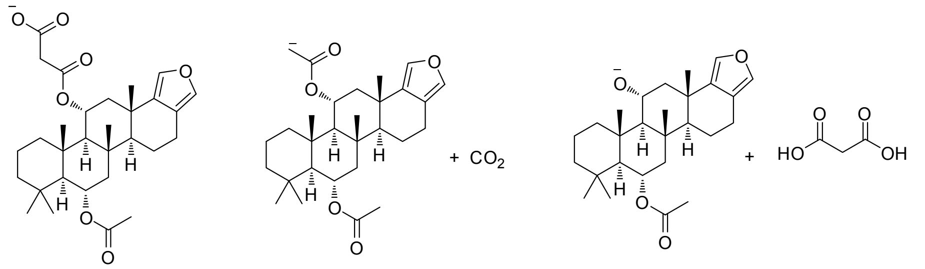

[M - H]$^-$ = 513.2846
calcd. = 513.2858

[M - CO_2]$^-$ = 469.2953
calcd. = 469.2959

[M - $C_3H_4O_4$]$^-$ = 427.2835
calcd. = 427.2854

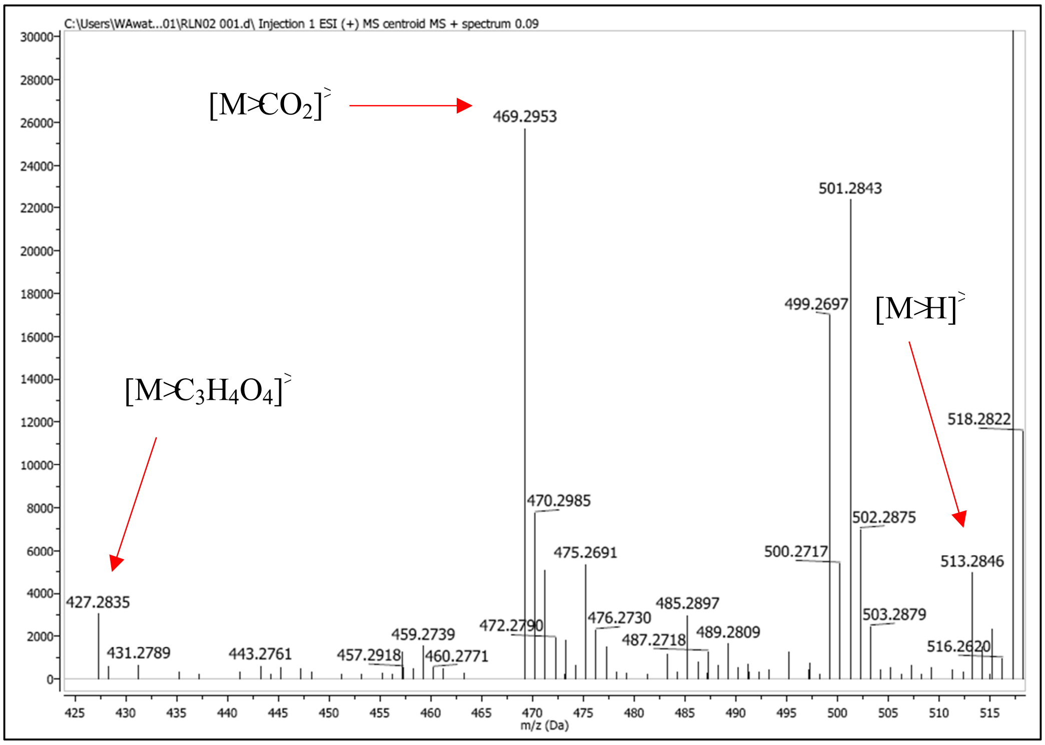

Figure S18: ^{1}H NMR spectrum of compound **2** in $CDCl_3$.

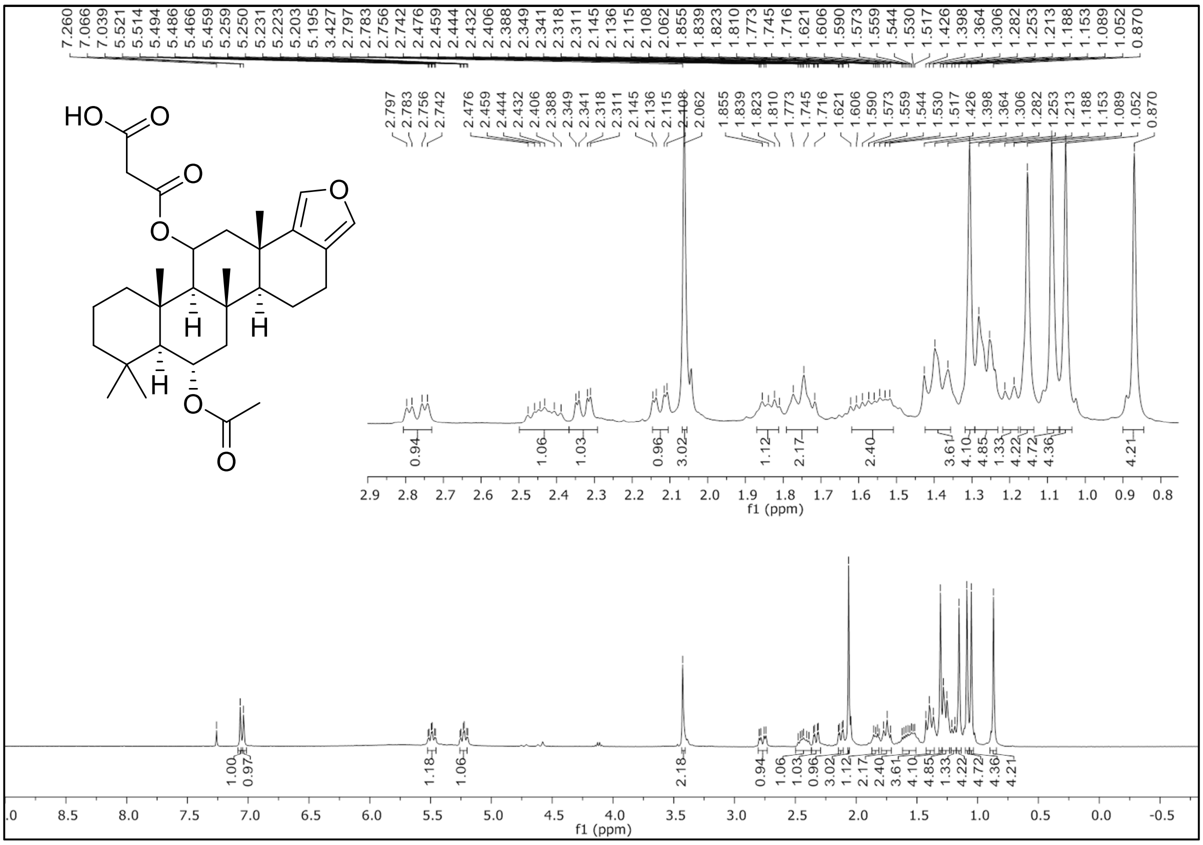

Figure S19: ^{13}C NMR spectrum of compound **2** in $CDCl_3$.

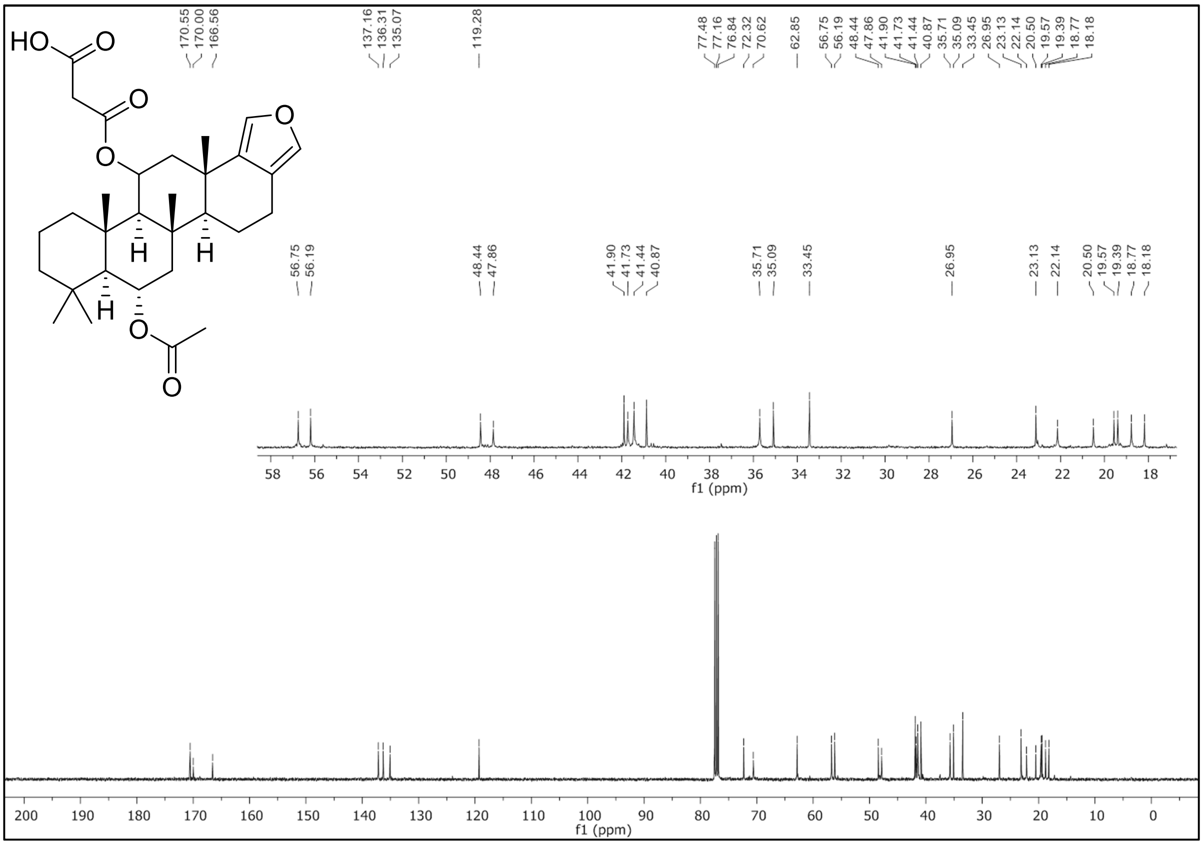

Figure S20: DEPT spectrum of compound **2** in $CDCl_3$.

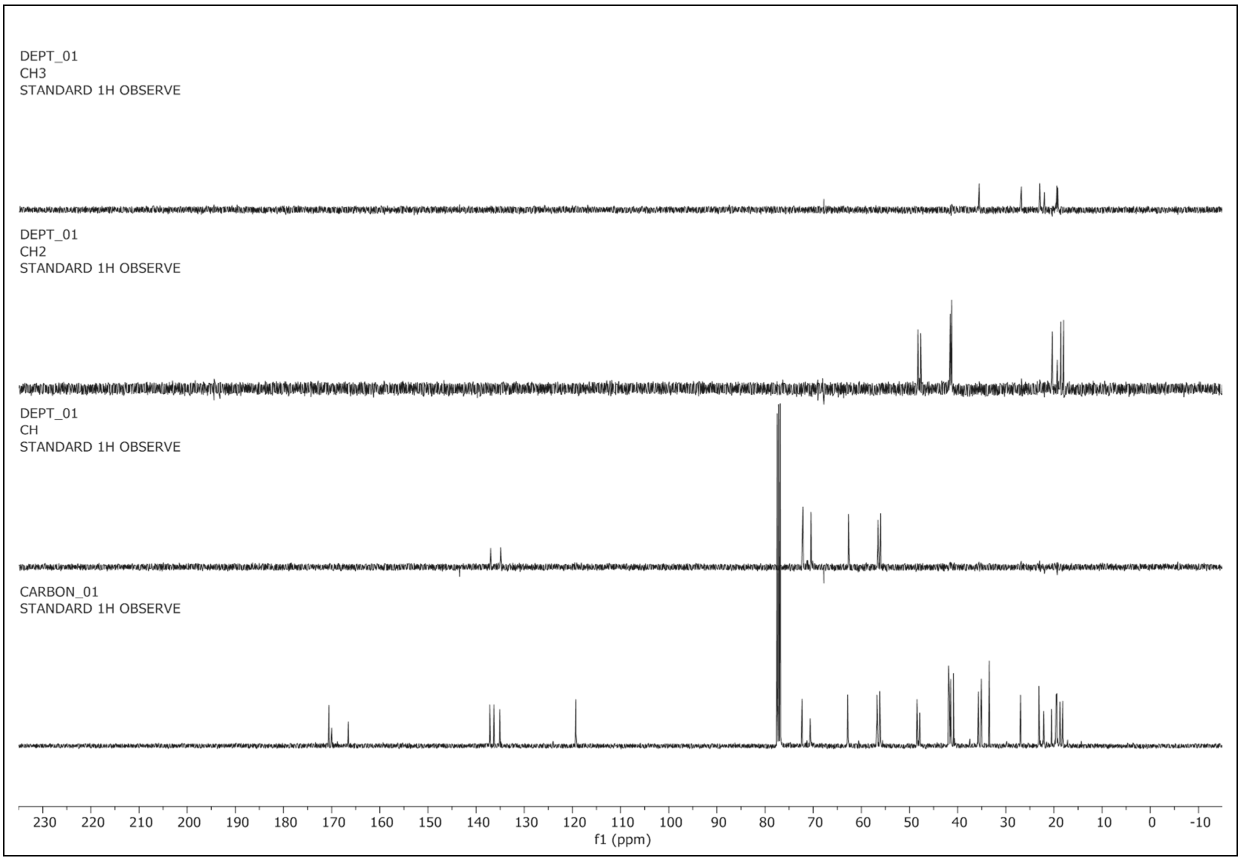

Figure S21: ^{1}H-^{1}H COSY NMR spectrum of compound **2** in $CDCl_3$.

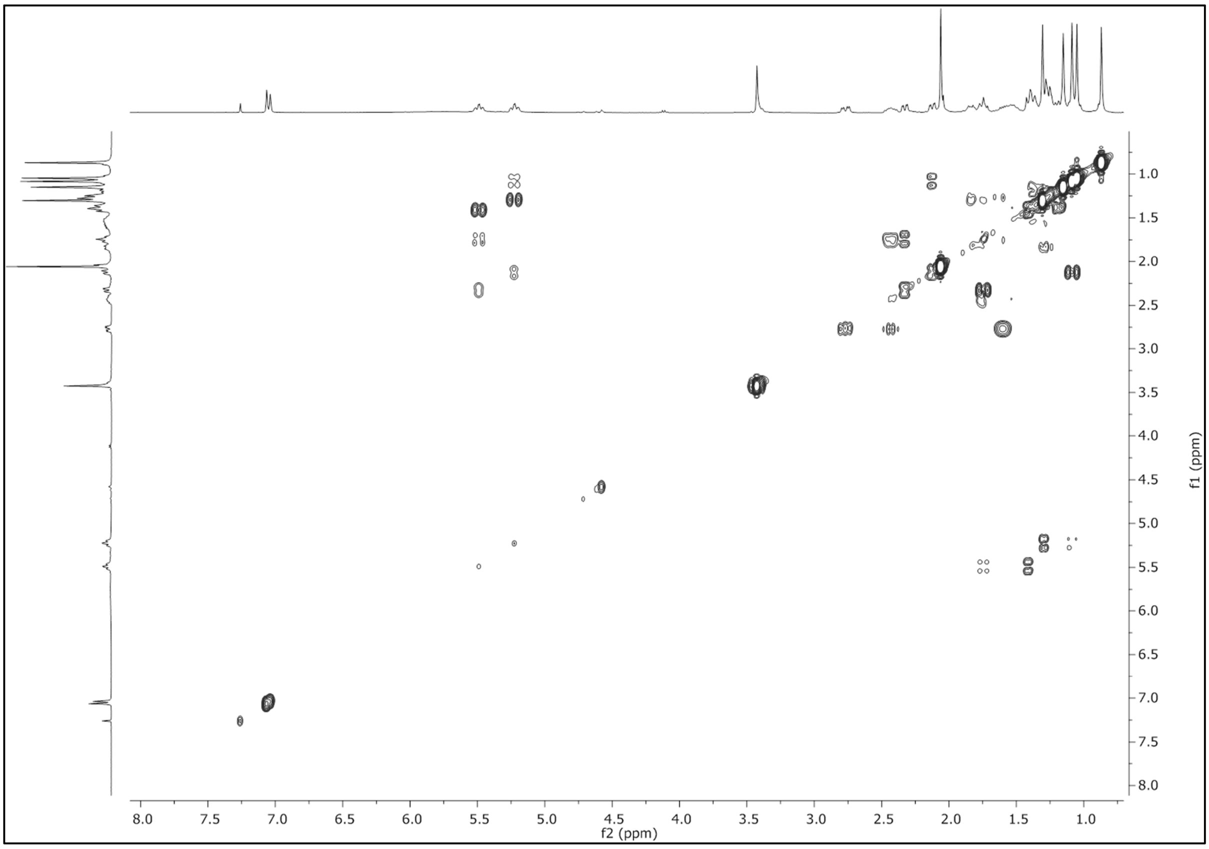

Figure S22: HSQC spectrum of compound **2** in $CDCl_3$.

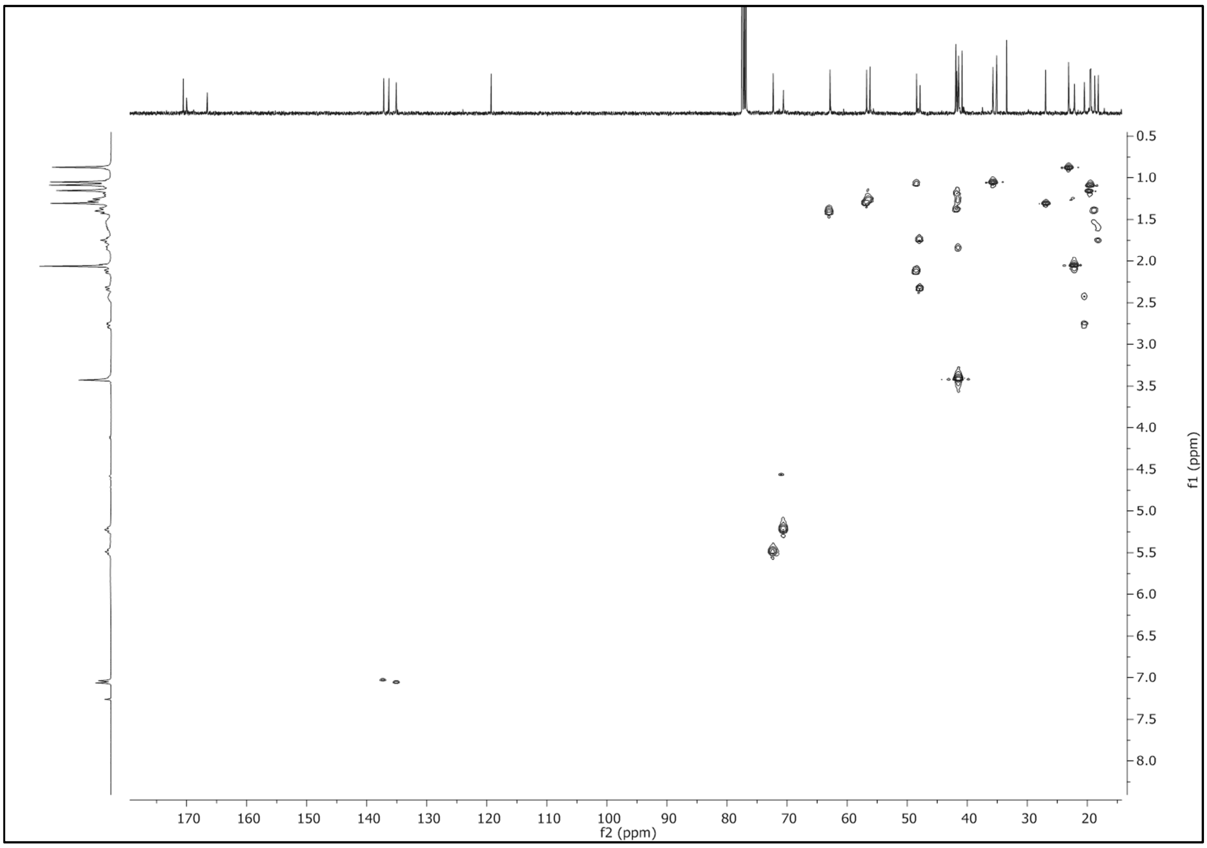

Figure S23: HMBC spectrum of compound **2** in $CDCl_3$.

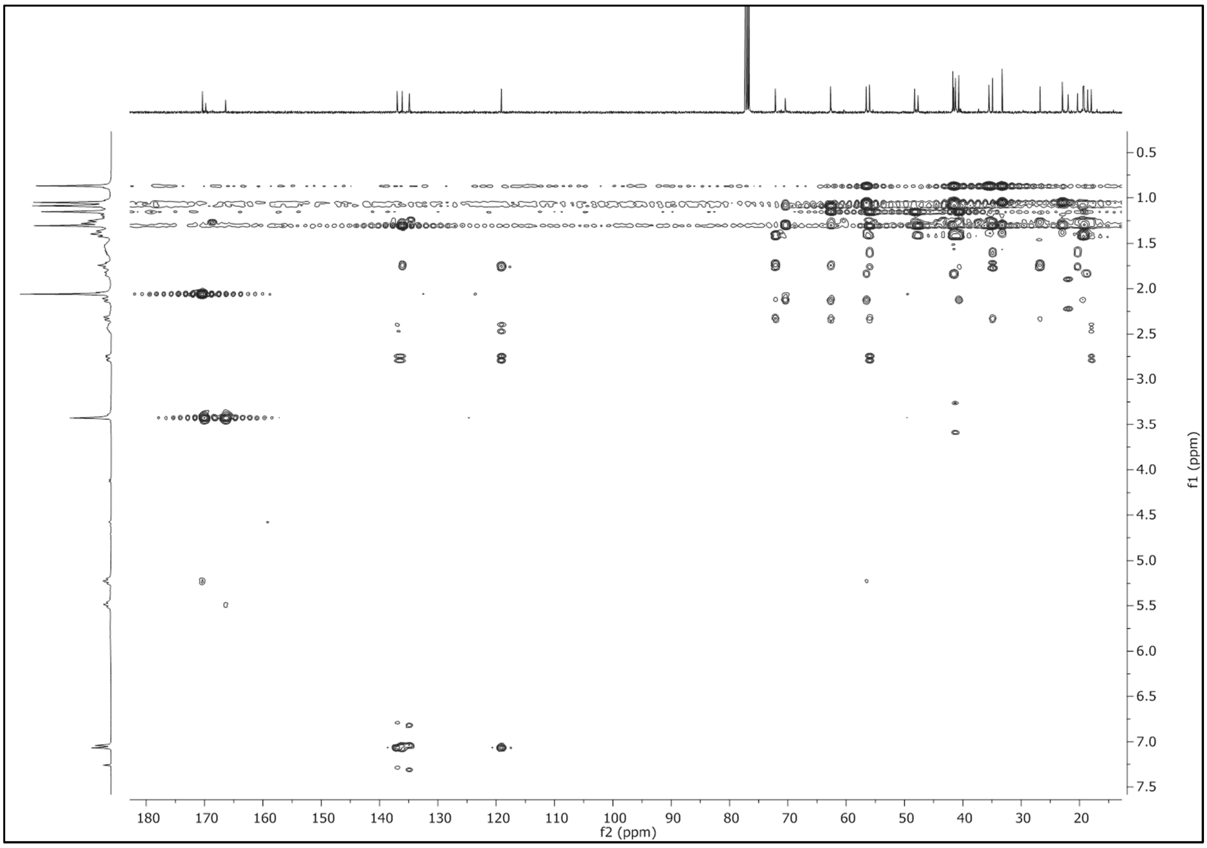

Figure S24: ^{1}H–^{1}H NOESY NMR spectrum of compound **2** in $CDCl_3$ (overview).

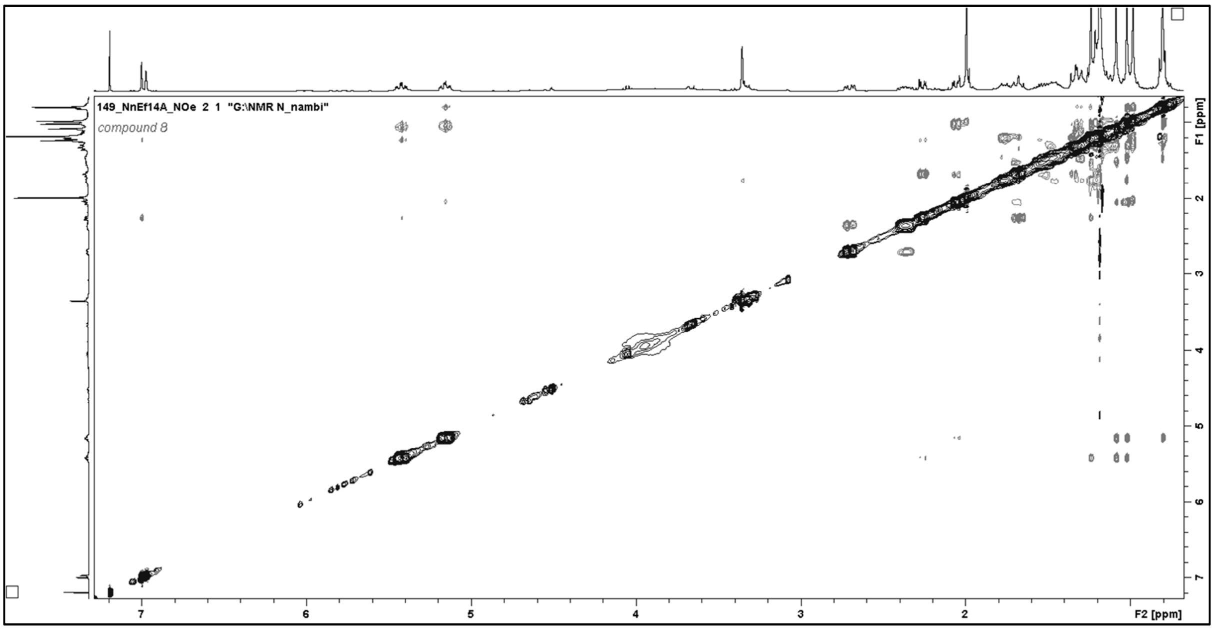

Figure S25: ^{1}H–^{1}H NOESY NMR spectrum of compound **2** in $CDCl_3$ (expanded view of the 0.0–6.0 ppm region).

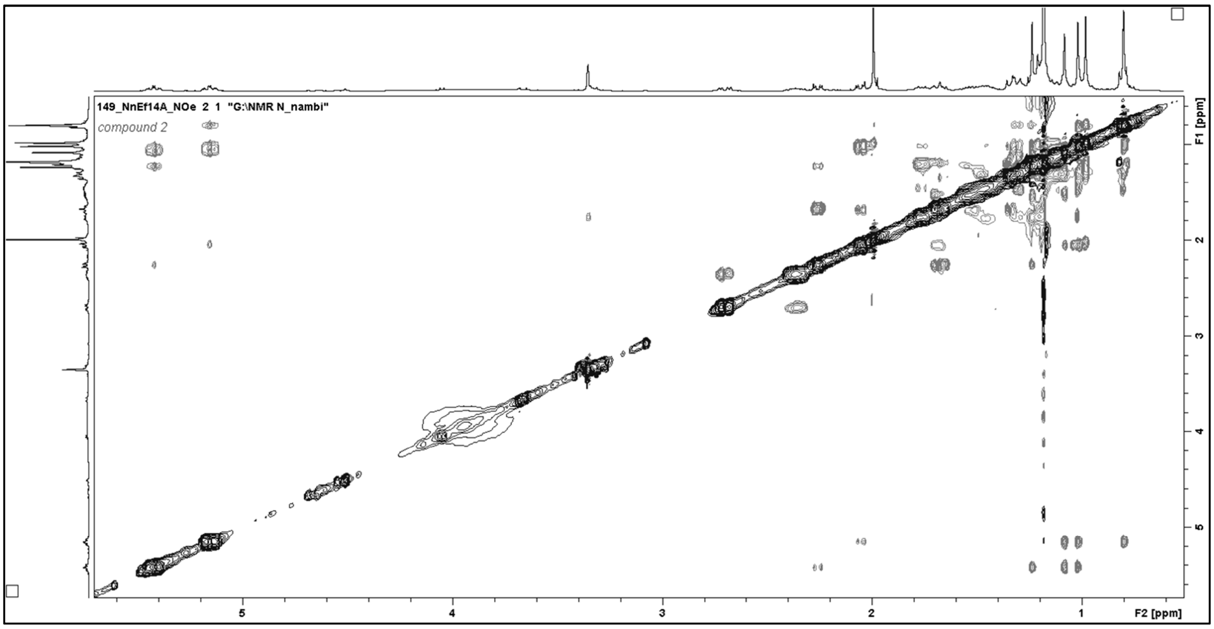

Figure S26: ^{1}H–^{1}H NOESY NMR spectrum of compound **2** in $CDCl_3$ (expanded view of the 0.0–3.0 ppm region).

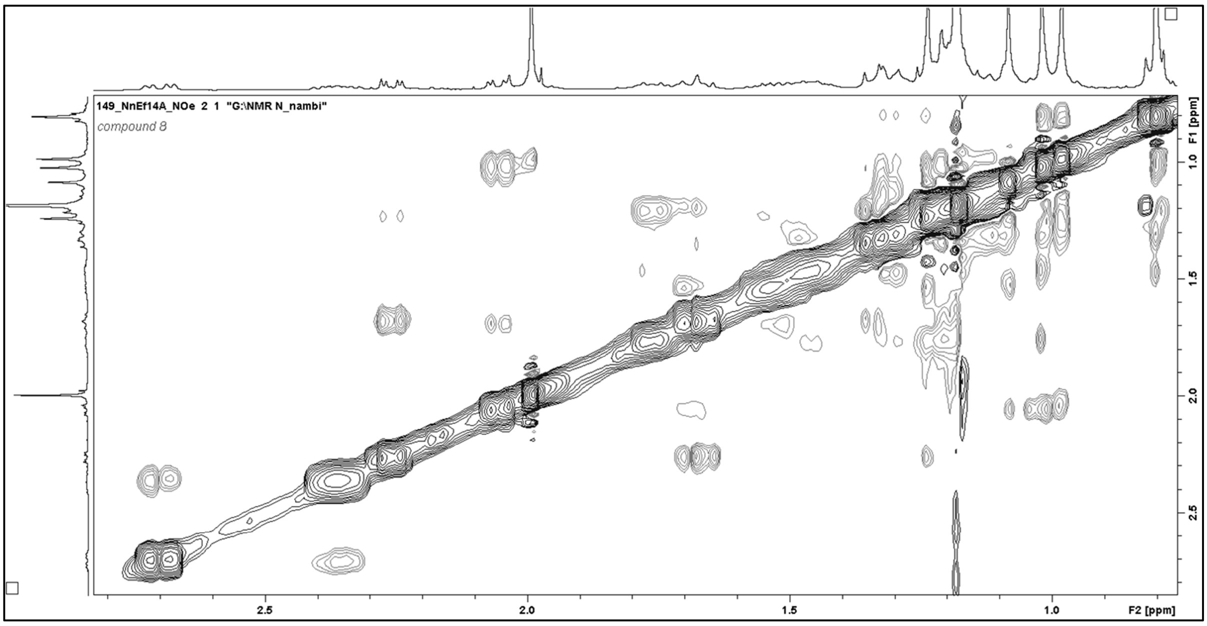

Figure S27: IR spectrum of compound **3**.

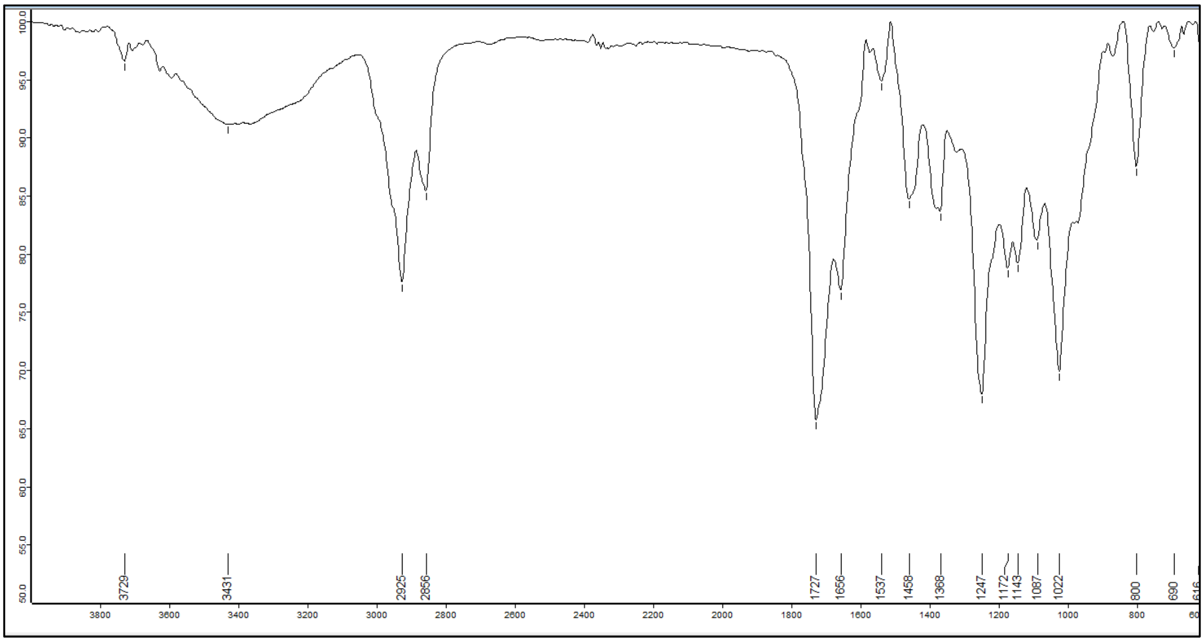

Figure S28: UV spectrum of compound **3** in methanol.

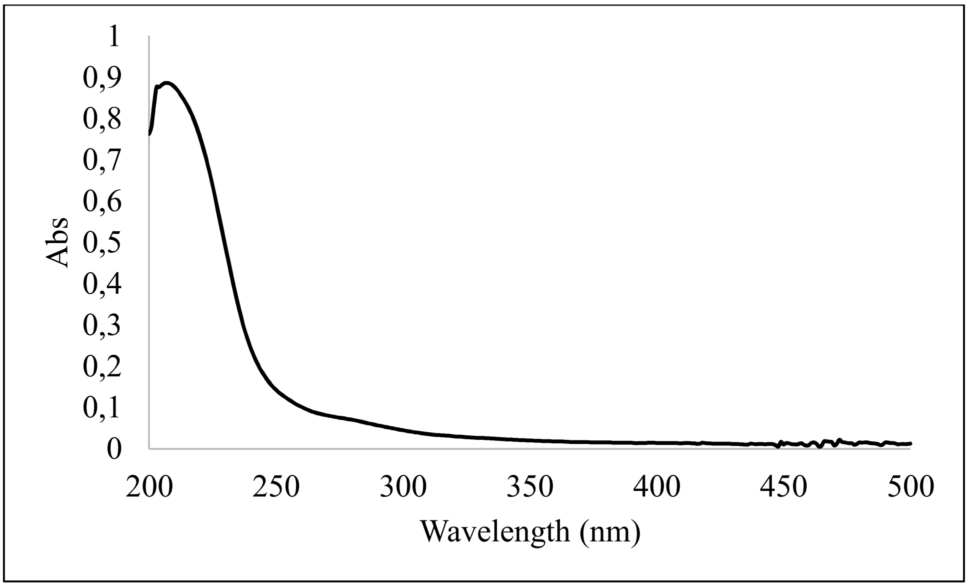

Figure S29: HRESIMS spectrogram of compound **3**.

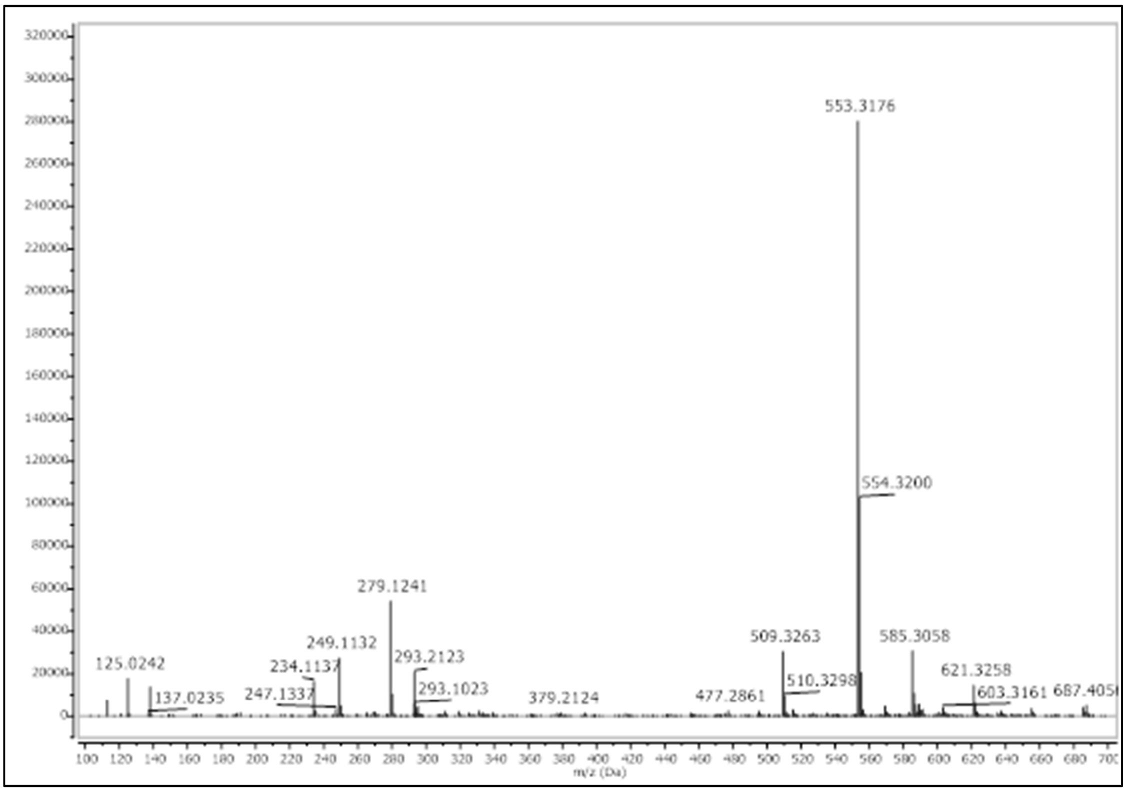

Figure S30: ^{1}H NMR spectrum of compound **3** in $CDCl_3$.

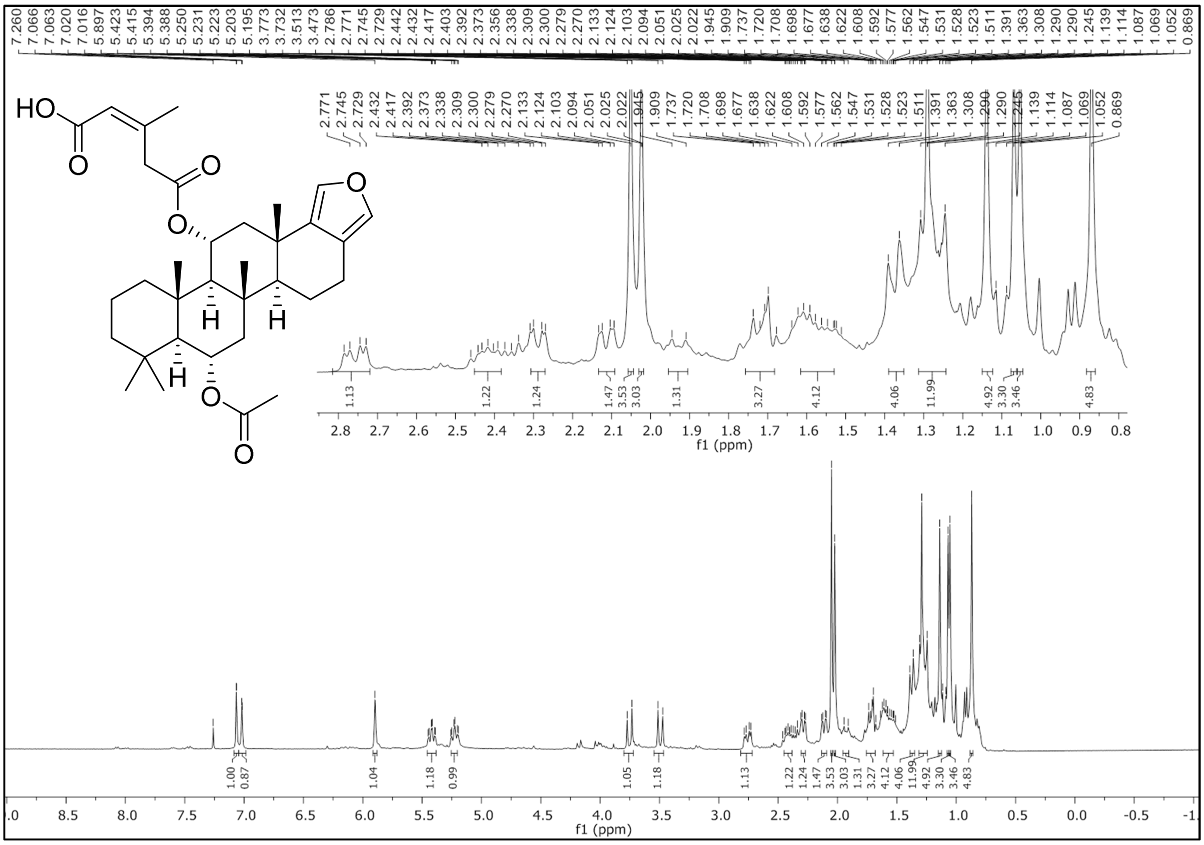

Figure S31: ^{13}C NMR spectrum of compound **3** in $CDCl_3$.

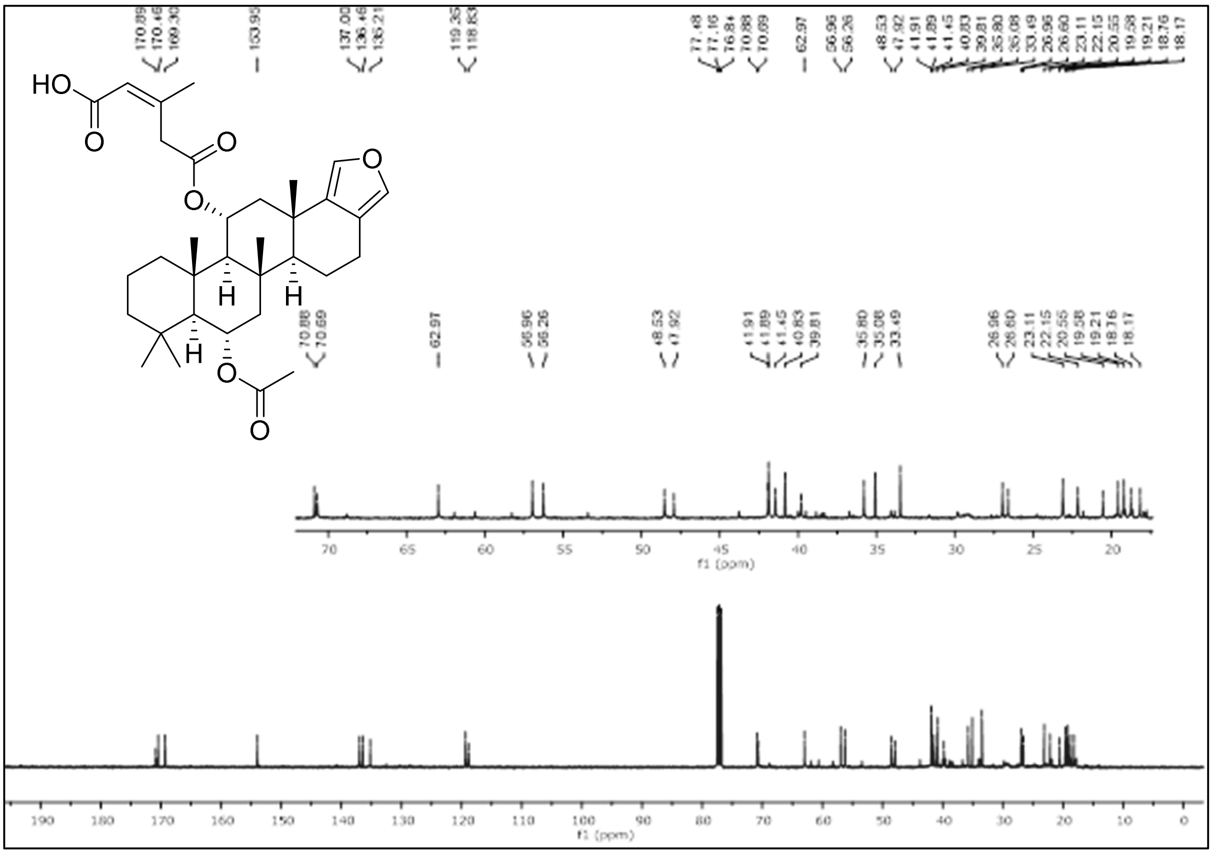

Figure S32: DEPT spectrum of compound **3** in $CDCl_3$.

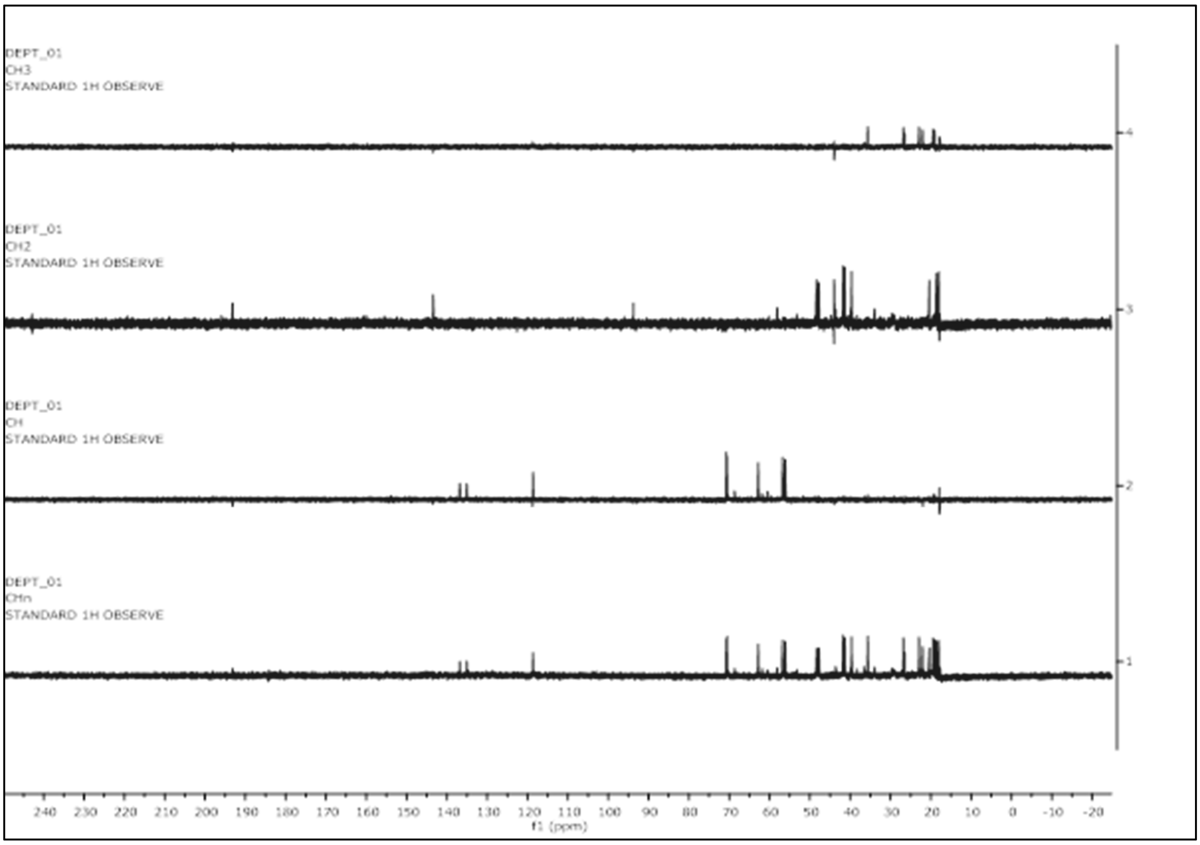

Figure S33: ^{1}H-^{1}H COSY NMR spectrum of compound **3** in $CDCl_3$.

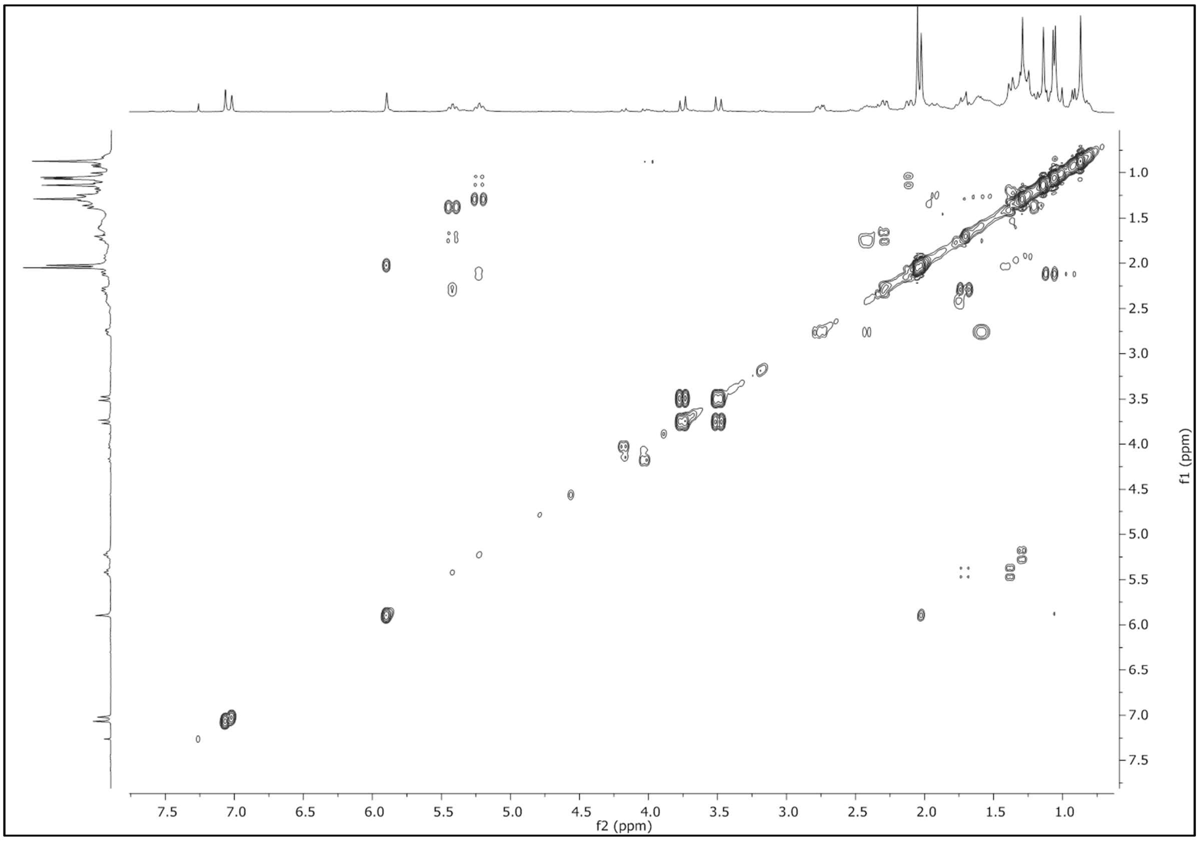

Figure S34: HSQC spectrum of compound **3** in $CDCl_3$.

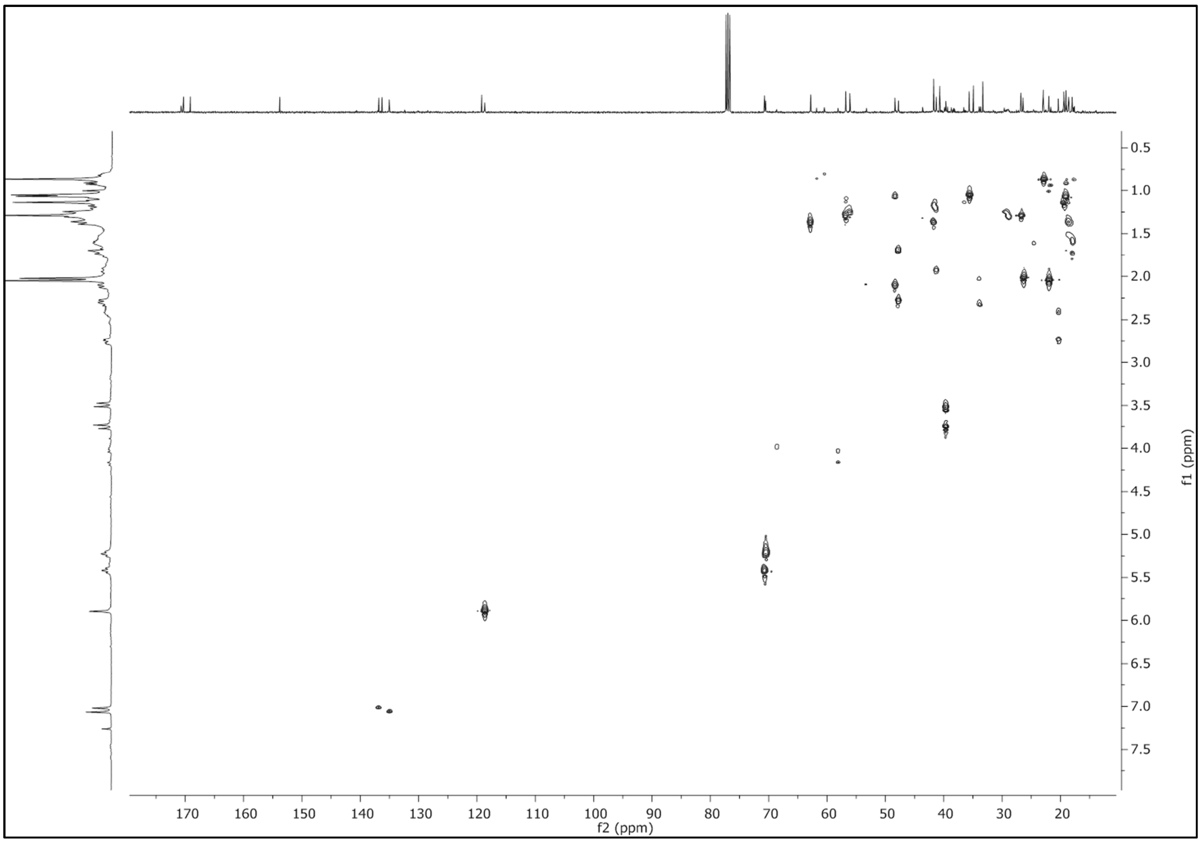

Figure S35: HMBC spectrum of compound **3** in $CDCl_3$.

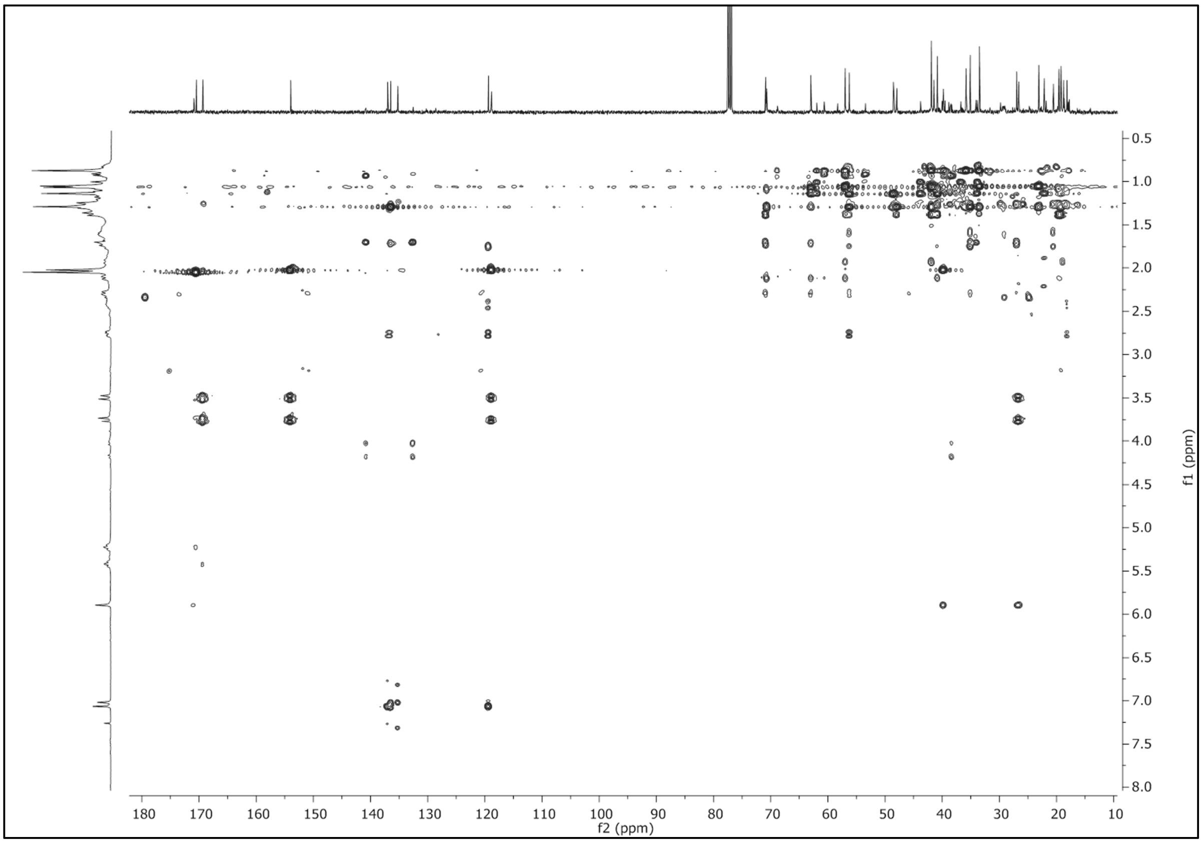

Figure S36: ^{1}H–^{1}H NOESY NMR spectrum of compound **3** in $CDCl_3$ (overview).

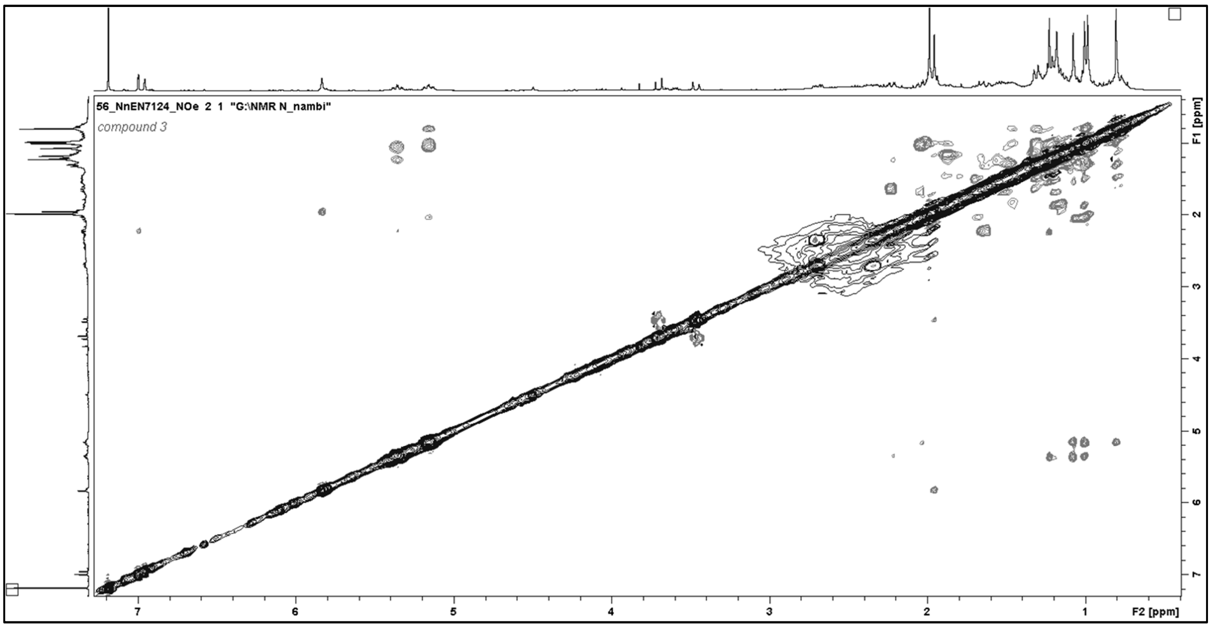

Figure S37: ^{1}H–^{1}H NOESY NMR spectrum of compound **3** in $CDCl_3$ (expanded view of the 0.1–2.9 × 5.05–5.55 ppm region).

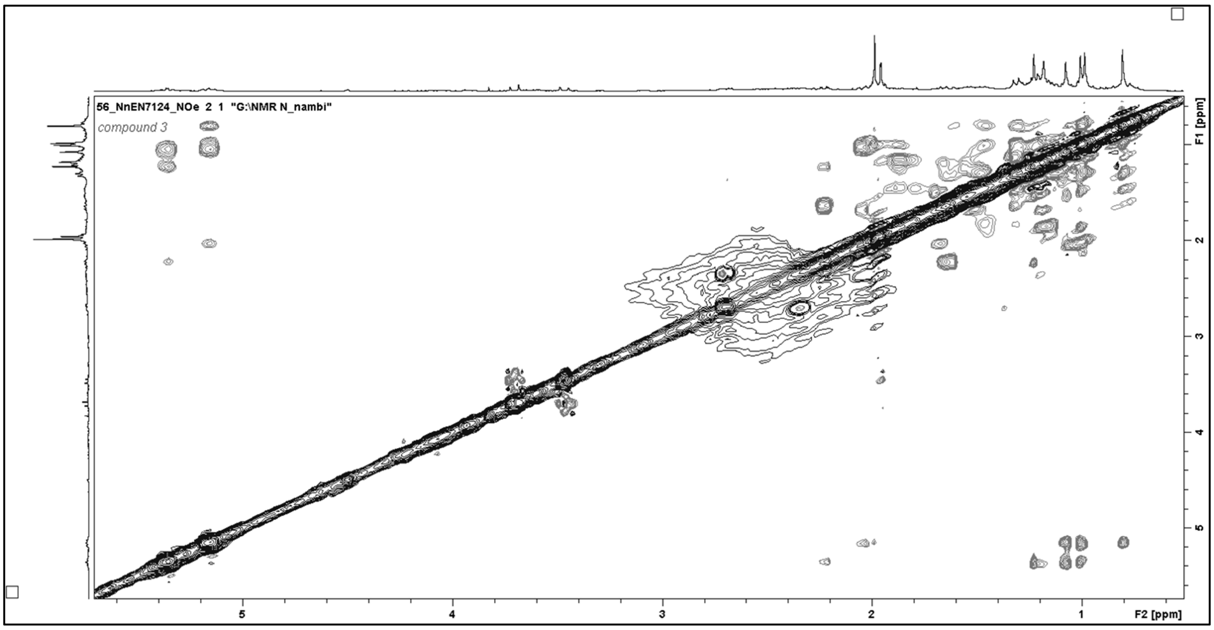

Figure S38: ^{1}H>^{1}H NOESY NMR spectrum of compound **3** in $CDCl_3$ (expanded view of the 0.0–3.0 ppm region).

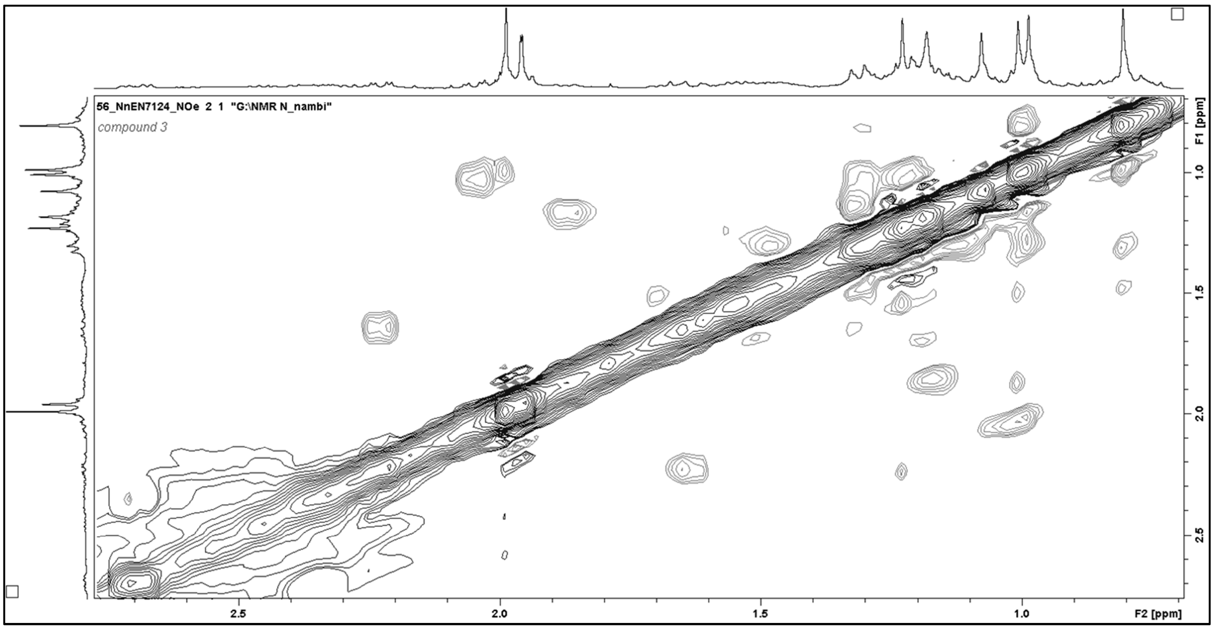

Figure S39: IR spectrum of compound **4**.

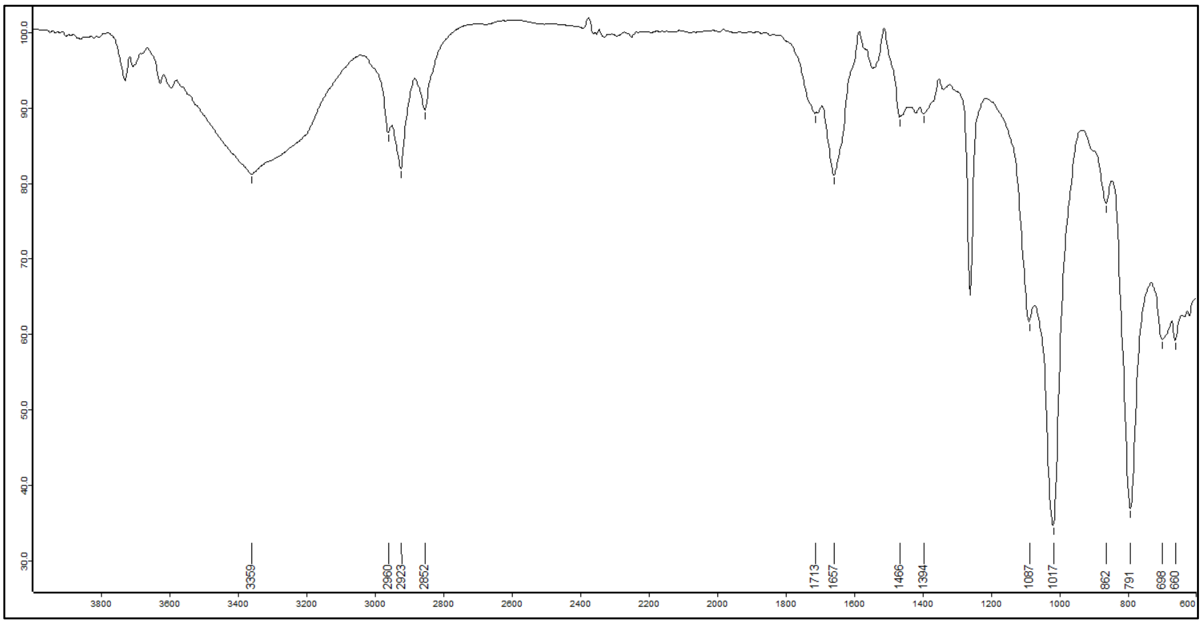

Figure S40: UV spectrum of **4** in methanol.

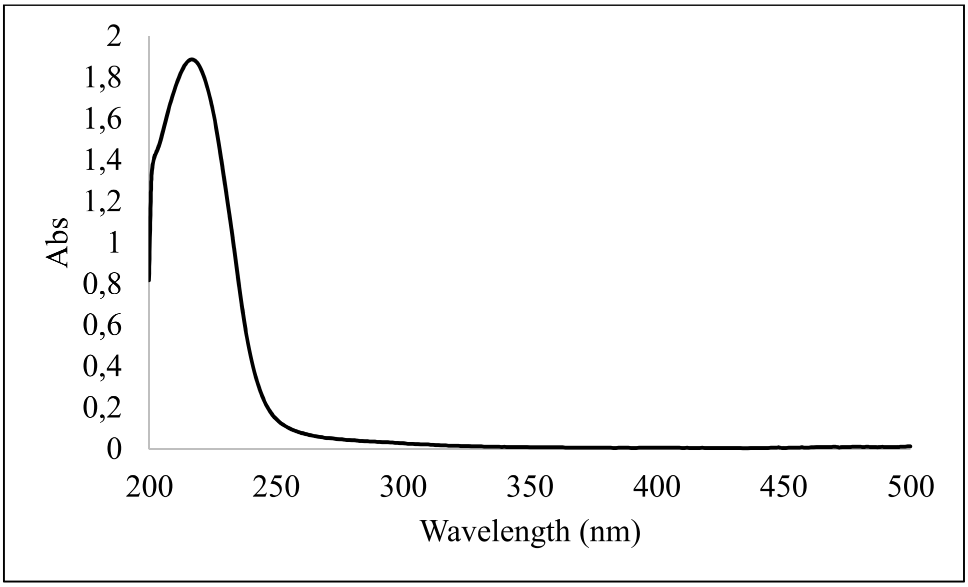

Figure S41: HRESIMS spectrogram of compound **4**.

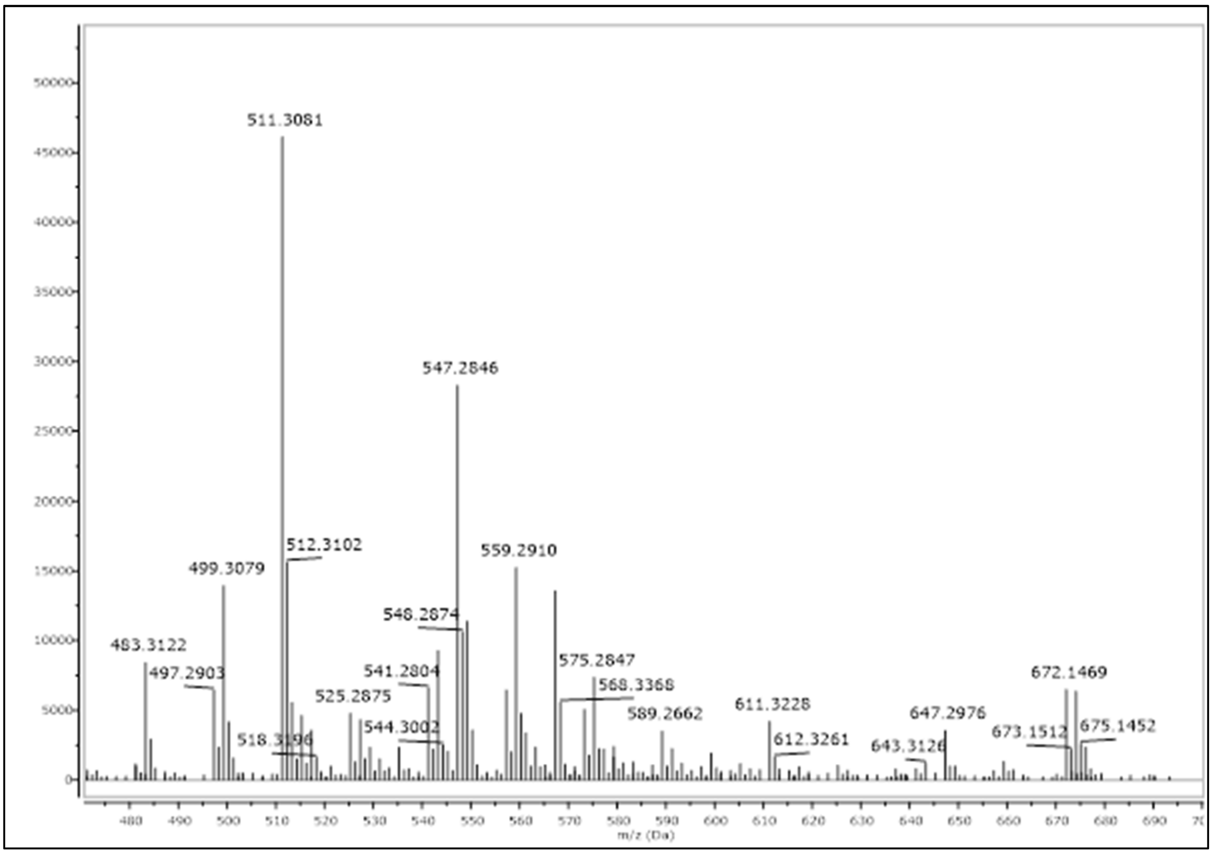

Figure S42: ^{1}H NMR spectrum of compound **4** in $CDCl_3$ + 2 drops of CD_3OD.

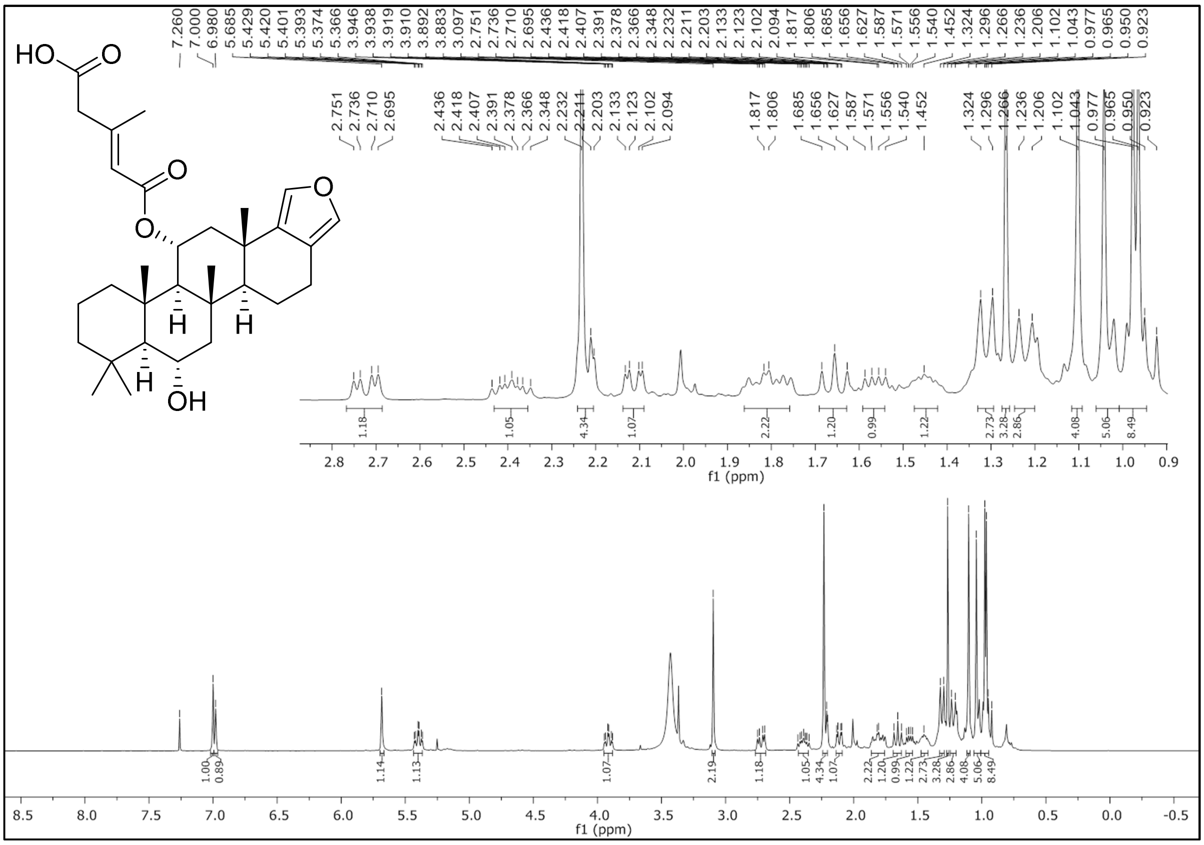

Figure S43: ^{13}C NMR spectrum of compound **4** in $CDCl_3$ + 2 drops of CD_3OD.

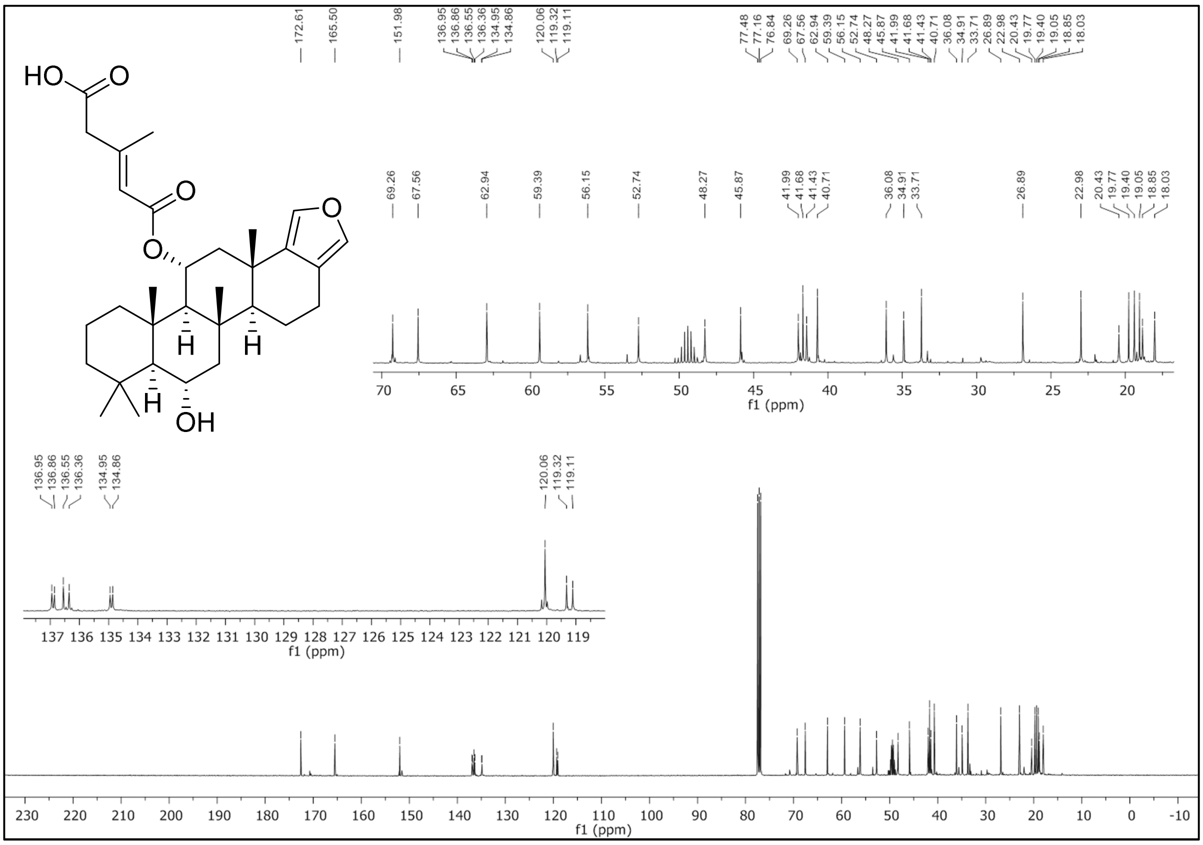

Figure S44: DEPTQ spectrum of compound **4** in $CDCl_3$ + 2 drops of CD_3OD.

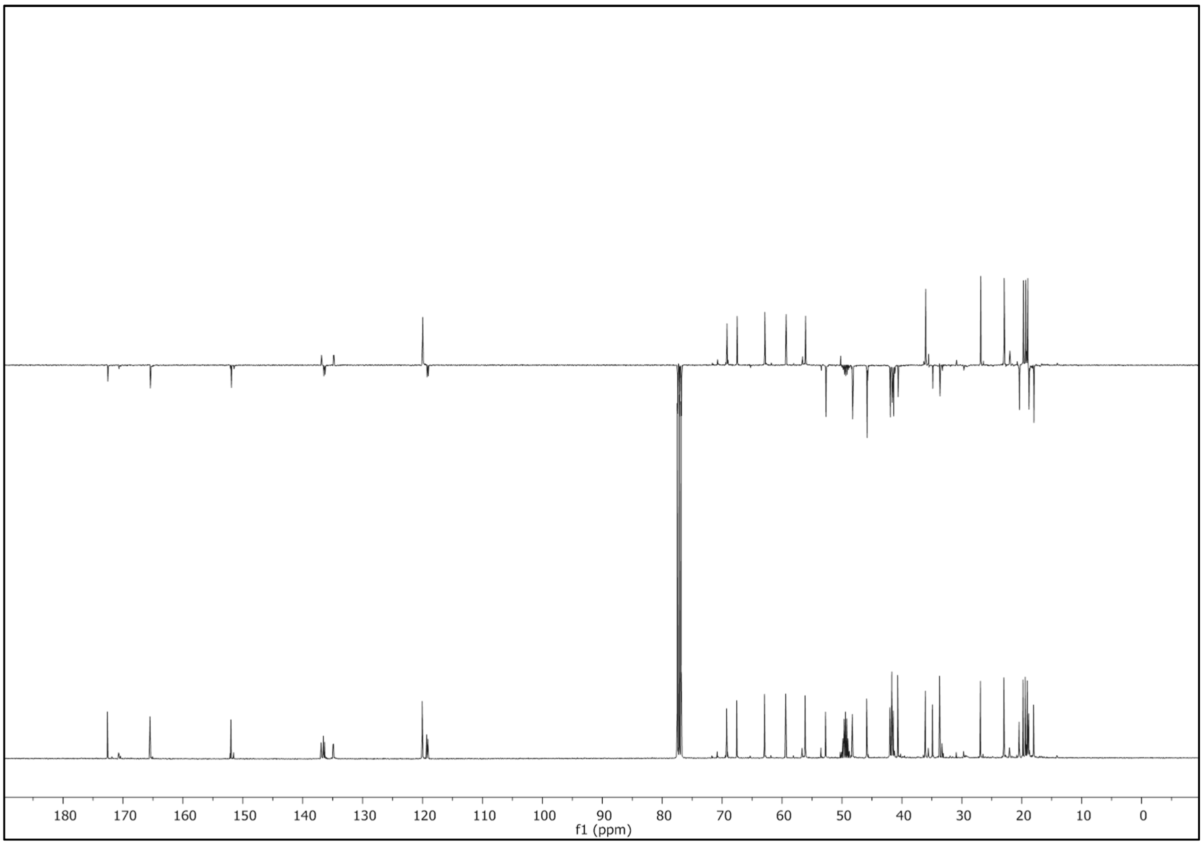

Figure S45: ^{1}H>^{1}H COSY spectrum of compound **4** in $CDCl_3$ + 2 drops of CD_3OD.

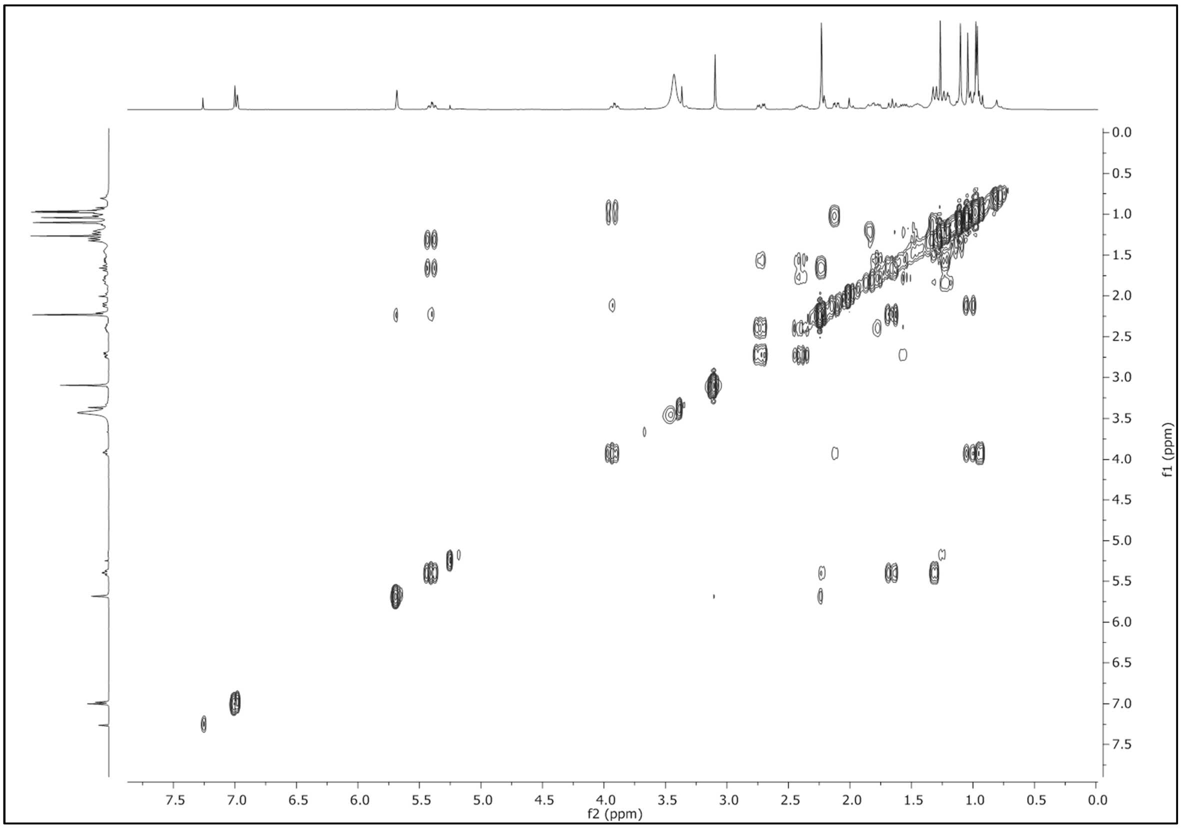

Figure S46: HSQC spectrum of compound **4** in $CDCl_3$ + 2 drops of CD_3OD.

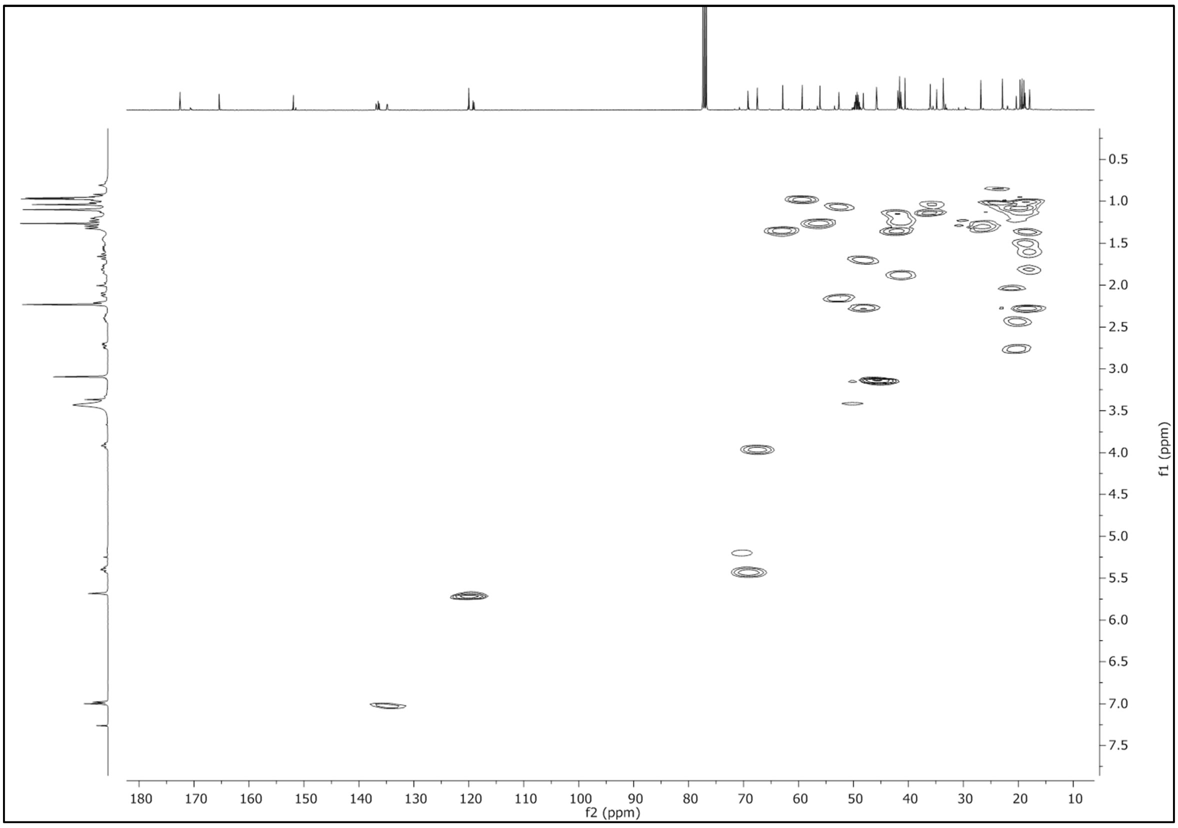

Figure S47: HMBC spectrum of compound **4** in $CDCl_3$ + 2 drops of CD_3OD.

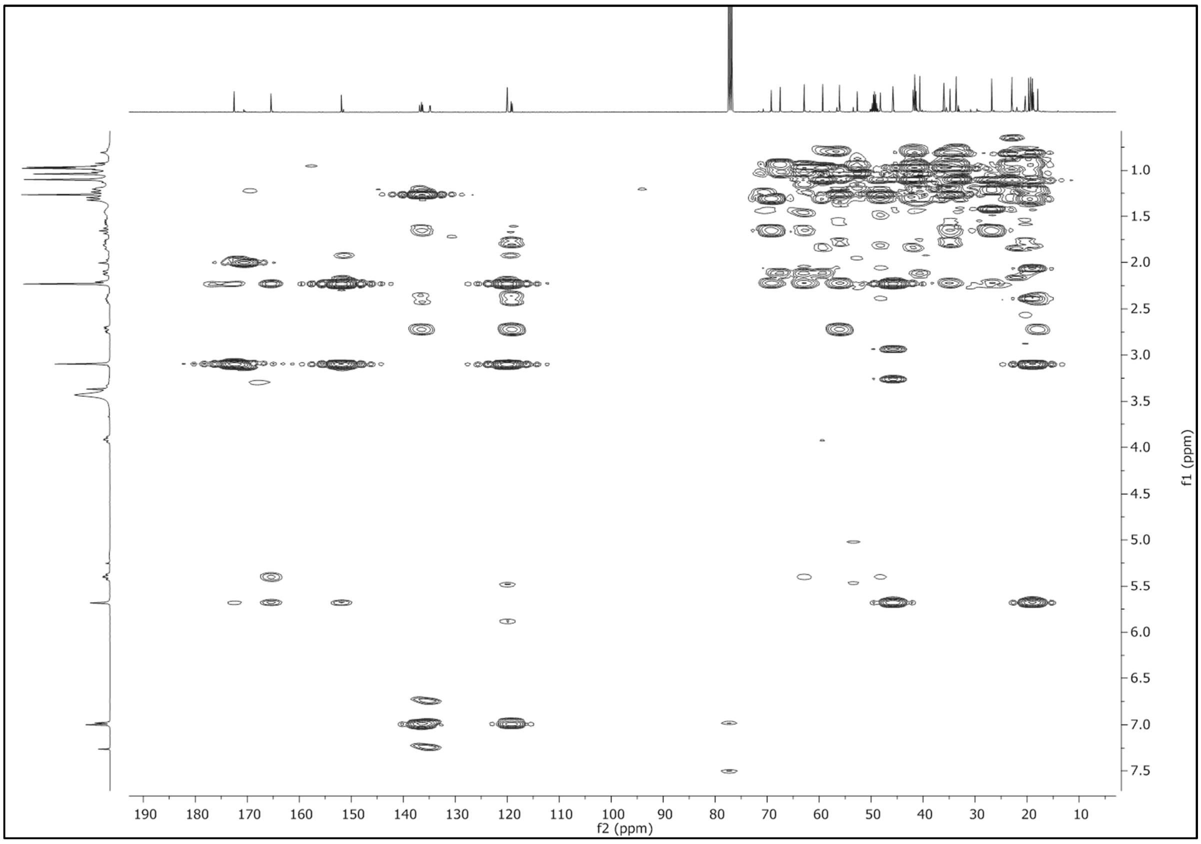

Figure S48: ^{1}H–^{1}H NOESY spectrum of compound **4** in $CDCl_3$ + 2 drops of CD_3OD (overview).

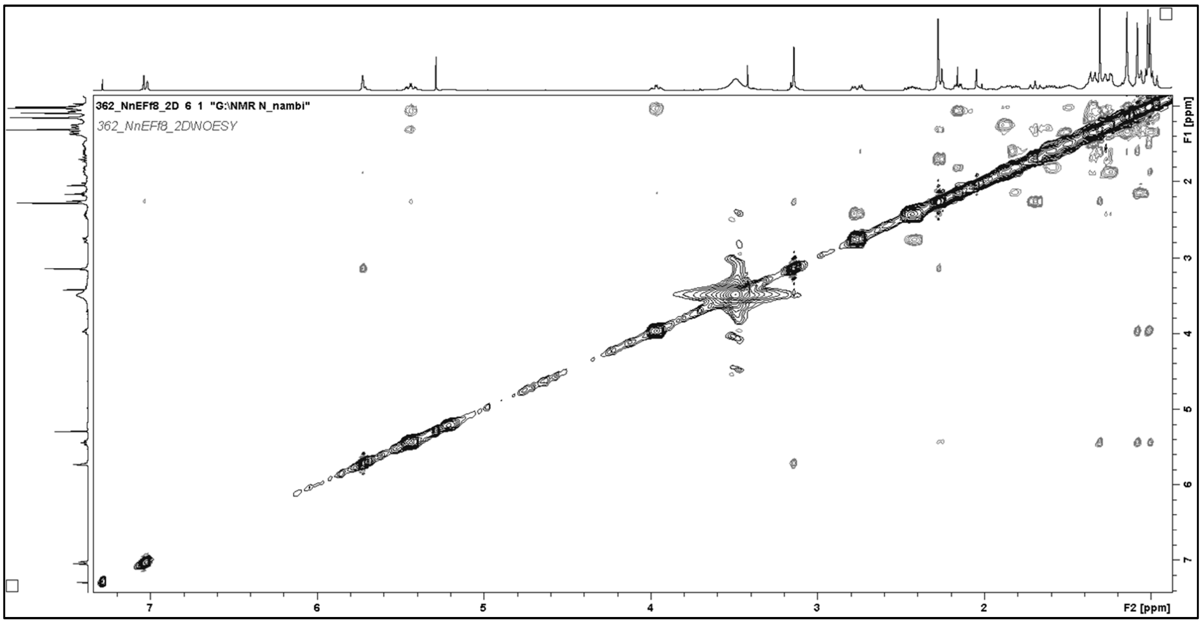

Figure S49: ^{1}H–^{1}H NOESY NMR spectrum of compound **4** in $CDCl_3$ + 2 drops of CD_3OD (expanded view of the 0.0–6.0 ppm region).

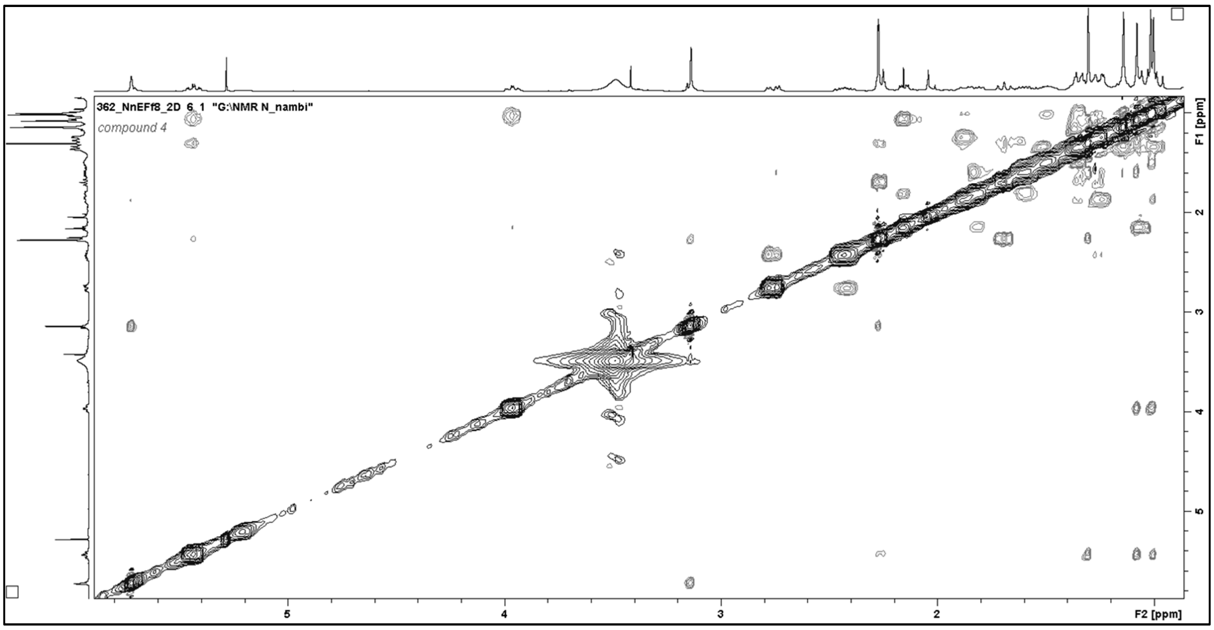

Figure S50: ^{1}H-^{1}H NOESY NMR spectrum of compound **4** in $CDCl_3$ + 2 drops of CD_3OD (expanded view of the 0.0–3.0 ppm region).

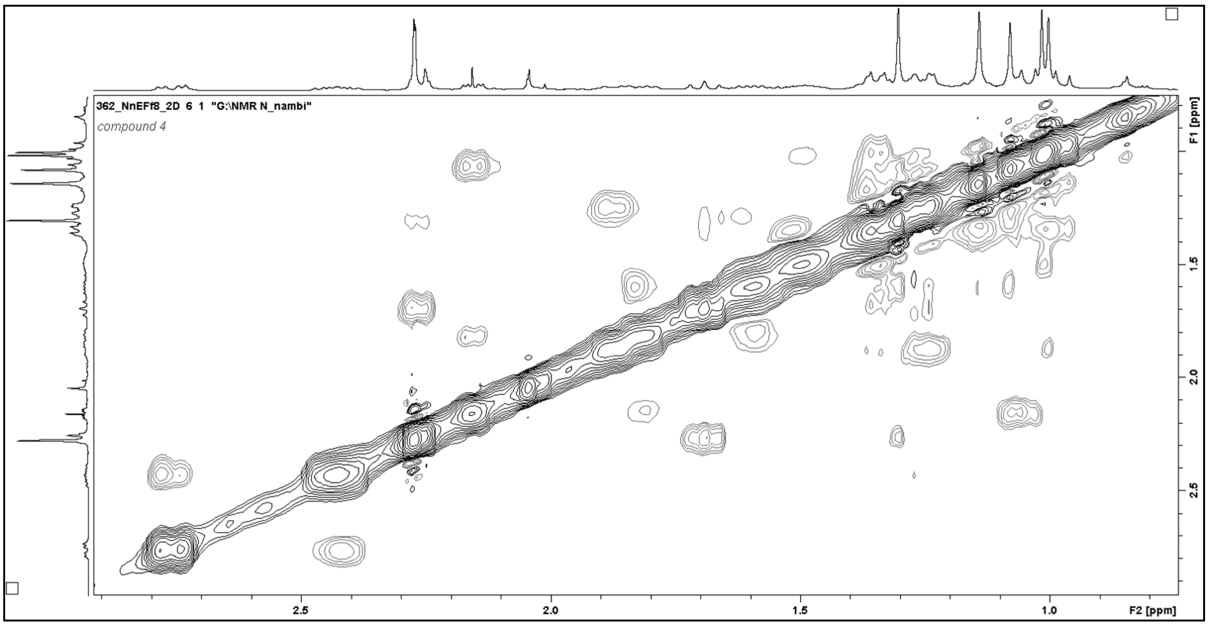

Figure S51: ^{1}H NMR spectrum of compound **4** in CD_3CN.

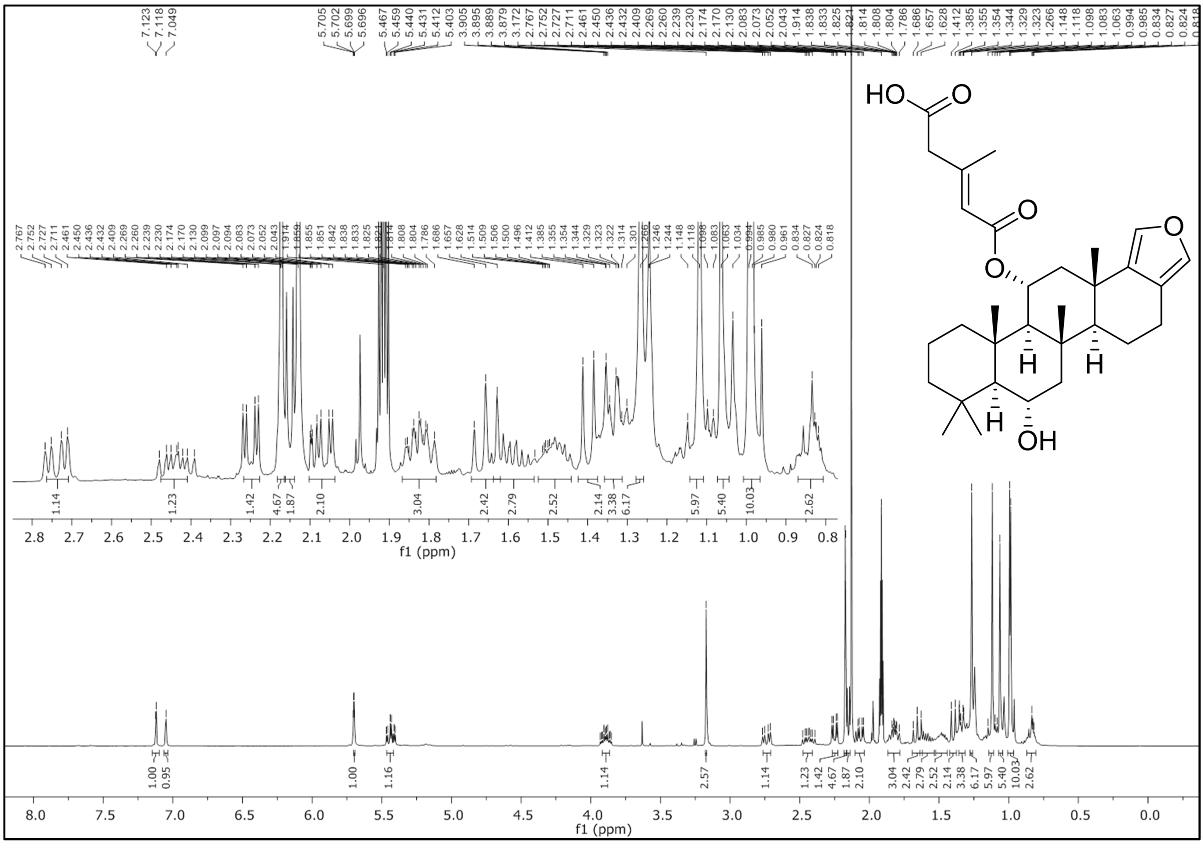

Figure S52: ^{13}C NMR spectrum of compound **4** in CD_3CN.

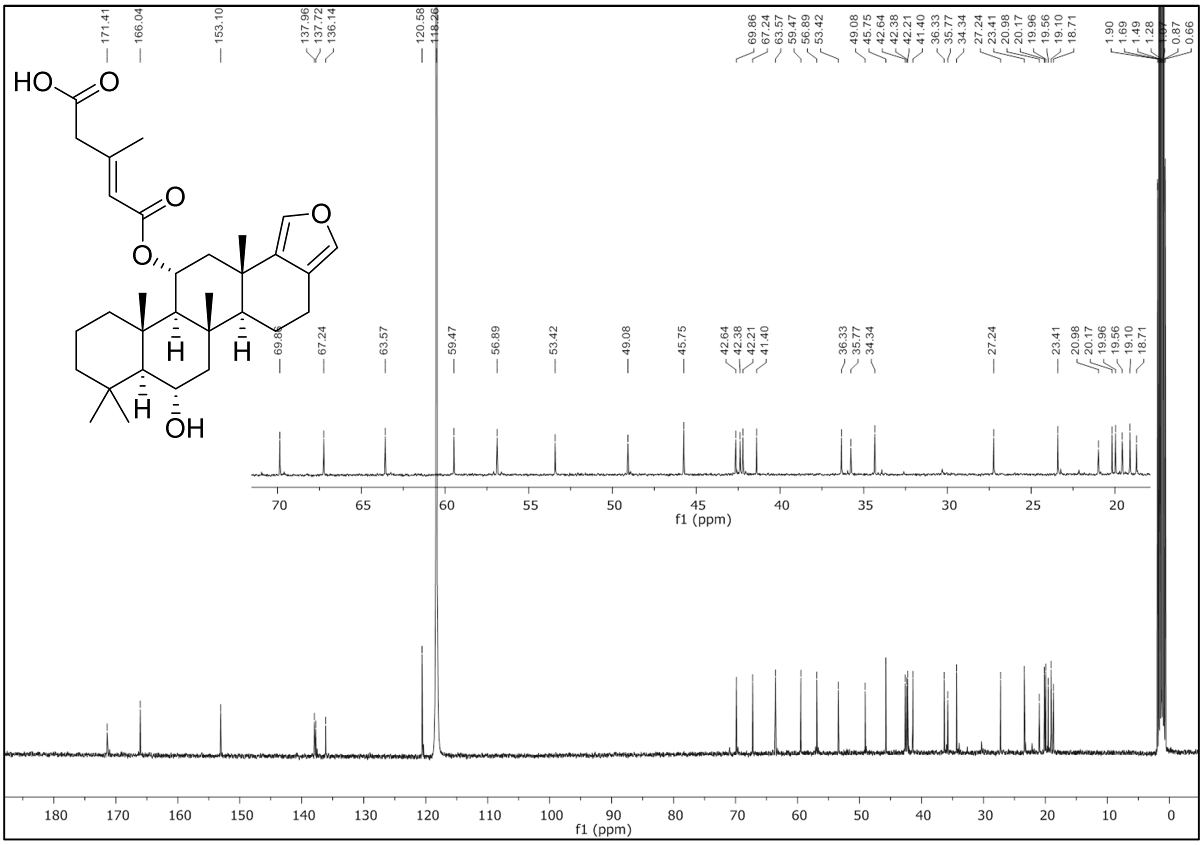

Figure S53: IR spectrum of compound **5**.

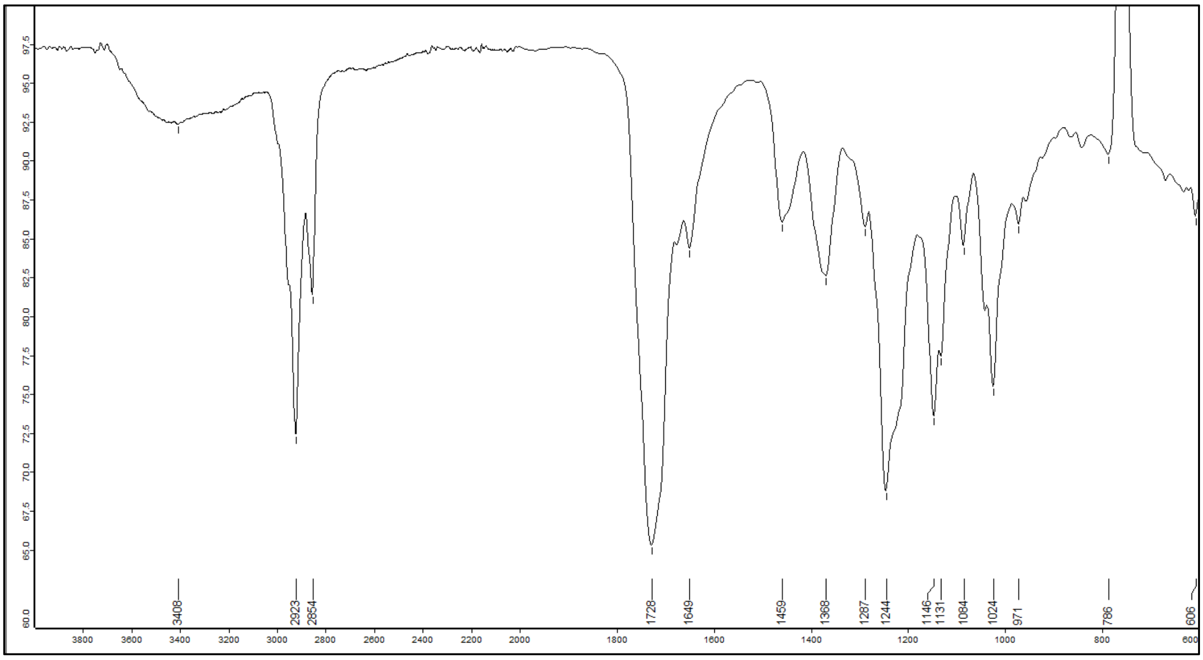

Figure S54: UV spectrum of **5** in methanol.

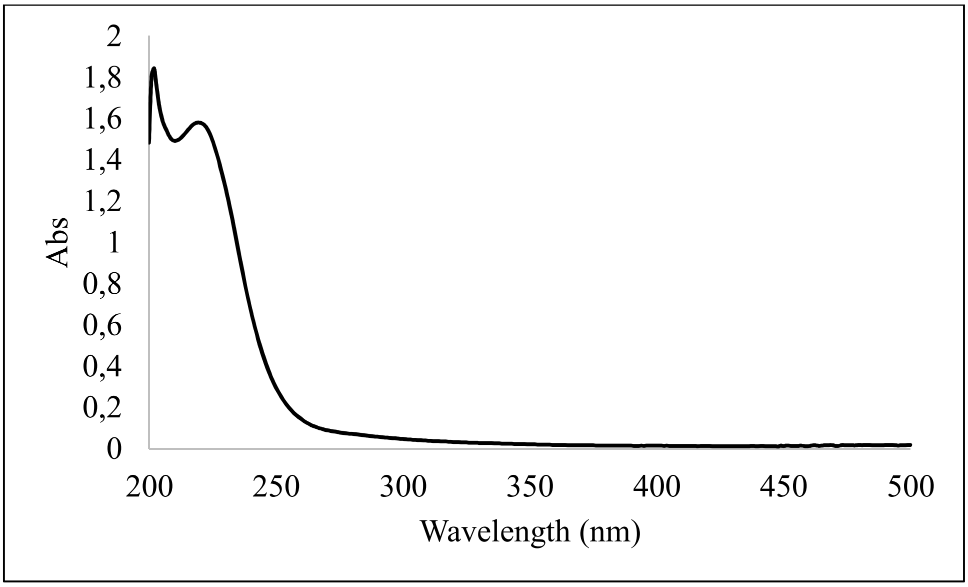

Figure S55: HRESIMS spectrogram of compound **5**.

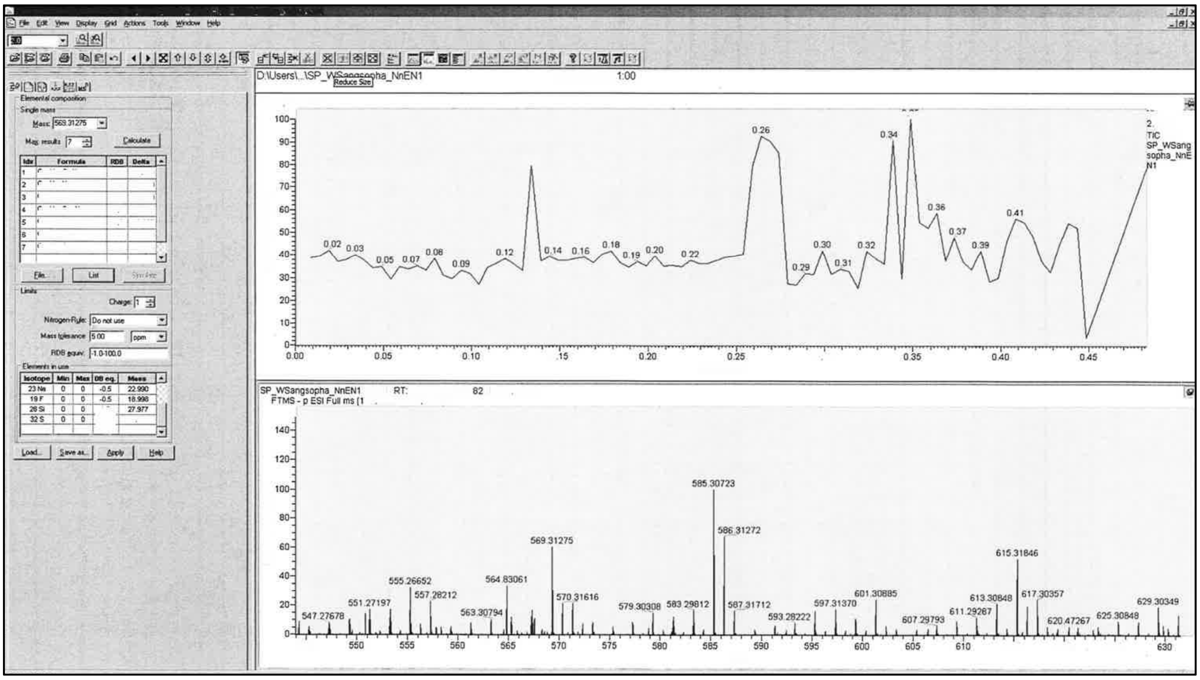

Figure S56: ^{1}H NMR spectrum of compound **5** in $CDCl_3$.

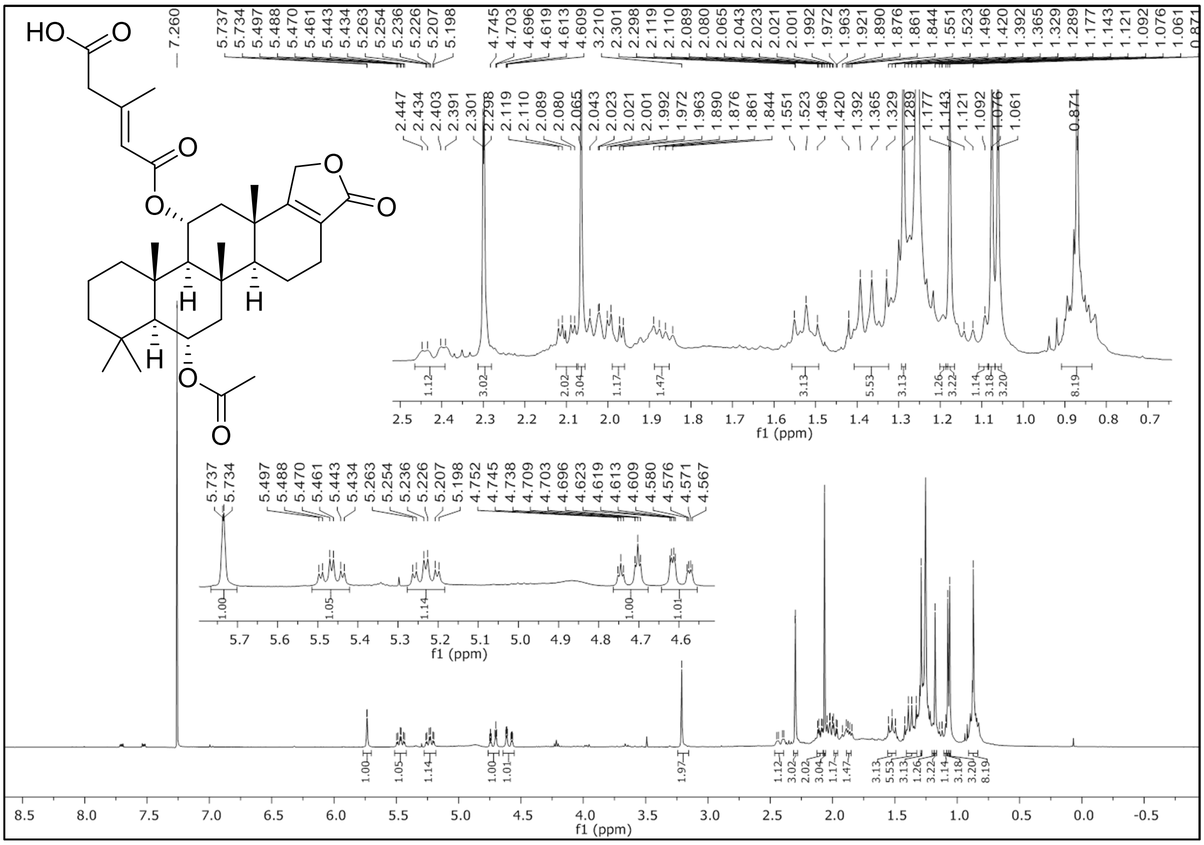

Figure S57: ^{13}C NMR spectrum of compound **5** in $CDCl_3$.

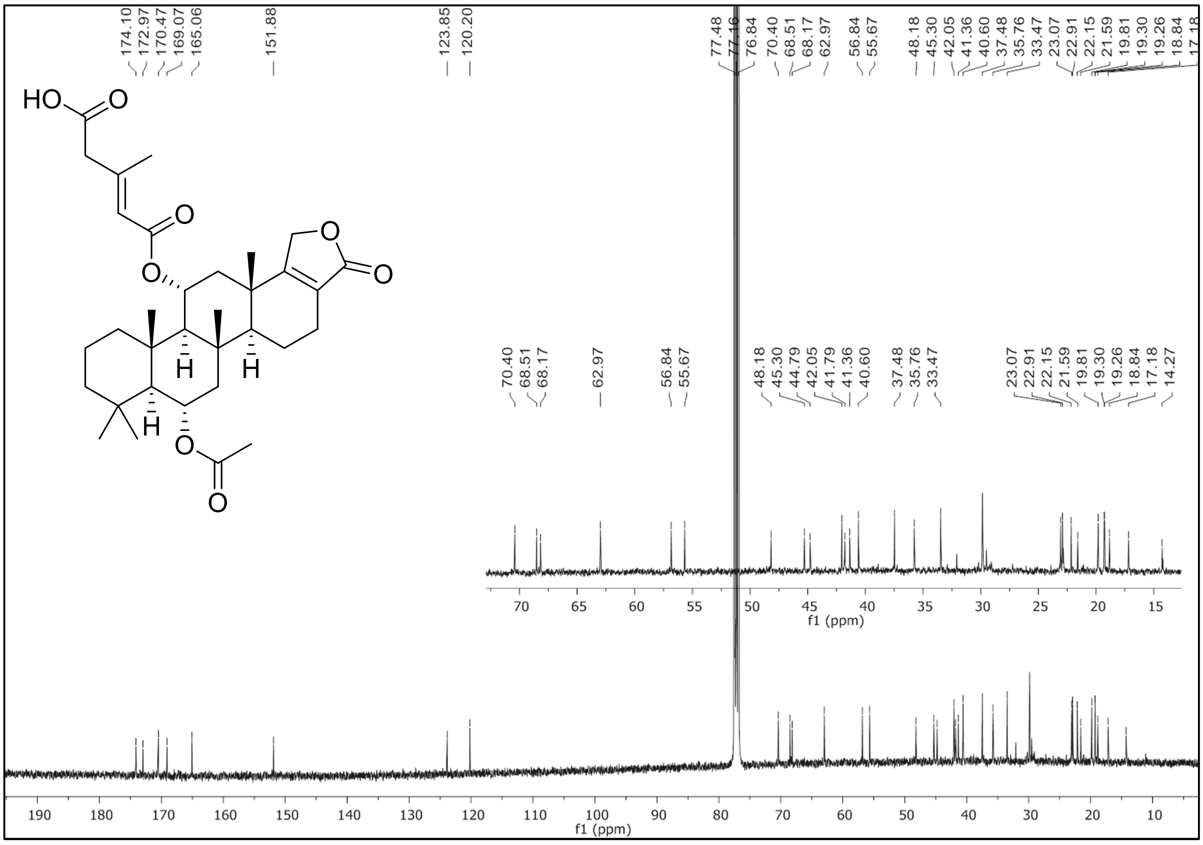

Figure S58: DEPTQ spectrum of compound **5** in $CDCl_3$.

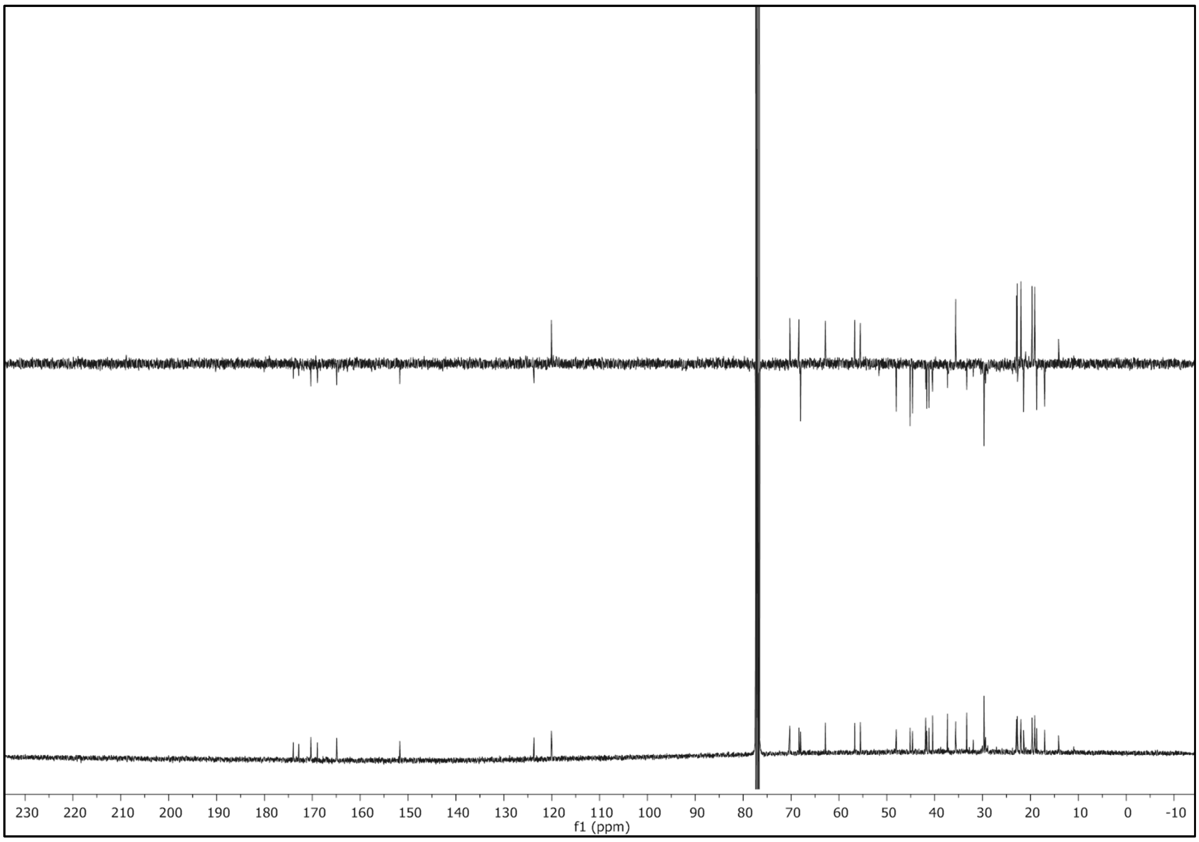

Figure S59: ^{1}H>^{1}H COSY NMR spectrum of compound **5** in $CDCl_3$.

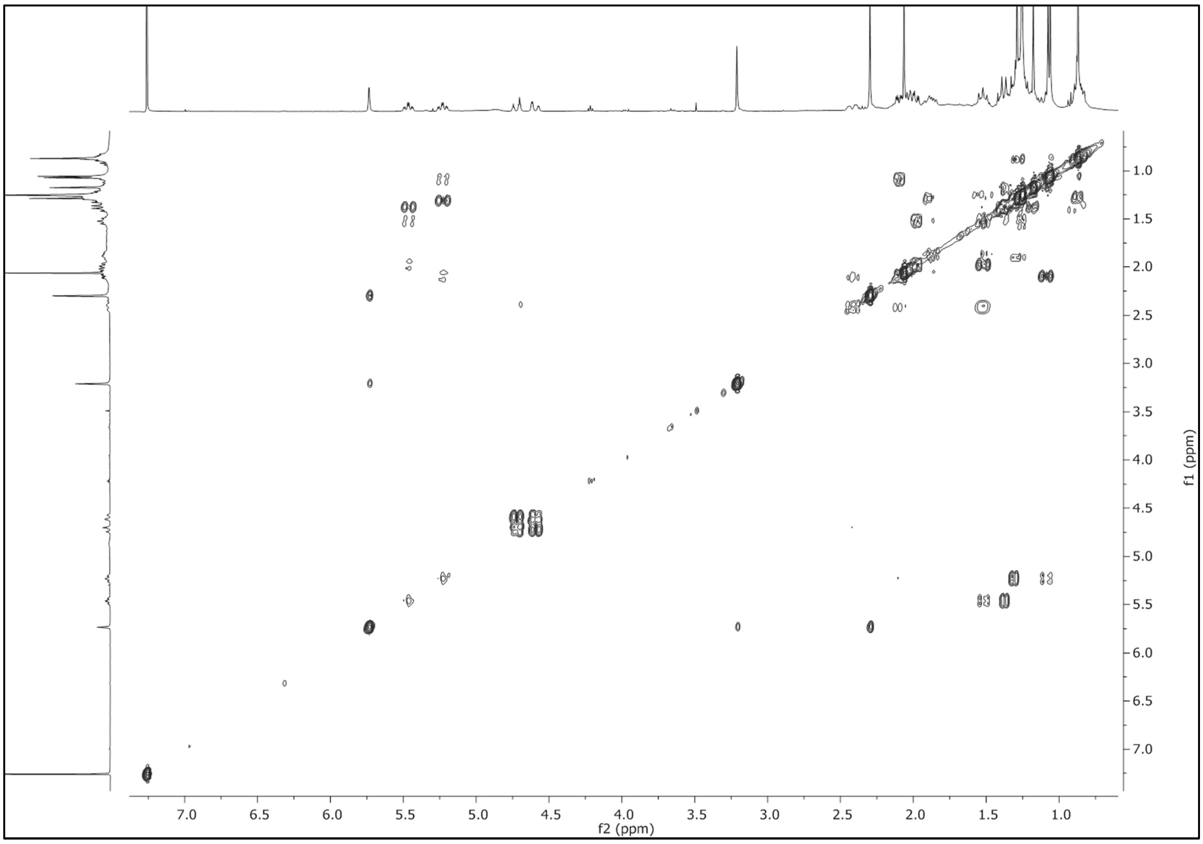

Figure S60: HSQC spectrum of compound **5** in $CDCl_3$.

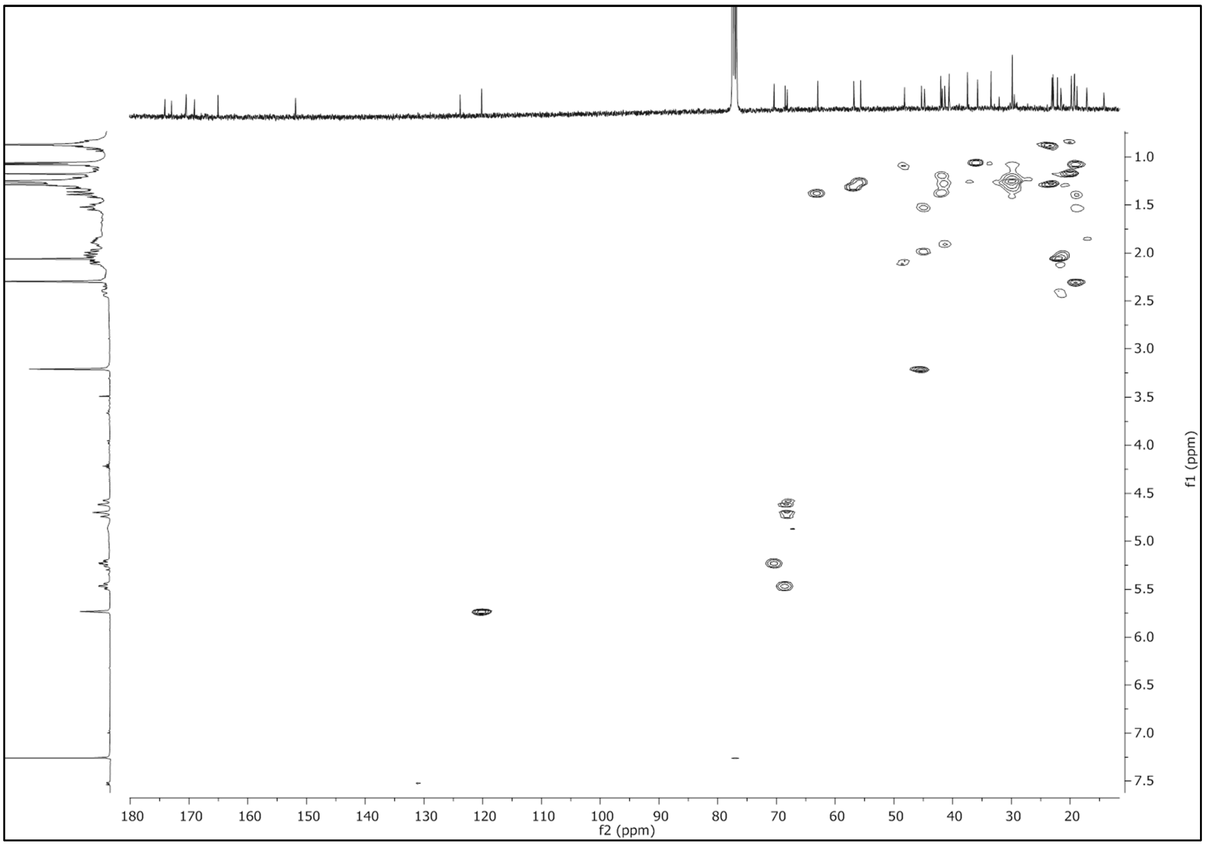

Figure S61: HMBC spectrum of compound **5** in $CDCl_3$.

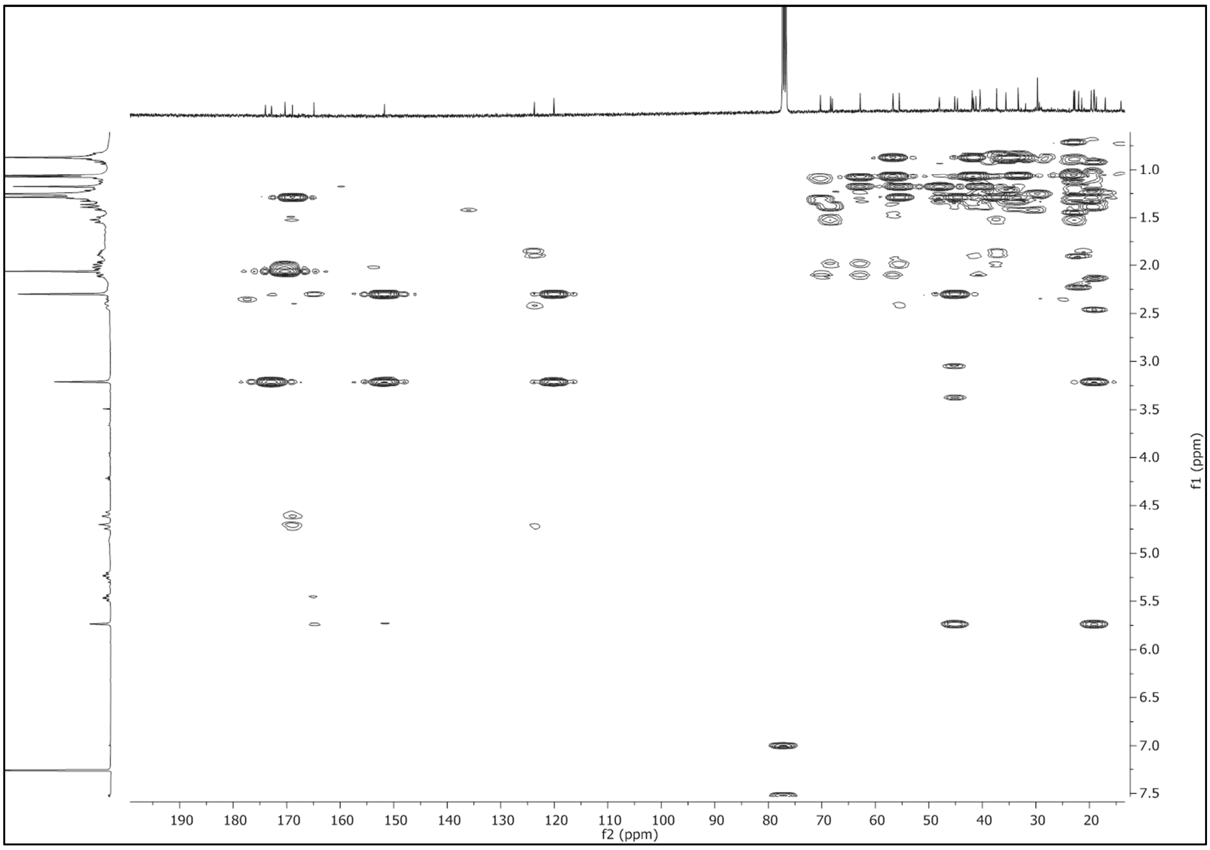

Figure S62: ^{1}H–^{1}H NOESY spectrum of compound **5** in $CDCl_3$ (overview).

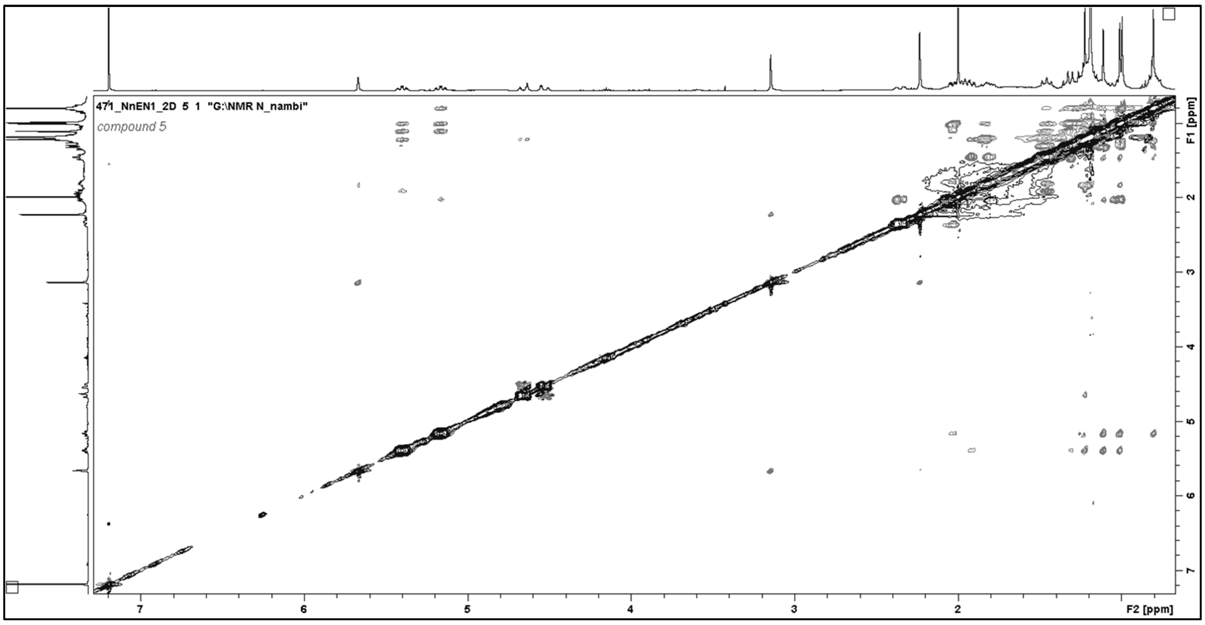

Figure S63: ^{1}H–^{1}H NOESY NMR spectrum of compound **5** in $CDCl_3$ (expanded view of the 4.3–5.7 × 0.6–2.5 ppm region).

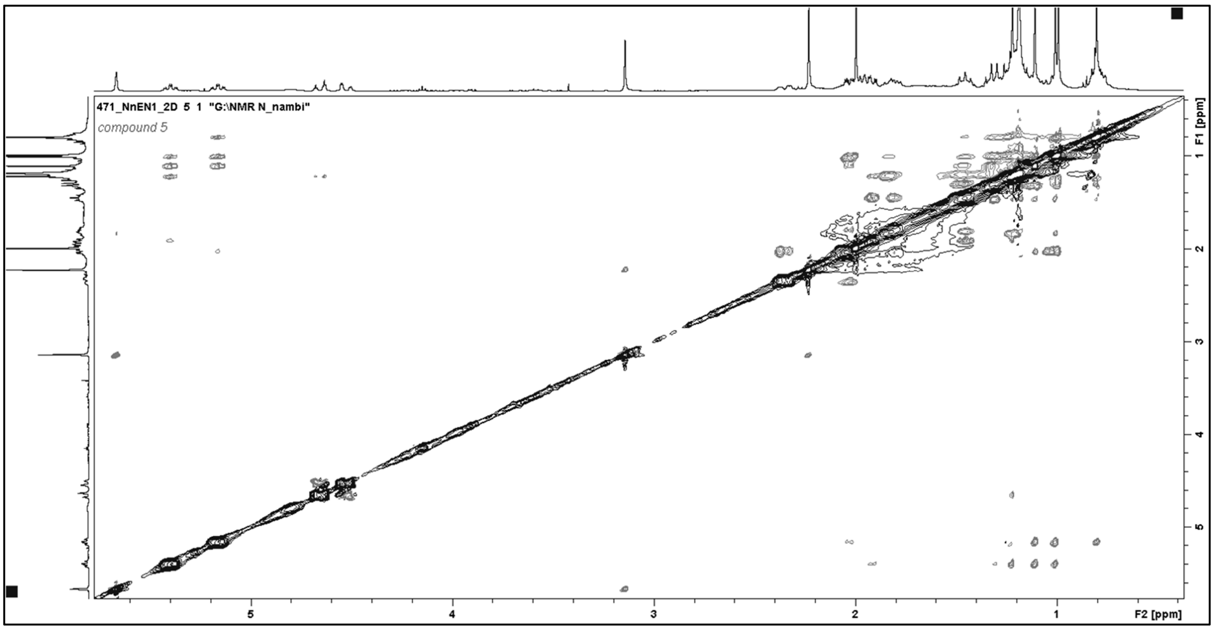

Figure S64: ^{1}H–^{1}H NOESY NMR spectrum of compound **5** in $CDCl_3$ (expanded view of the 0.0–3.0 ppm region).

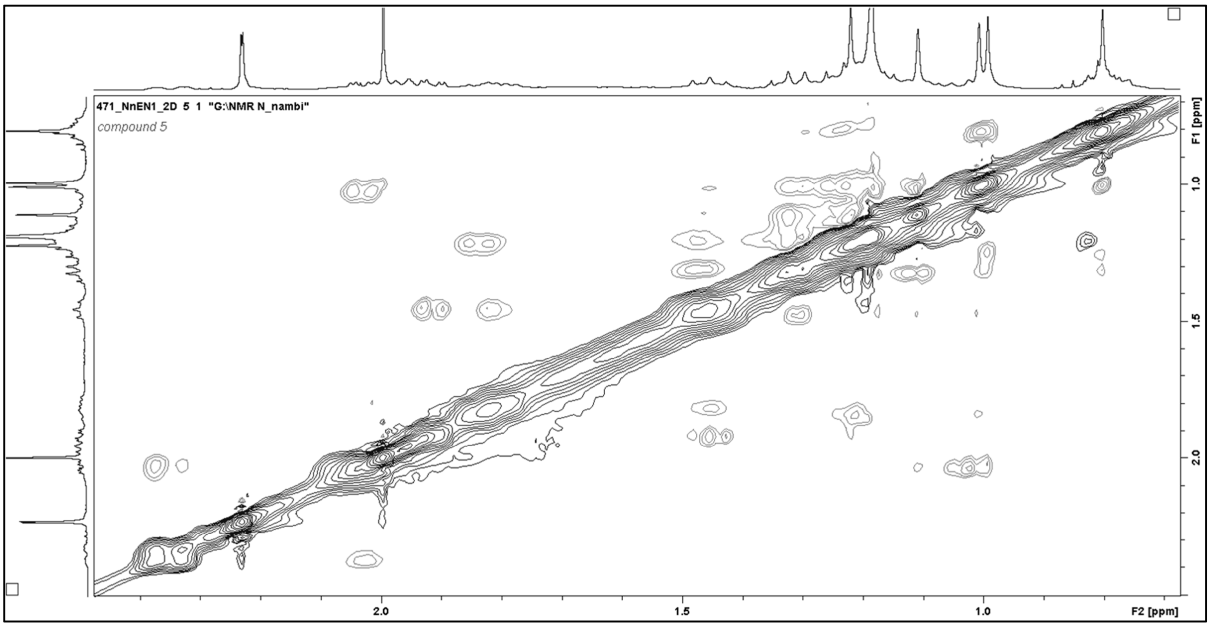

Figure S65: IR spectrum of compound **6**.

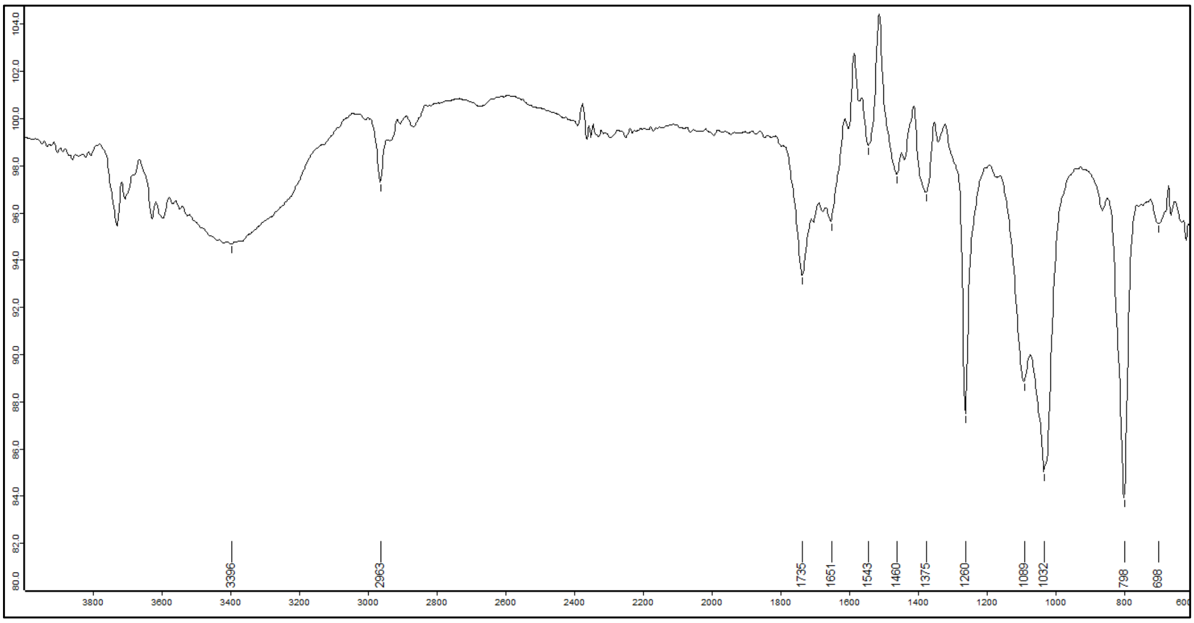

Figure S66: UV spectrum of **6** in methanol.

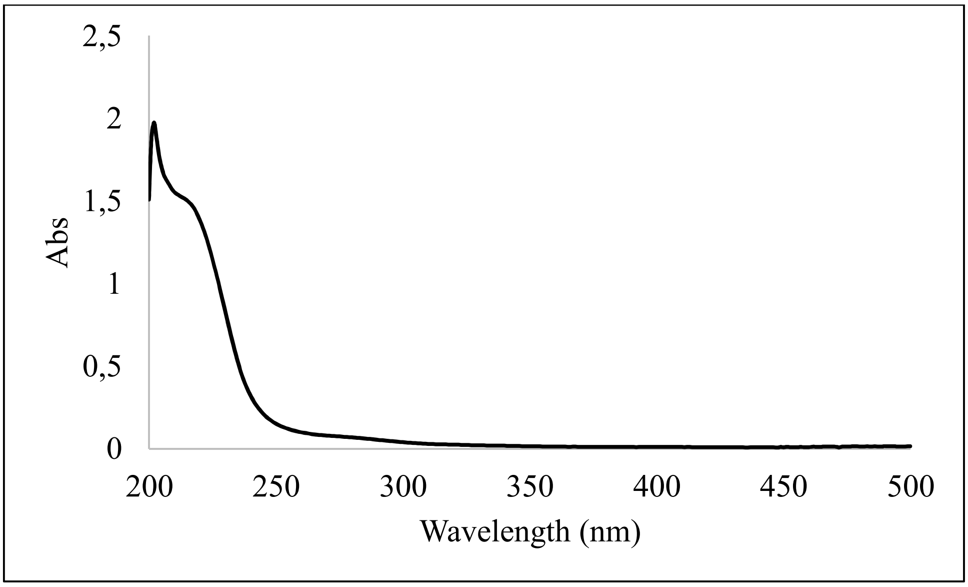

Figure S67: HRESIMS spectrogram of compound **6**.

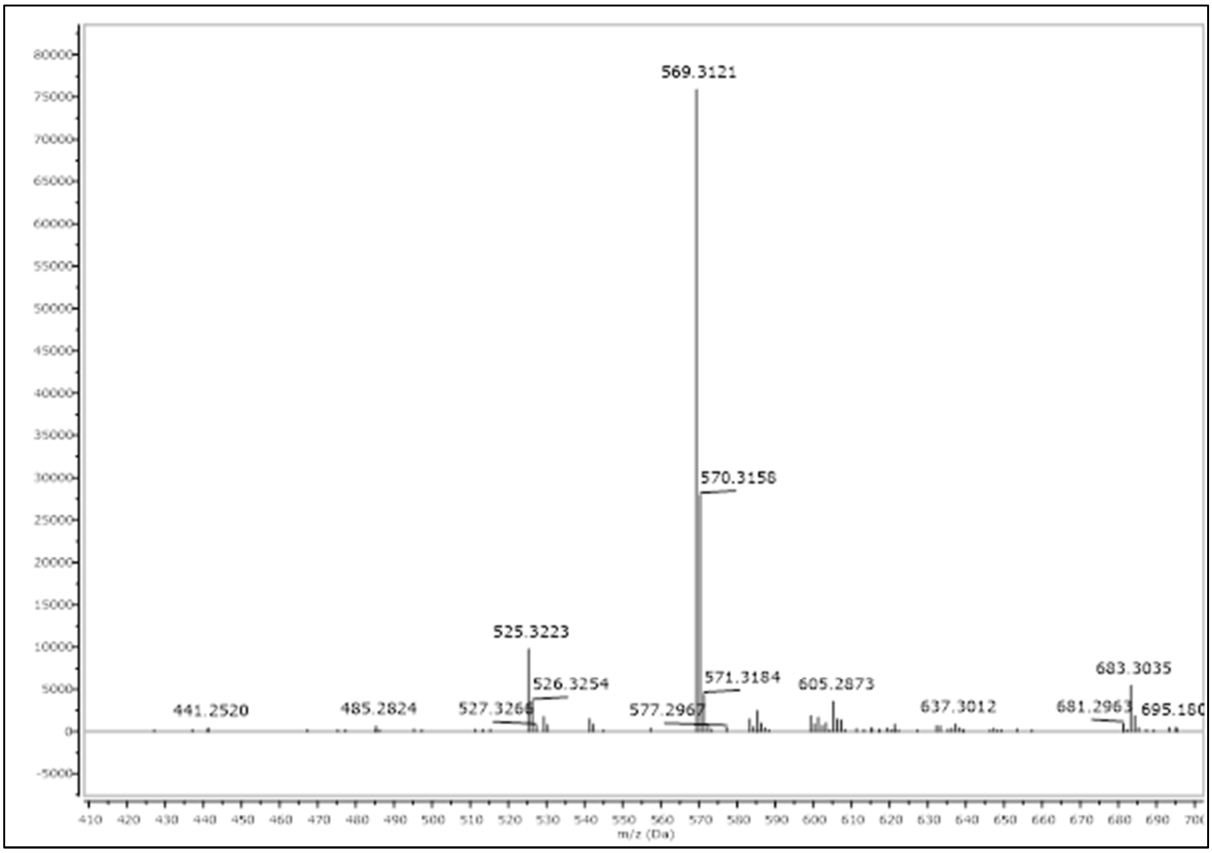

Figure S68: ^{1}H NMR spectrum of compound **6** in $CDCl_3$.

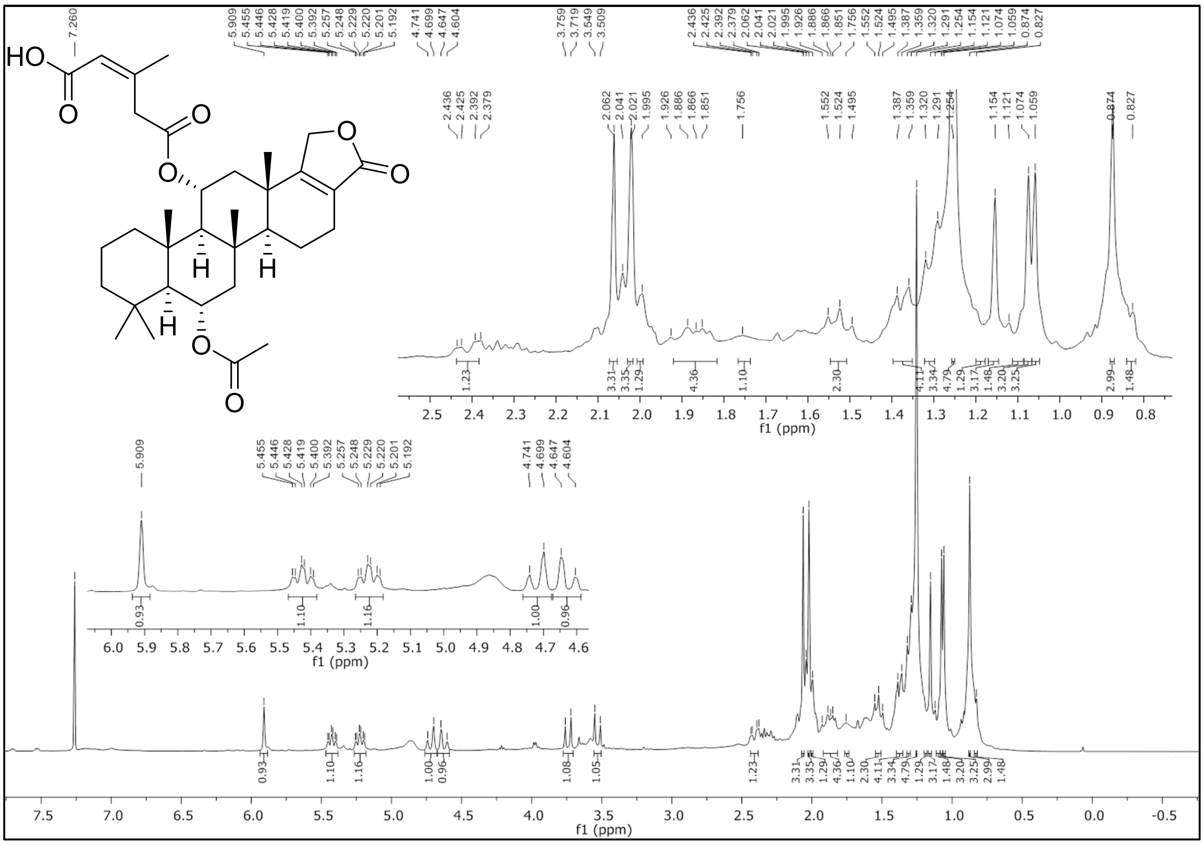

Figure S69: ^{13}C NMR spectrum of compound **6** in $CDCl_3$.

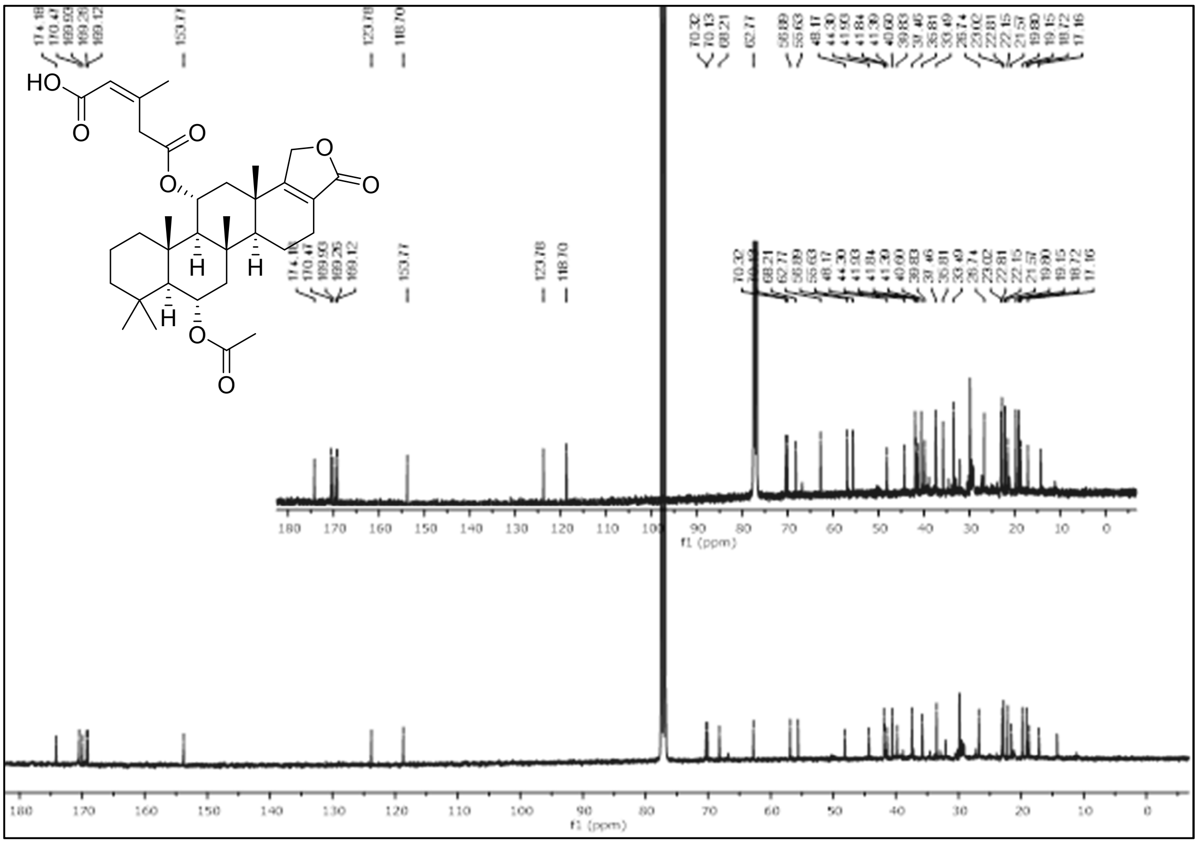

Figure S70: DEPTQ spectrum of compound **6** in $CDCl_3$.

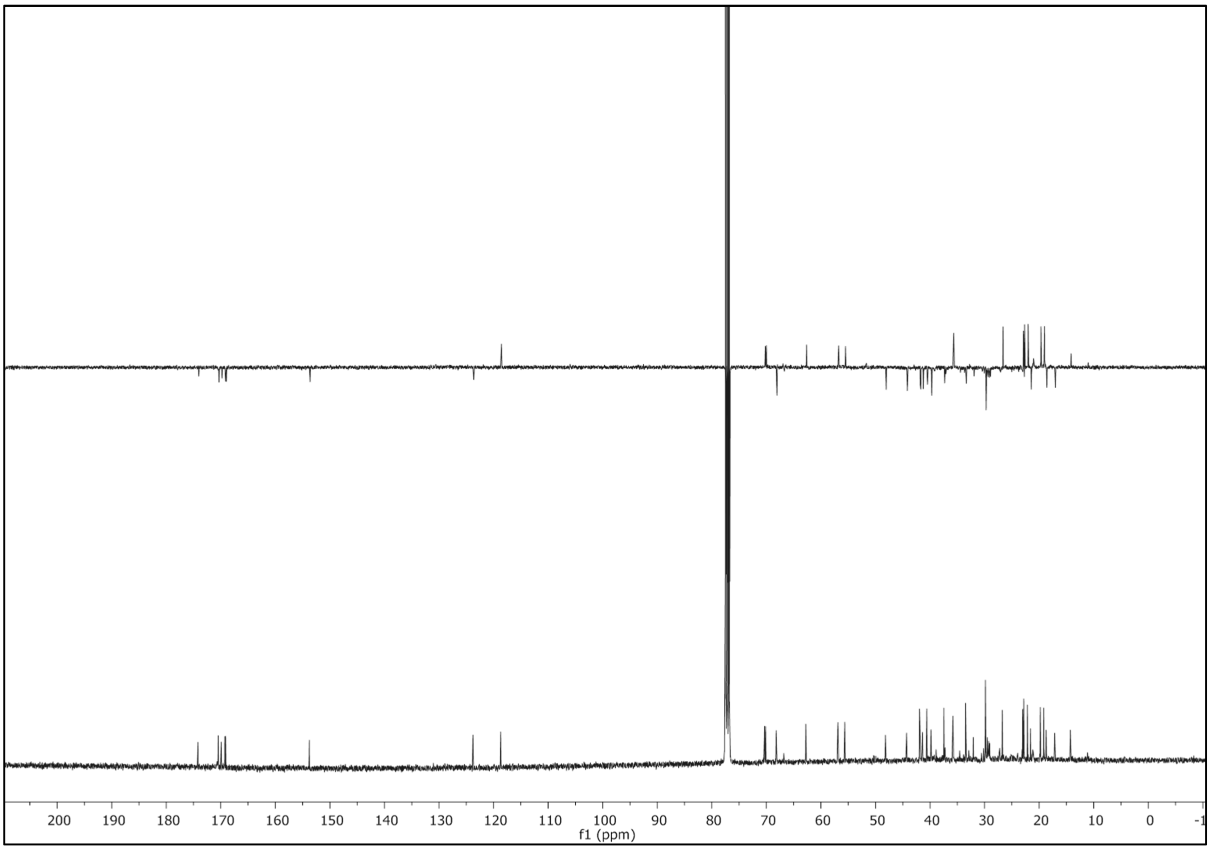

Figure S71: ^{1}H-^{1}H COSY NMR spectrum of compound **6** in $CDCl_3$.

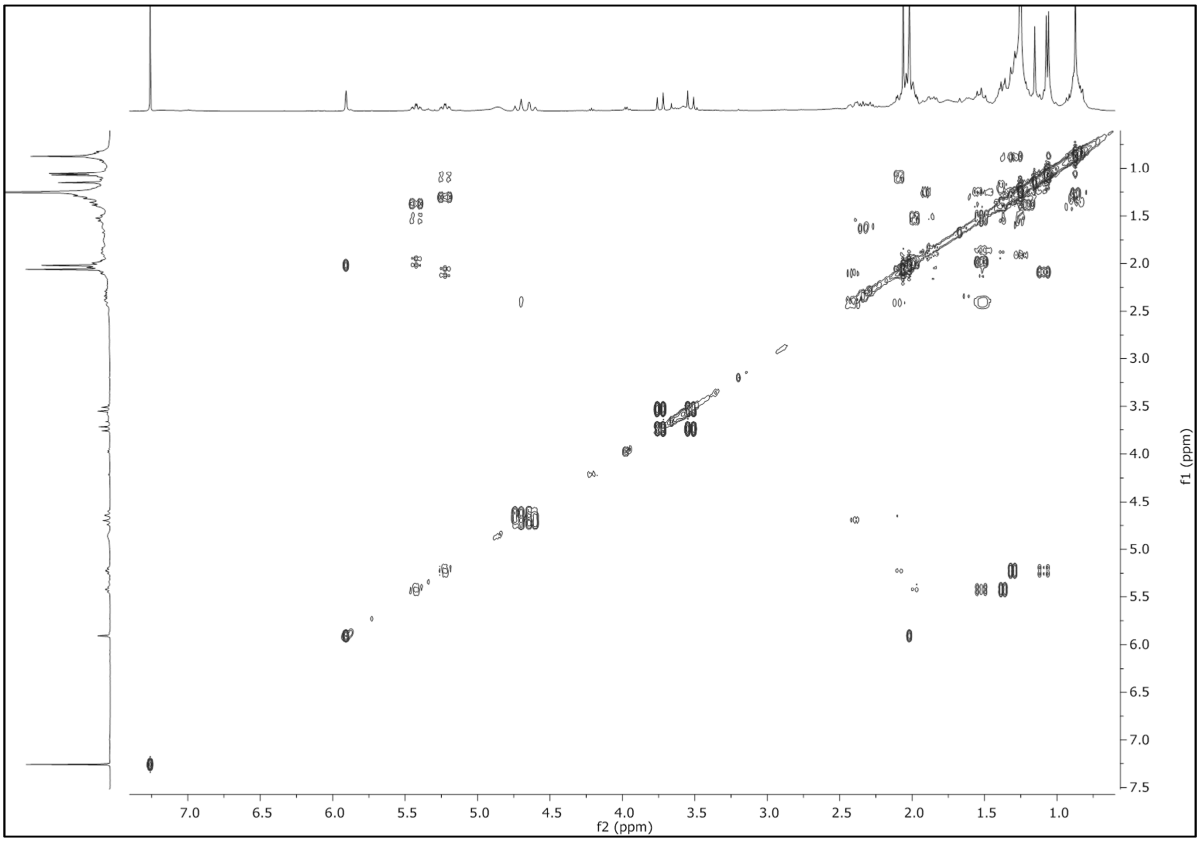

Figure S72: HSQC spectrum of compound **6** in $CDCl_3$.

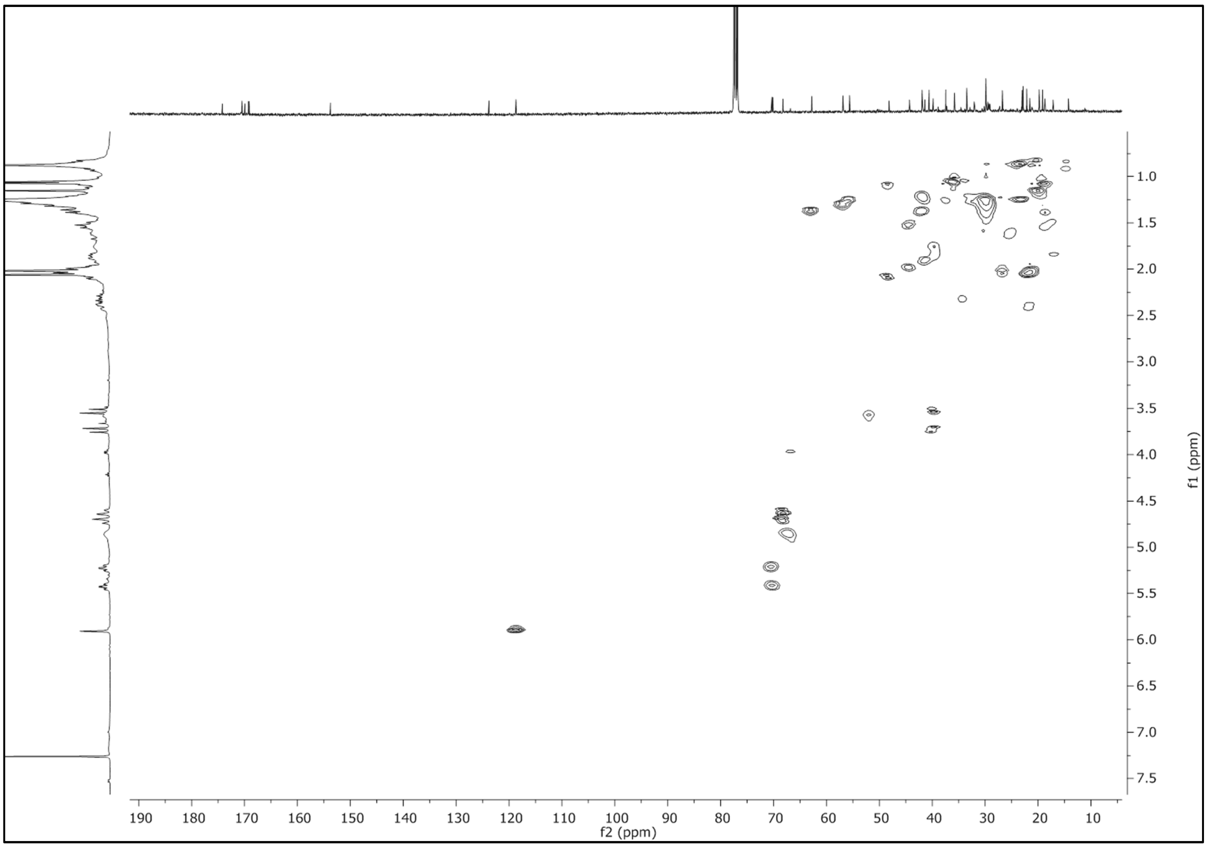

Figure S73: HMBC spectrum of compound **6** in $CDCl_3$.

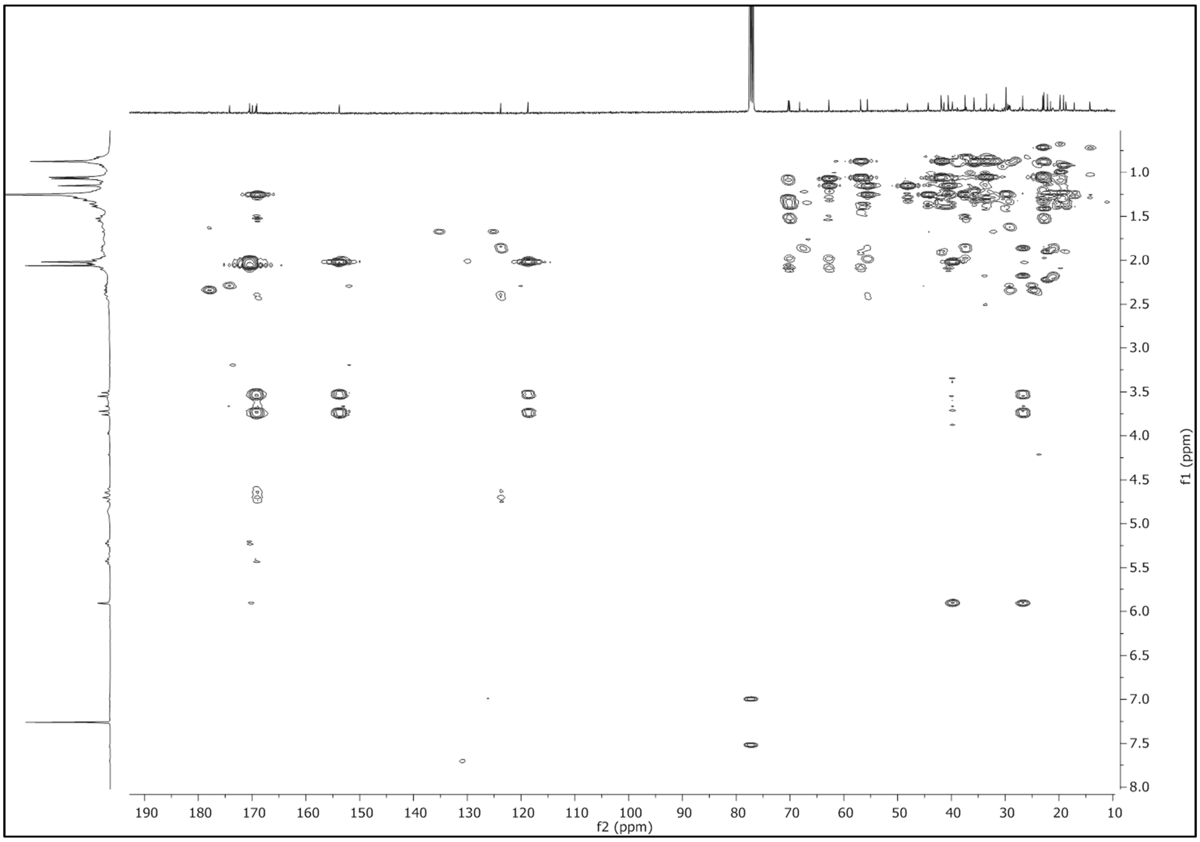

Figure S74: ^{1}H–^{1}H NOESY spectrum of compound **6** in $CDCl_3$ (overview).

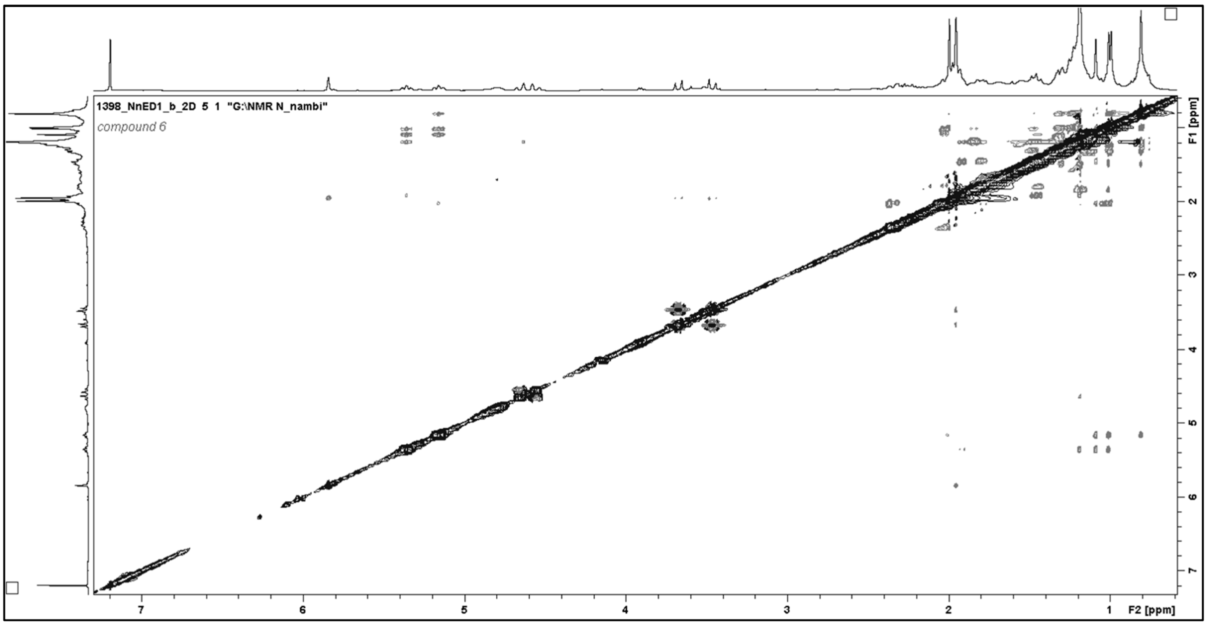

Figure S75: ^{1}H–^{1}H NOESY NMR spectrum of compound **6** in $CDCl_3$ (expanded view of the 0.0–6.0 ppm region).

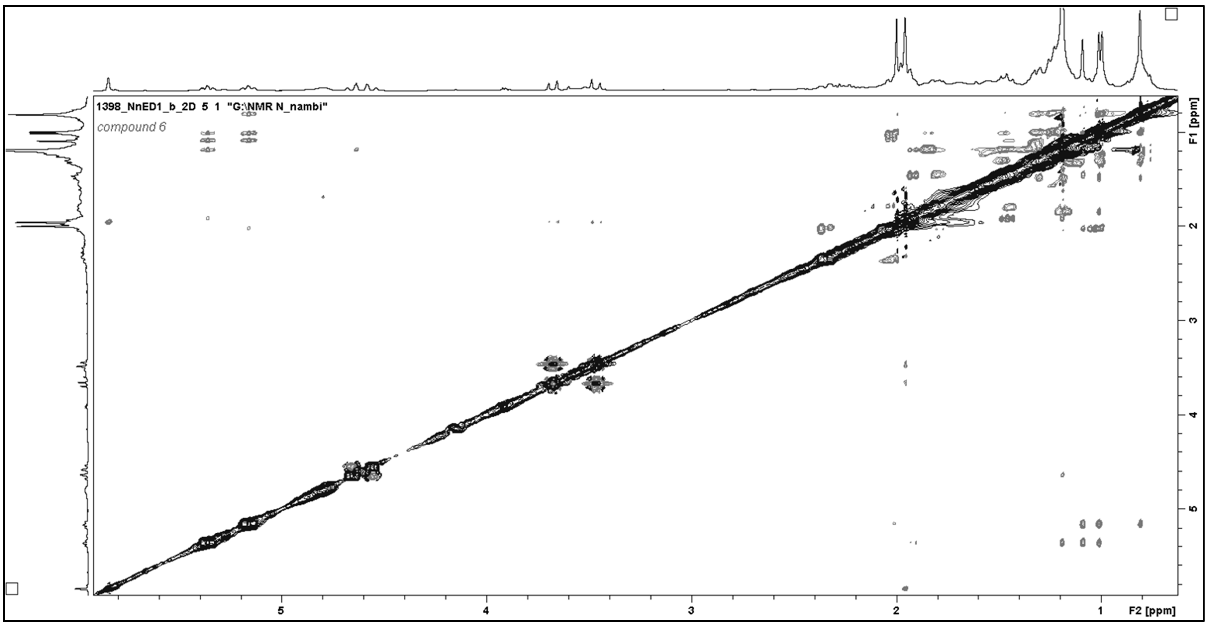

Figure S76: ^{1}H–^{1}H NOESY NMR spectrum of compound **6** in $CDCl_3$ (expanded view of the 0.7–2.6 ppm region).

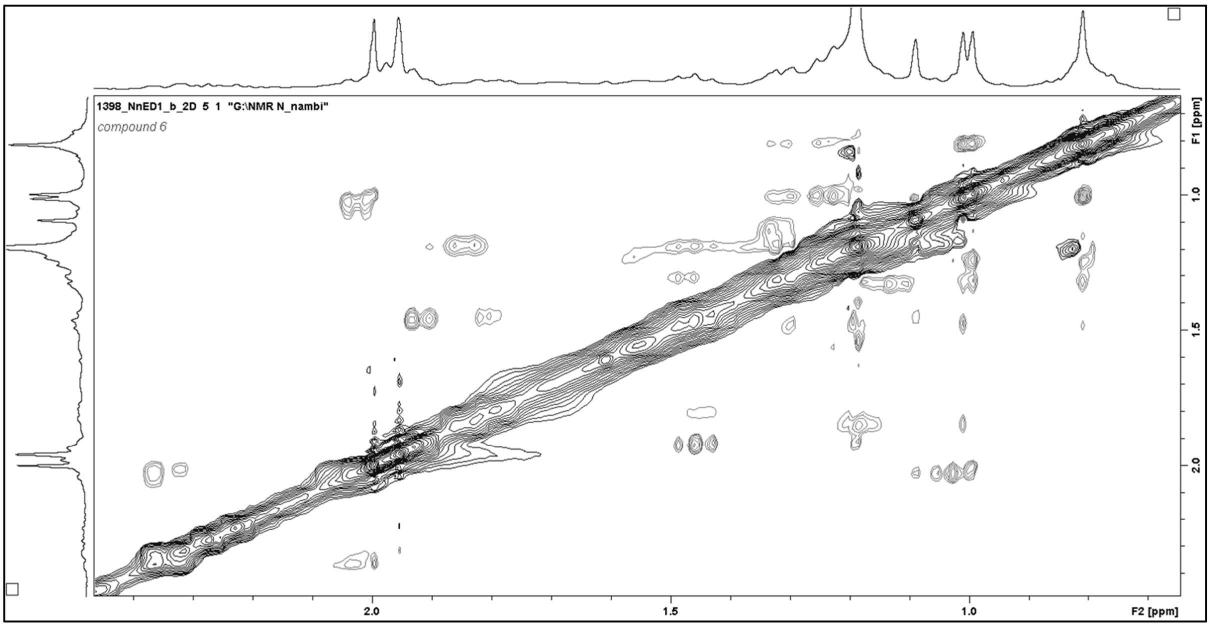

Figure S77: IR spectrum of compound **7**.

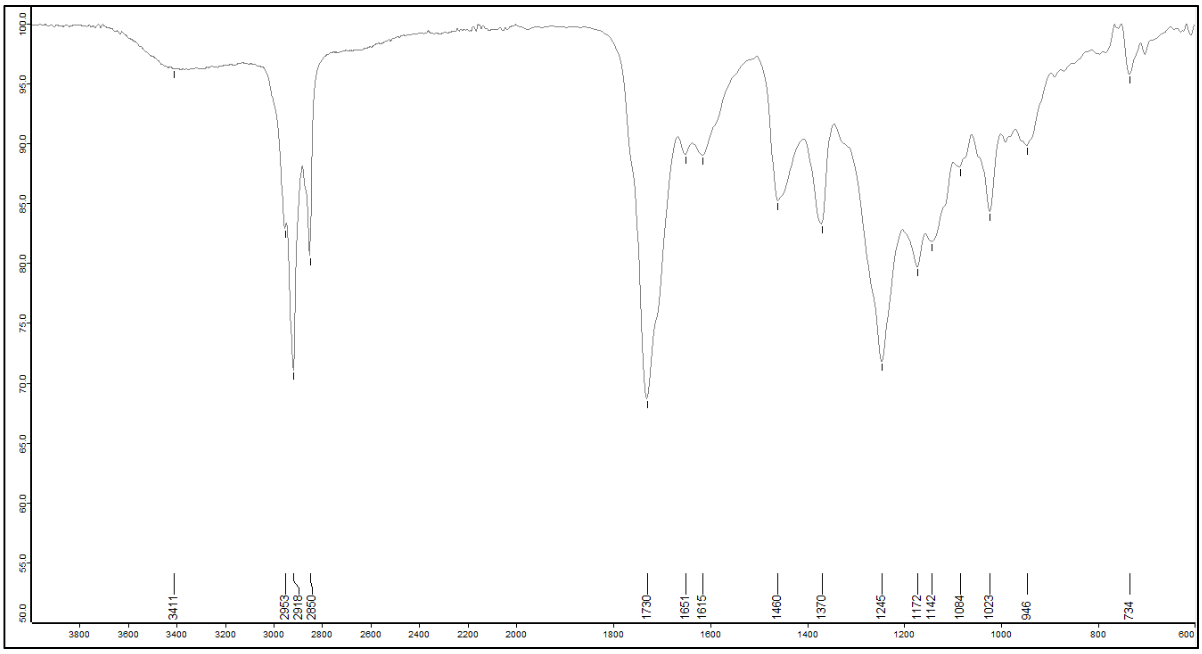

Figure S78: UV spectrum of **7** in methanol.

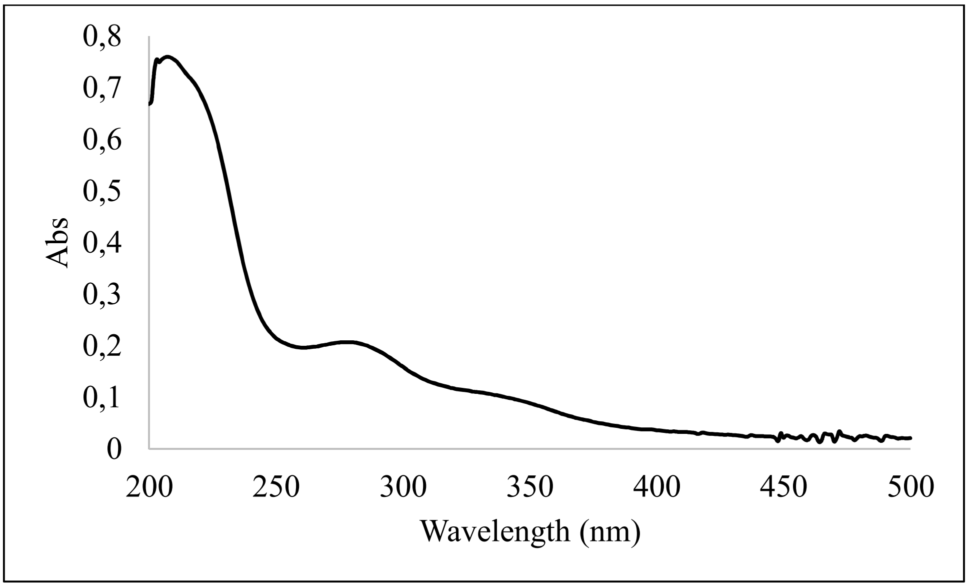

Figure S79: HRESIMS spectrogram of compound **7**.

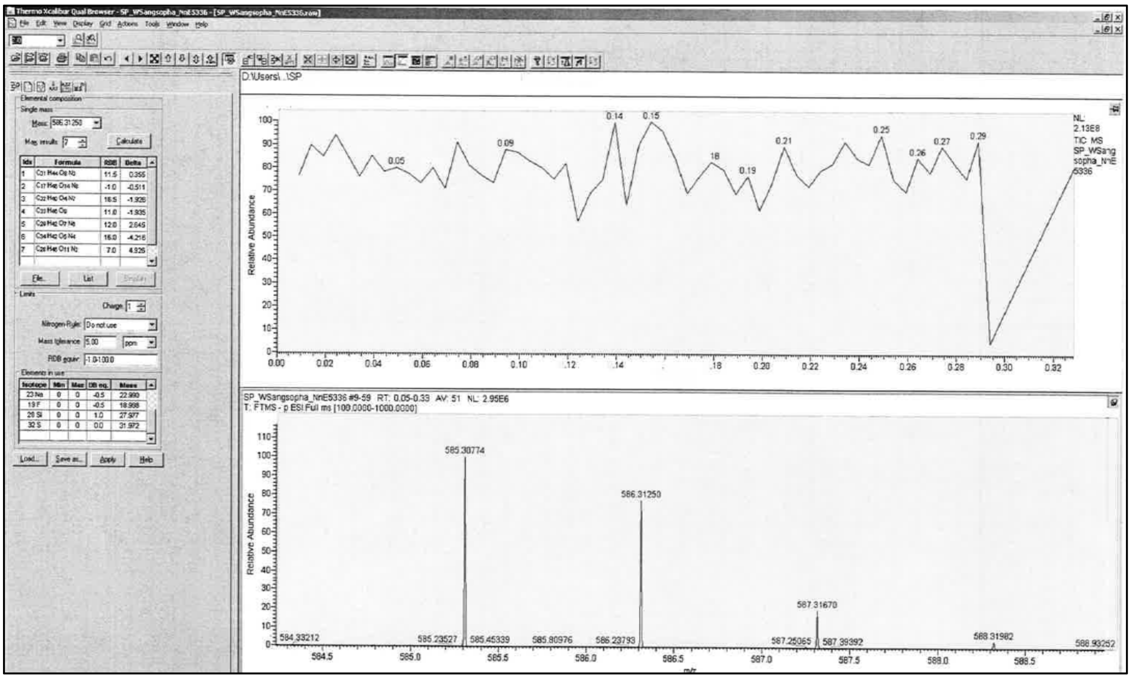

Figure S80: ^{1}H NMR spectrum of compound **7** in CD_3OD.

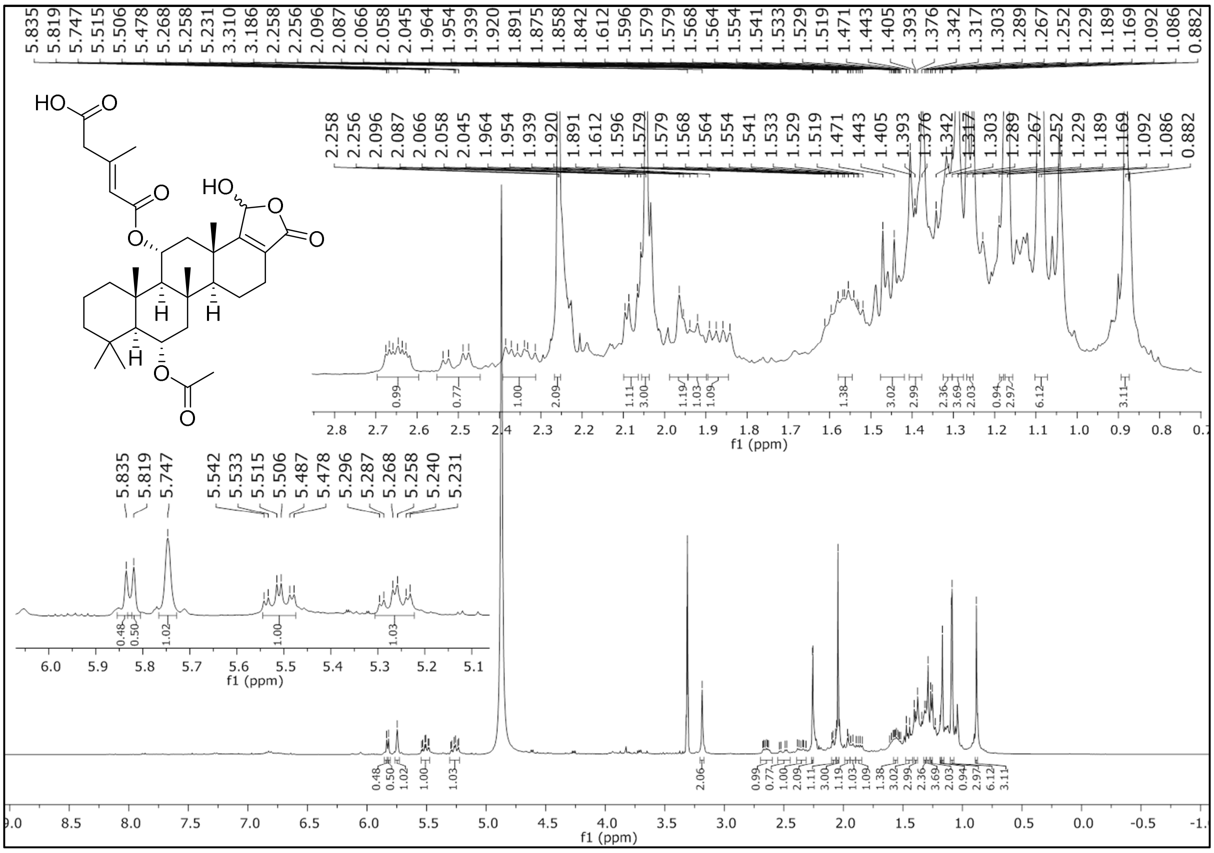

Figure S81: ^{13}C NMR spectrum of compound **7** in CD_3OD.

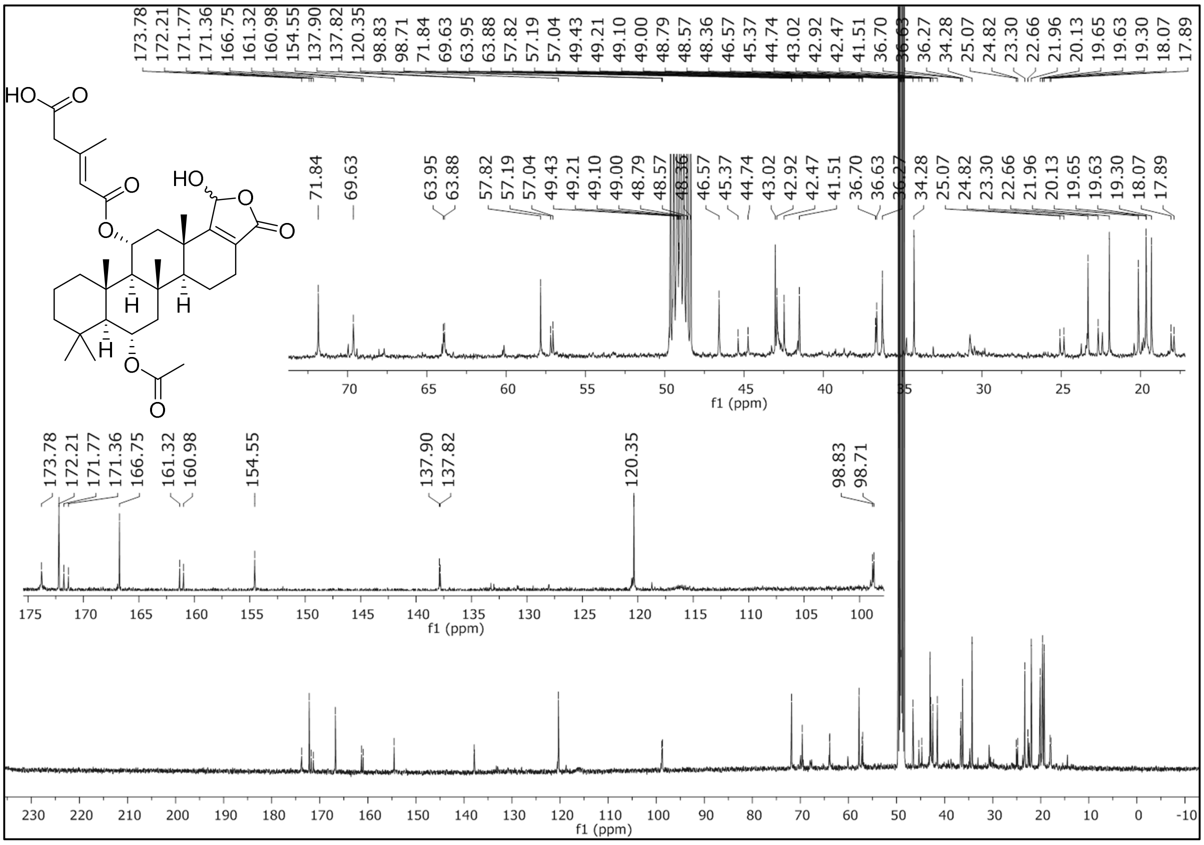

Figure S82: DEPTQ spectrum of compound **7** in CD_3OD.

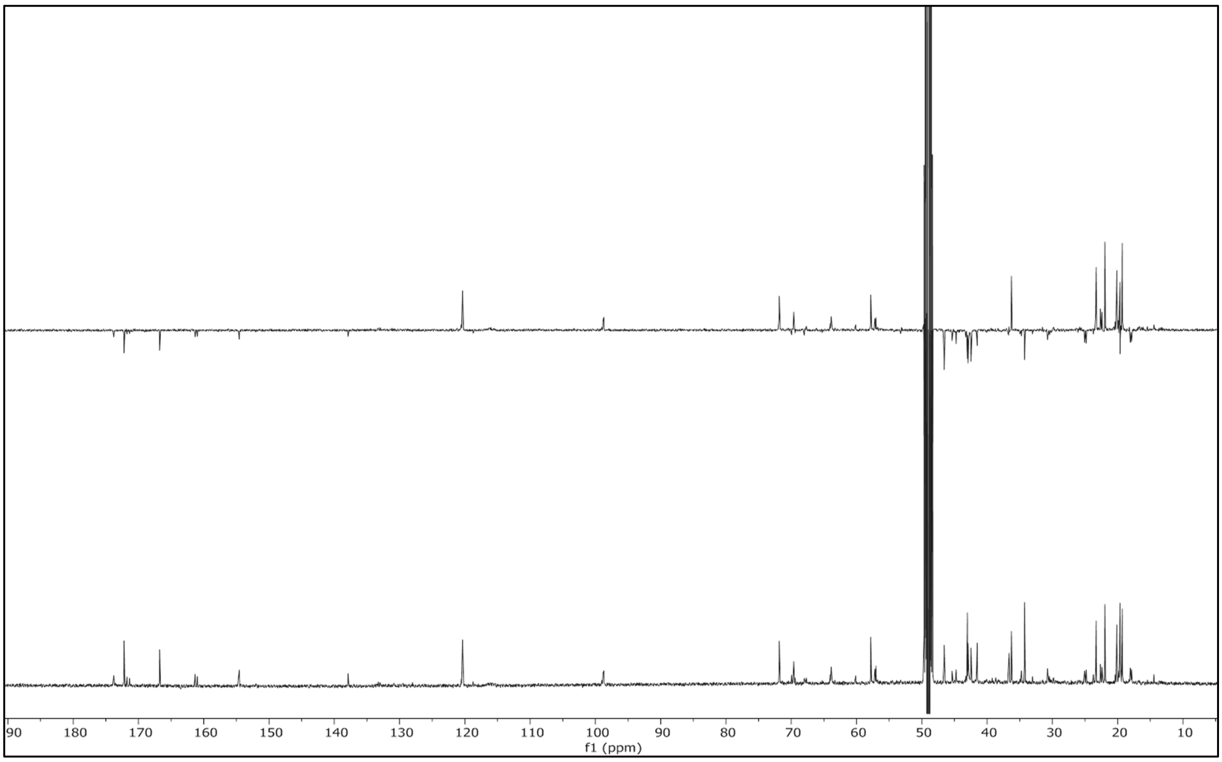

Figure S83: ^{1}H–^{1}H COSY NMR spectrum of compound **7** in CD_3OD.

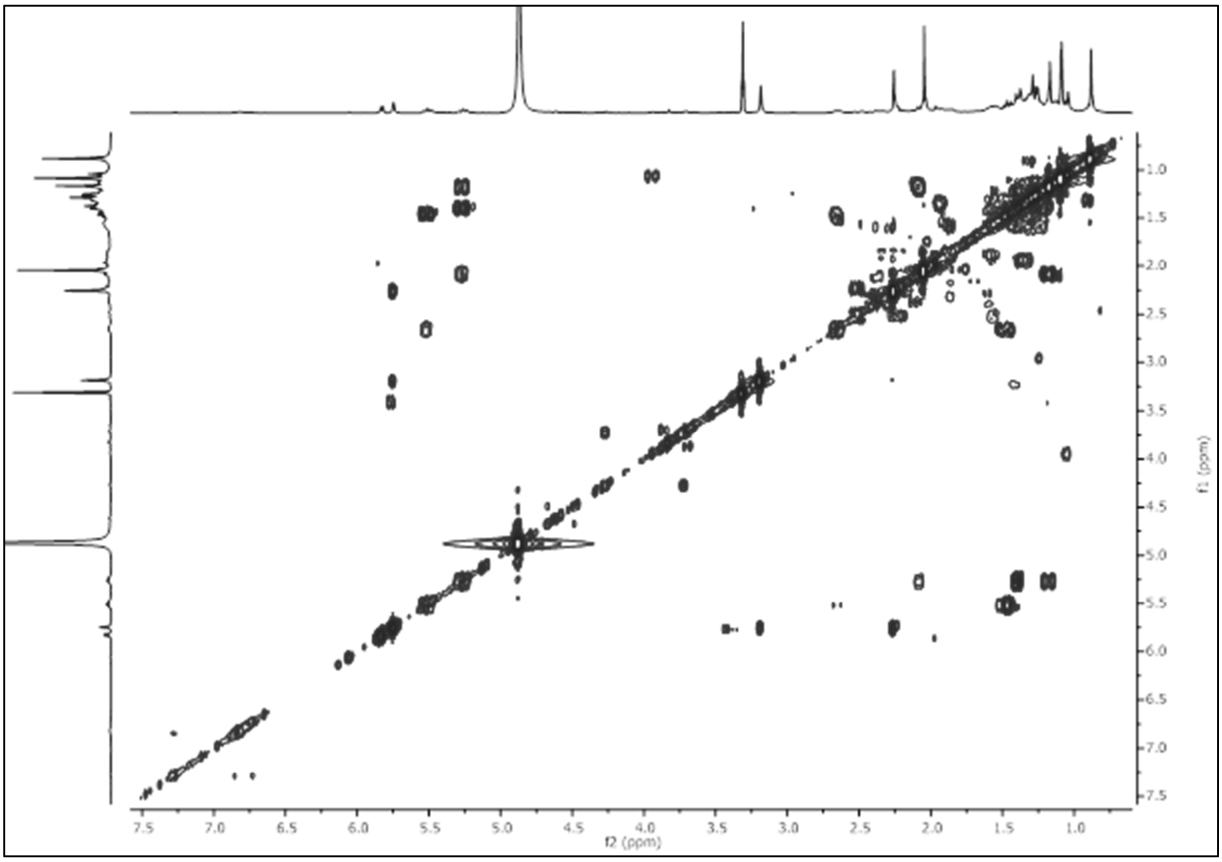

Figure S84: HSQC spectrum of compound **7** in CD_3OD.

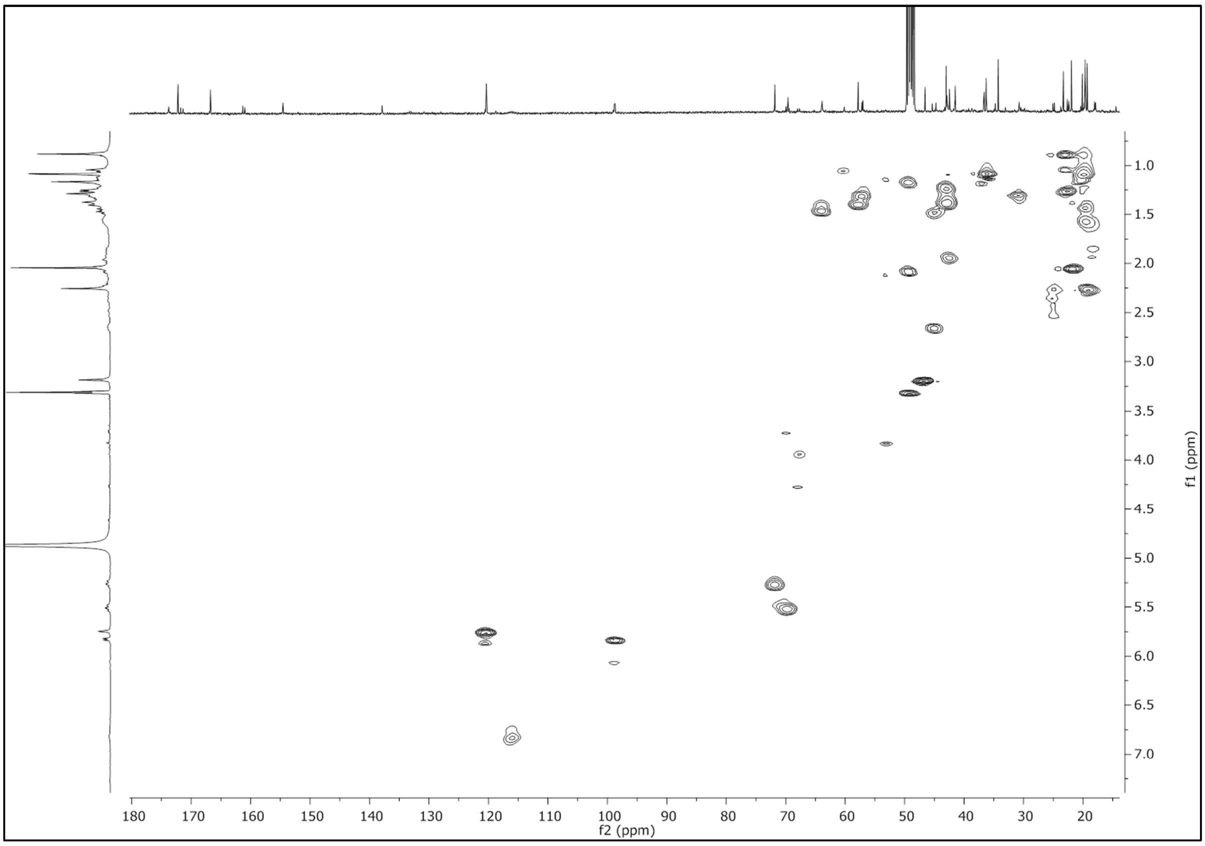

Figure S85: HMBC spectrum of compound **7** in CD_3OD.

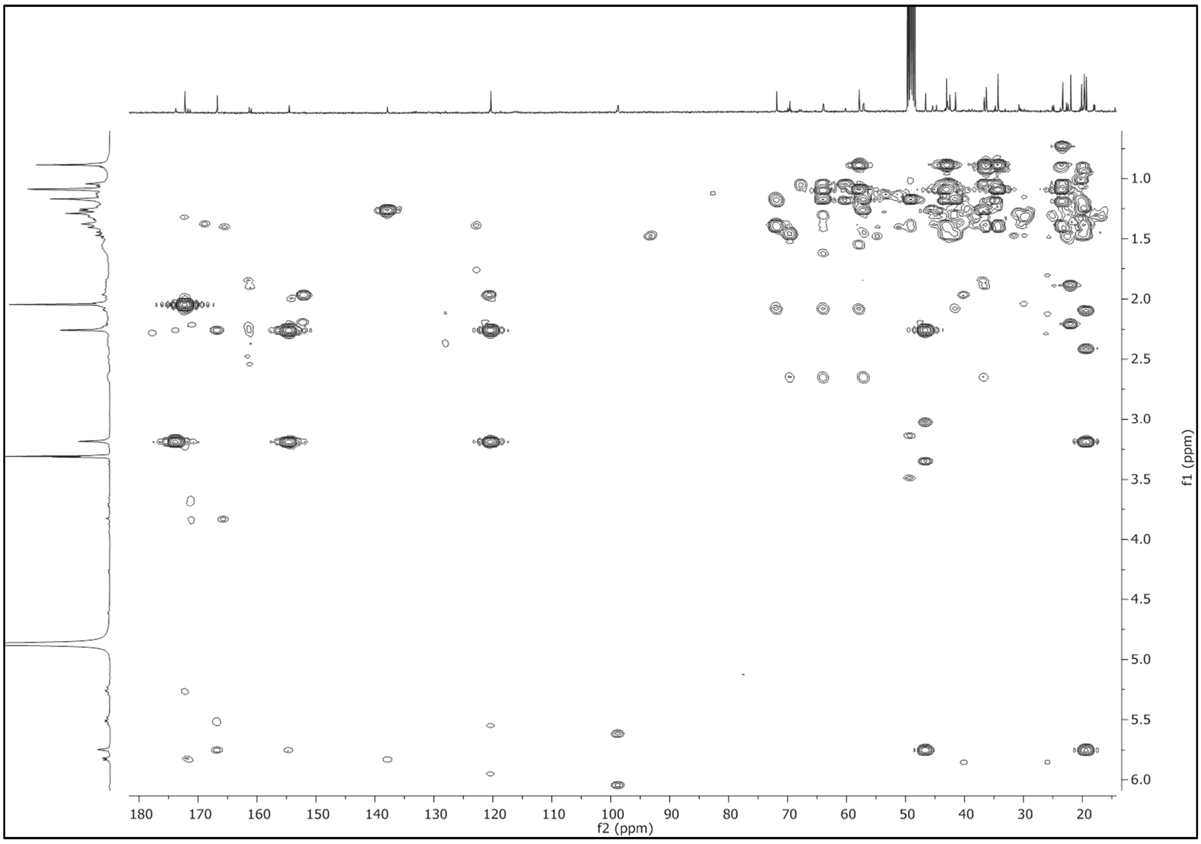

Figure S86: ^{1}H-^{1}H NOESY spectrum of compound **7** in CD_3OD (overview).

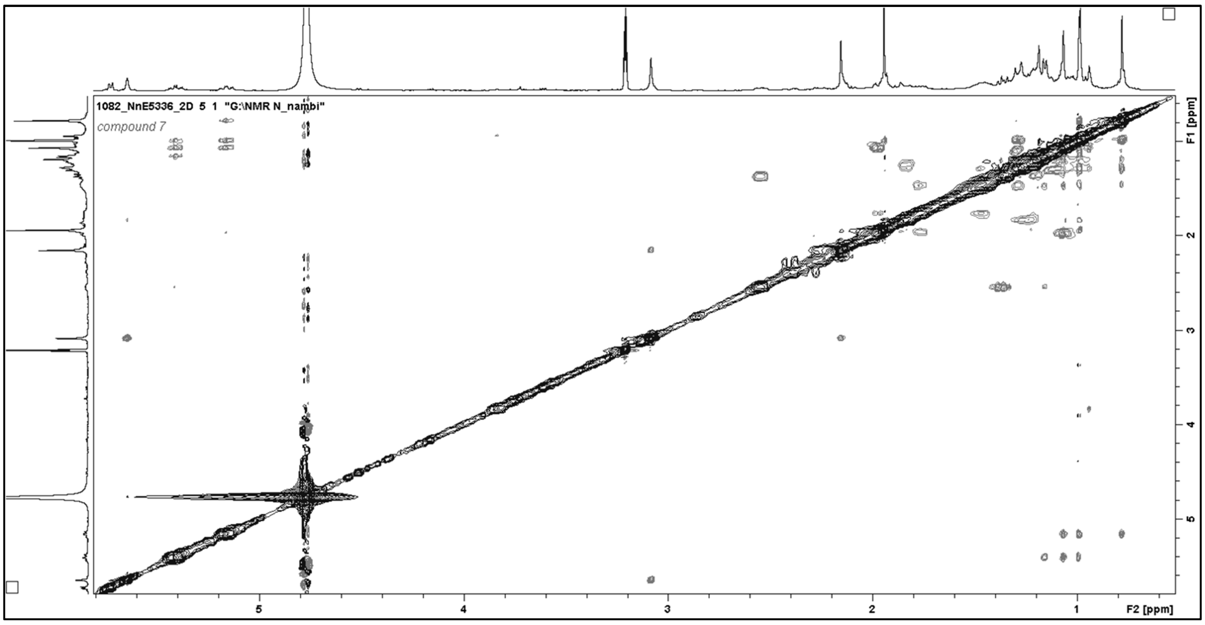

Figure S87: ^{1}H-^{1}H NOESY NMR spectrum of compound **7** in CD_3OD (expanded view of the 0.0–3.0 ppm region).

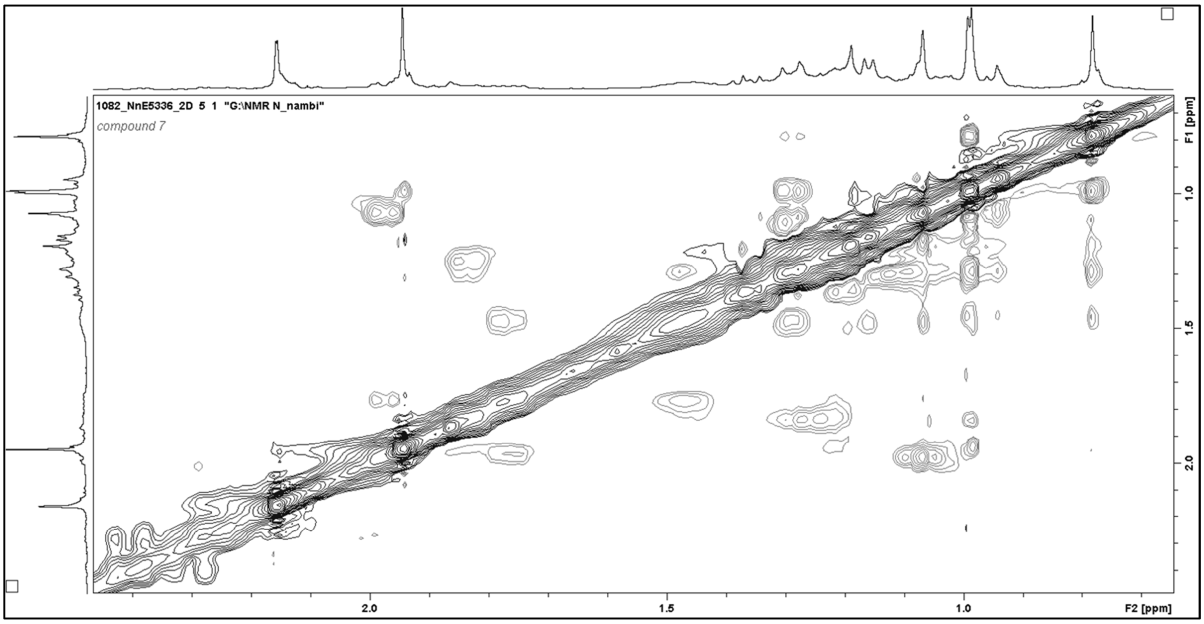

Figure S88: UV spectrum of **8** in methanol.

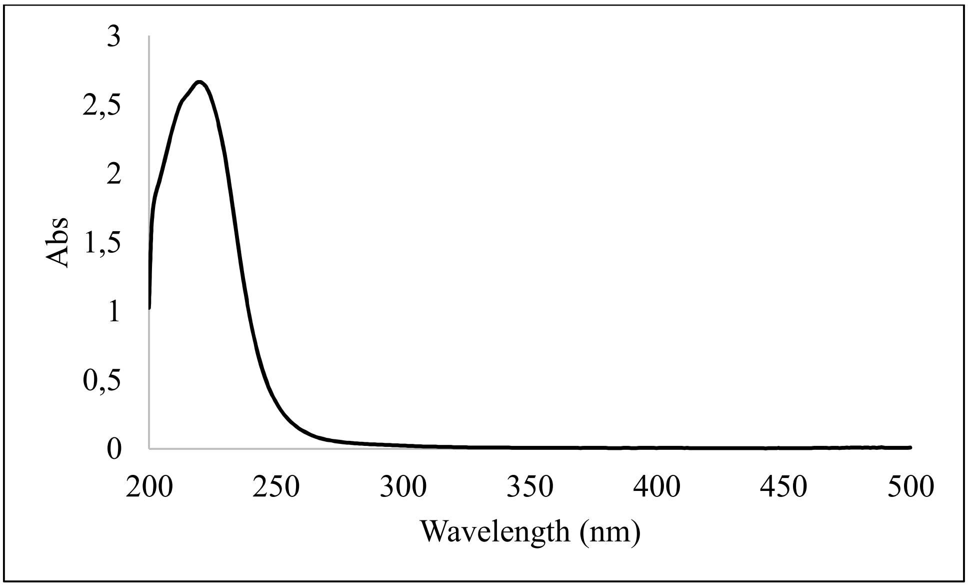

Figure S89: HRESIMS spectrogram of compound **8**.

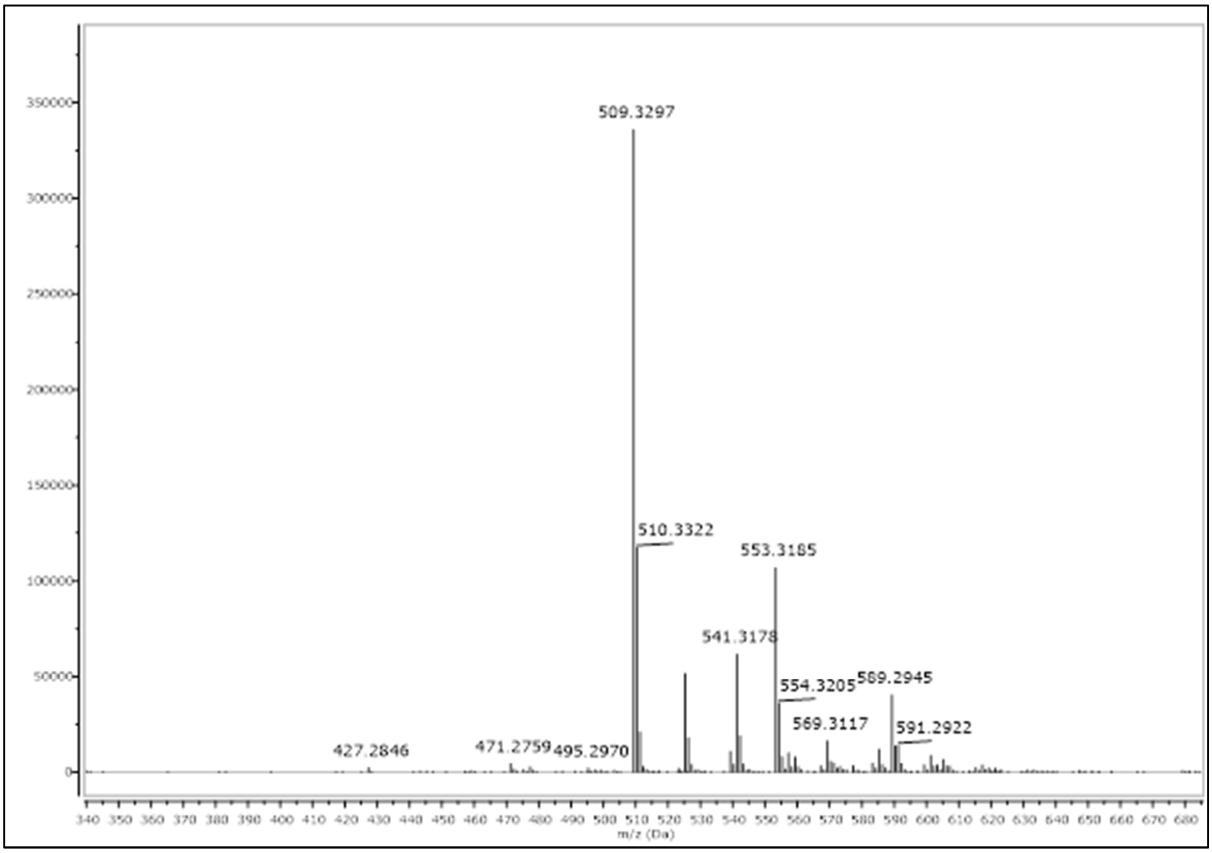

Figure S90: ^{1}H NMR spectrum of compound **8** in $CDCl_3$.

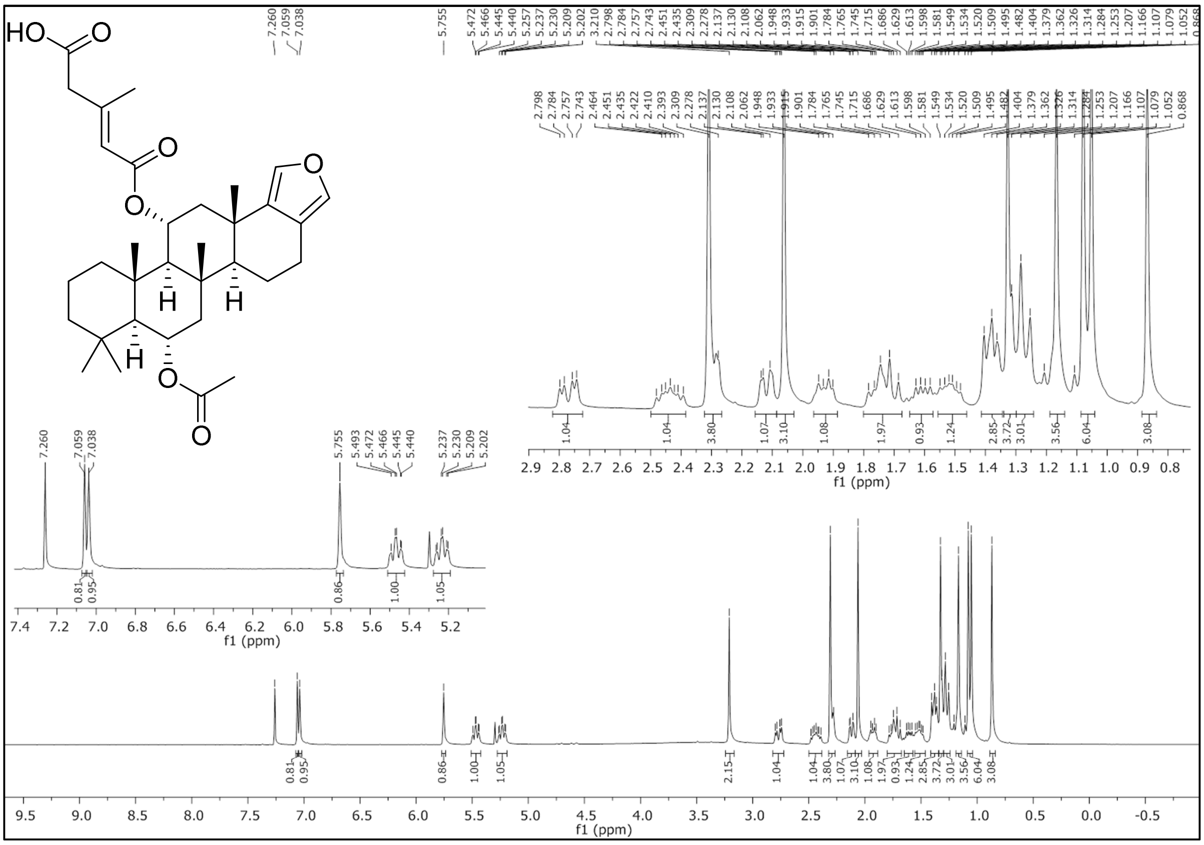

Figure S91: ^{13}C NMR spectrum of compound **8** in $CDCl_3$.

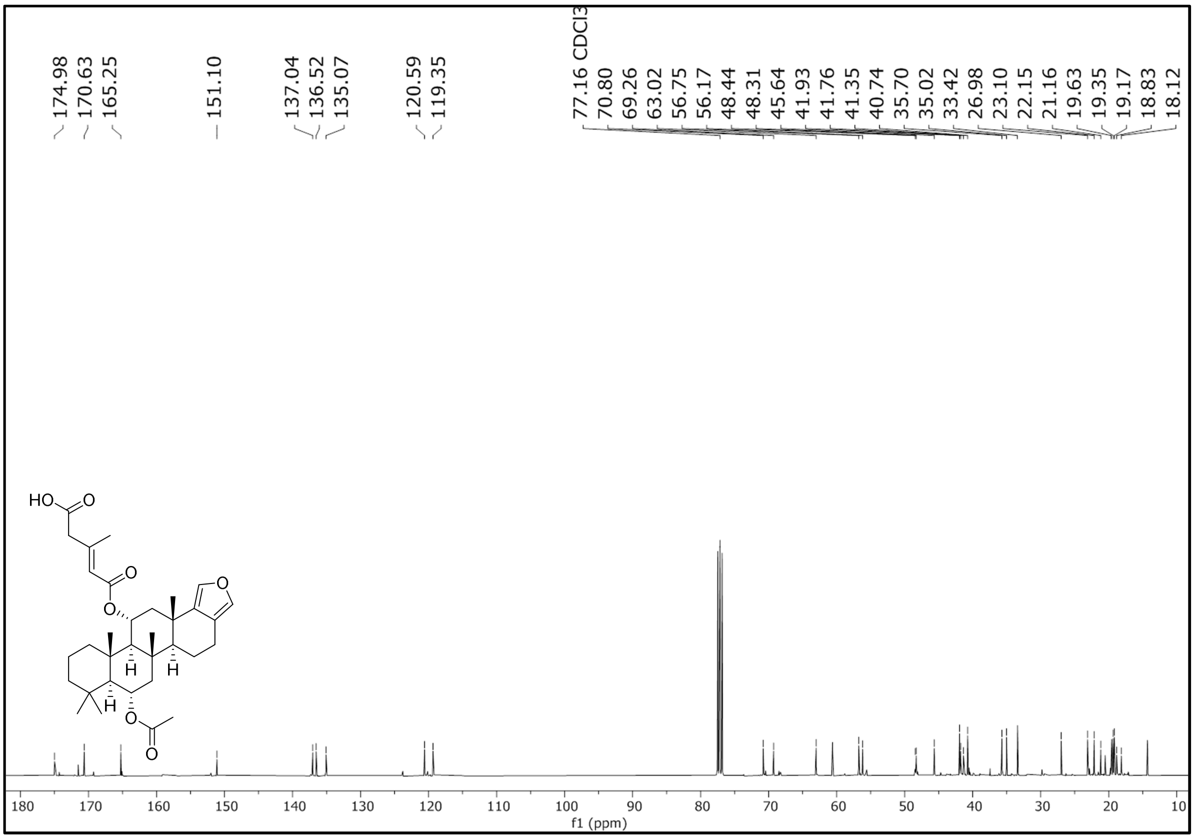

Figure S92: ^{1}H–^{1}H NOESY spectrum of compound **8** in $CDCl_3$ (overview).

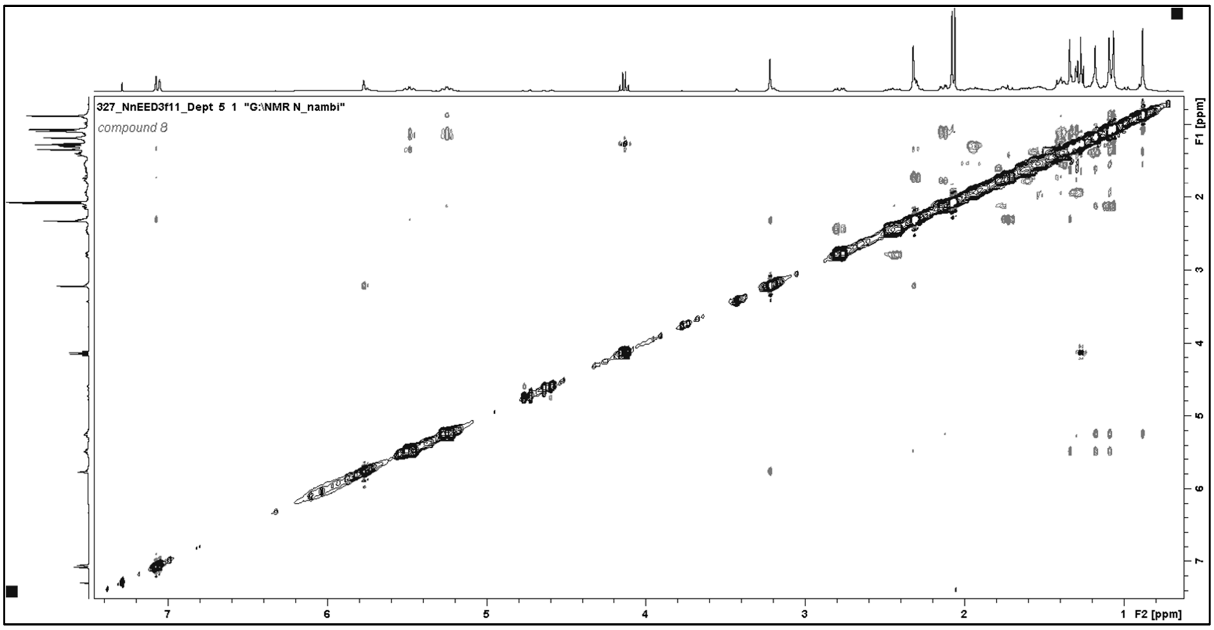

Figure S93: ^{1}H–^{1}H NOESY NMR spectrum of compound **8** in $CDCl_3$ (expanded view of the 0.0–6.0 ppm region).

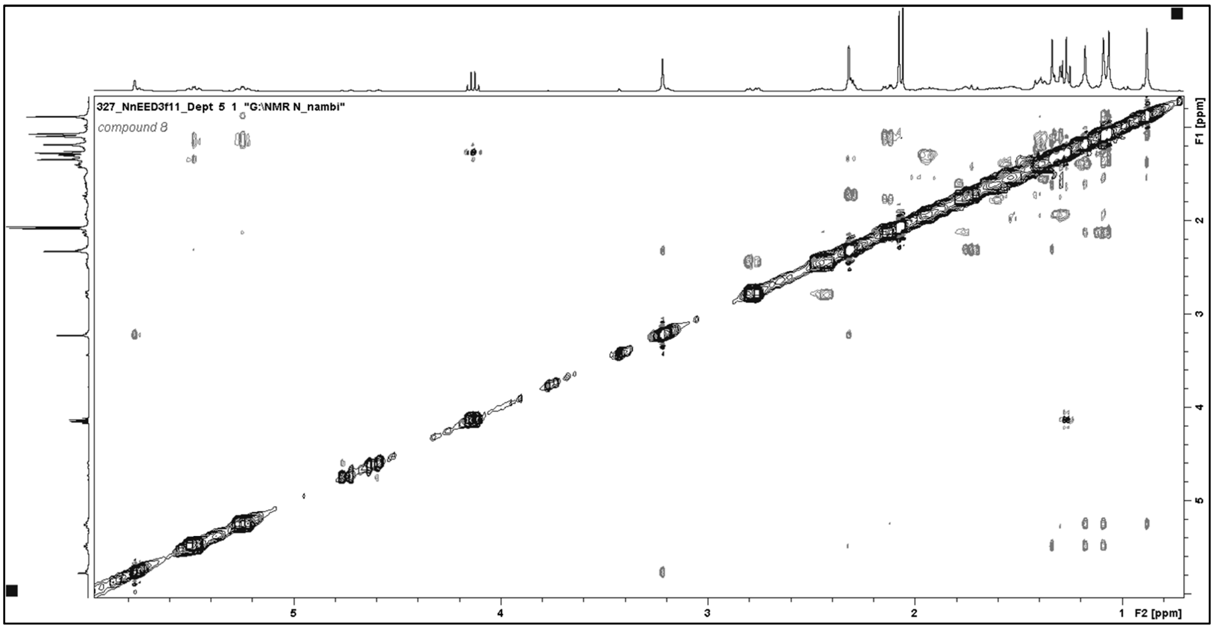

Figure S94: ^{1}H>^{1}H NOESY NMR spectrum of compound **8** in $CDCl_3$ (expanded view of the 0.0–3.5 ppm region).

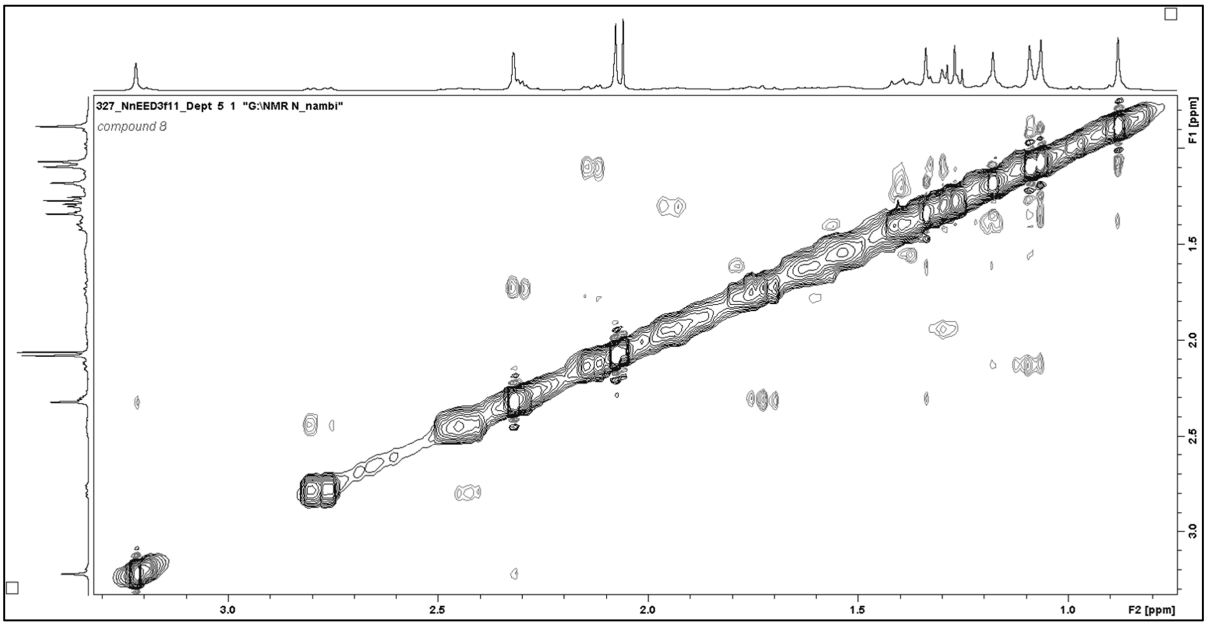

Supplement: Supplementary file 1 [file molecules-26-07667-s001.zip › molecules-1514393-supplementary.pdf]
